# Iterative and data-driven ortholog mining enables reliable discovery of stereoselective ketoreductases

**DOI:** 10.1038/s41467-026-77715-6

**Published:** 2026-09-15

**Authors:** Peter Stockinger, Michael Niklaus, Kevin M. Yar, Nicolas Imstepf, Ivana Pastierikova, Jasmin Küng, Steven Hanlon, Hans Iding, Sumire Honda Malca, Rebecca Buller

**Affiliations:** 1 Department of Chemistry, Biochemistry and Pharmaceutical Sciences, University of Bern, Bern, Switzerland; 2 Institute of Chemistry and Biotechnology, Zurich University of Applied Sciences, Wädenswil, Switzerland; 3 Department of Biotechnology, Delft University of Technology, Delft, The Netherlands; 4 Process Chemistry and Catalysis, F. Hoffmann-La Roche Ltd., Grenzacherstrasse 124, Basel, Switzerland; 5 Present Address: Process Chemistry and Catalysis, F. Hoffmann-La Roche Ltd., Grenzacherstrasse 124, Basel, Switzerland; 6 Present Address: Omics-OS, Inc., 995 Market Street, San Francisco, USA; 7 Present Address: Lonza, Drug Product Services, Hochbergerstrasse 60, Basel, Switzerland; 8 Present Address: RGCC International, Baarerstrasse 95, Zug, Switzerland

**Keywords:** Oxidoreductases, Protein function predictions, Biocatalysis

## Abstract

Ketoreductases (KREDs) enable stereoselective alcohol synthesis and are widely used industrially. Yet identifying process-compatible and selective enzymes for non-native substrates remains challenging, requiring extensive screening. Here, we present an iterative ortholog mining strategy to explore functional space. Starting from a known KRED, we sample its orthogroup across 48 evolutionarily distant variants, identifying five enzymes producing pharmaceutically relevant alcohols (*de*/*ee* 42.8% to >99%). Refined sampling of 60 phylogenetically close orthologs yields further improved ketoreductases (*de*/*ee* 98% to >99%). Because ortholog mining is genome-dependent, it overlooks enzymes from unsequenced organisms. To address this, we use our dataset to explore homologous KREDs beyond the sampled orthogroup, expanding the search space fifteenfold. To this end, we develop the automated pipeline HomoLogic, whose functional descriptors, fed into interpretable models, predict KRED performance accurately (R² up to 0.76). Overall, we show that evolutionarily balanced ortholog panels combined with data-driven modeling enable efficient discovery of stereoselective biocatalysts.

## Abstract (web summary)

Ketoreductases (KREDs) enable the stereoselective synthesis of chiral alcohols and are used in many industrial manufacturing routes, however, the identification of process-compatible enzymes with high activity and selectivity toward non-native substrates remains challenging. Here, the authors present an iterative ortholog mining strategy to efficiently explore and expand functional enzyme space of KREDs.

## Introduction

Ketoreductases (KREDs) occur in various enzyme superfamilies such as aldo-keto reductase (AKR)^1^, short- and medium-chain dehydrogenases/reductase (SDR and MDR) families^2^ and catalyze the reduction of ketones to chiral alcohols with high stereoselectivity using nicotinamide adenine dinucleotide (NAD(P)H) as the hydride donor^3^. Because chiral alcohols are key motifs in pharmaceuticals, agrochemicals, and fine chemicals, KREDs have become widely used in industrial asymmetric synthesis^4^. Unlike traditional ketone reductions, which often require transition-metal catalysts, costly chiral ligands and harsh reaction conditions^5^, KRED-mediated transformations proceed efficiently under mild aqueous conditions and can be paired with cofactor recycling systems, enhancing atom economy and overall sustainability^6^.

KREDs, like many other wildtype enzymes, often exhibit promiscuous activities, such as catalyzing the reduction of non-native ketone substrates. These secondary reactivities, typically not subject to selective pressure unless they affect the host organism’s fitness^7^, can be harnessed for industrial applications^8,9^. Through directed evolution and semi-rational design, enzyme engineering can improve such promiscuous activities, tailoring enzymes with increased catalytic efficiency and stereoselectivity for the non-natural substrates^10^. In addition, enzyme engineering can optimize enzymes’ total turnover and stability under process conditions, such as high organic solvent concentrations or elevated temperatures^10^. Accordingly, various KREDs have been engineered for the industrial synthesis of key chiral intermediates. In an early example, Codexis optimized a KRED from *Lactobacillus kefir* to produce ethyl *(S)*−4-chloro-3-hydroxybutyrate, a key intermediate for the cholesterol-lowering drug atorvastatin (Lipitor)^11^. More recently, Merck & Co. reported a highly active, solvent-stable KRED used in a chemoenzymatic dynamic kinetic reduction cascade to produce a precursor for belzutifan^12^. Further industrial examples include a KRED process reported by Pfizer for an intermediate of an investigational γ-secretase inhibitor for Alzheimer’s disease^13^, the Codexis KRED CDX-026 process for a montelukast intermediate^14^, and a Merck & Co. process for a chemokine receptor inhibitor intermediate^15^, demonstrating the broad industrial relevance of engineered KREDs, which is also reflected in the rapidly increasing number of patent filings describing KRED-enabled processes^4^. Contributing to this body of work, we recently optimized a fungal KRED from *Sporidiobolus salmonicolor* (Ssal-KRED) for the chemo-enzymatic synthesis of a precursor of the AKT inhibitor ipatasertib (compound **1b**)^16–18^. Combining mutational scanning, structure-guided design and machine learning, the optimized variant achieved a 64-fold higher k_cat_, ≥98% conversion, and 99.7% diastereomeric excess (*(R,R)-trans*−**1b**) at a 100 g L⁻¹ scale^16^.

The evolvability of scaffolds such as Ssal-KRED’s motivates the search for related enzymes to expand ready-to-evolve enzyme platform for the scalable production of industrially relevant chiral alcohols. Conventionally, homologous proteins are identified using sequence- or structure-similarity-based approaches such as BLAST^19^, MMseqs2^20^, or Foldseek^21^. However, sequence-based methods can fail to capture distant homologs, whereas structure-guided approaches may identify enzymes with similar folds but vastly different properties, and neither strategy reliably controls functional similarity. Position-specific search tools (as implemented in BioCatNet^22^, EnzymeMiner^23^, RSCB PDB’s 3D motif search^24^, or ActSeek^25^) and cavity-focused approaches (such as Innophore’s point-cloud based catalophore search^26^) address this by mining for candidates with similar catalytic sites or substrate-binding pockets. Their success, however, depends strongly on the quality of the alignments, structural models, or cavity geometries used as inputs, requiring time-consuming refinement cycles and considerable expert knowledge. Moreover, strict binding-site filtering may exclude alternative catalytic solutions arising from distinct topological arrangements of binding-site residues^27,28^.

An alternative, inspired by recent advances in the field of comparative genomics, is to assemble enzyme panels from orthologs. Orthologous enzymes typically retain the same primary function across species that diverged millions of years ago^29^. Yet, owing to distinct evolutionary paths and environmental adaptations, may differ in their secondary activities and substrate scope^8,30^. Using ortholog inference software, such as OrthoFinder^31–33^ or its web version SHOOT^34^, orthogroups of a protein of interest can be defined from the amino acid sequences and genome data of related host organisms^35^. These orthogroups are sets of proteins that descend from a single ancestral gene, including both orthologs and paralogs^36,37^, and are classified based on phylogenetic relationships rather than sequence or structure similarity^29,31^. While preserving a common origin and ancestral function, the evolutionary diversity within an orthogroup can expand the accessible functional landscape, making orthogroups a valuable yet still underexploited resource for biocatalyst discovery^38–40^.

**Fig. 1:**
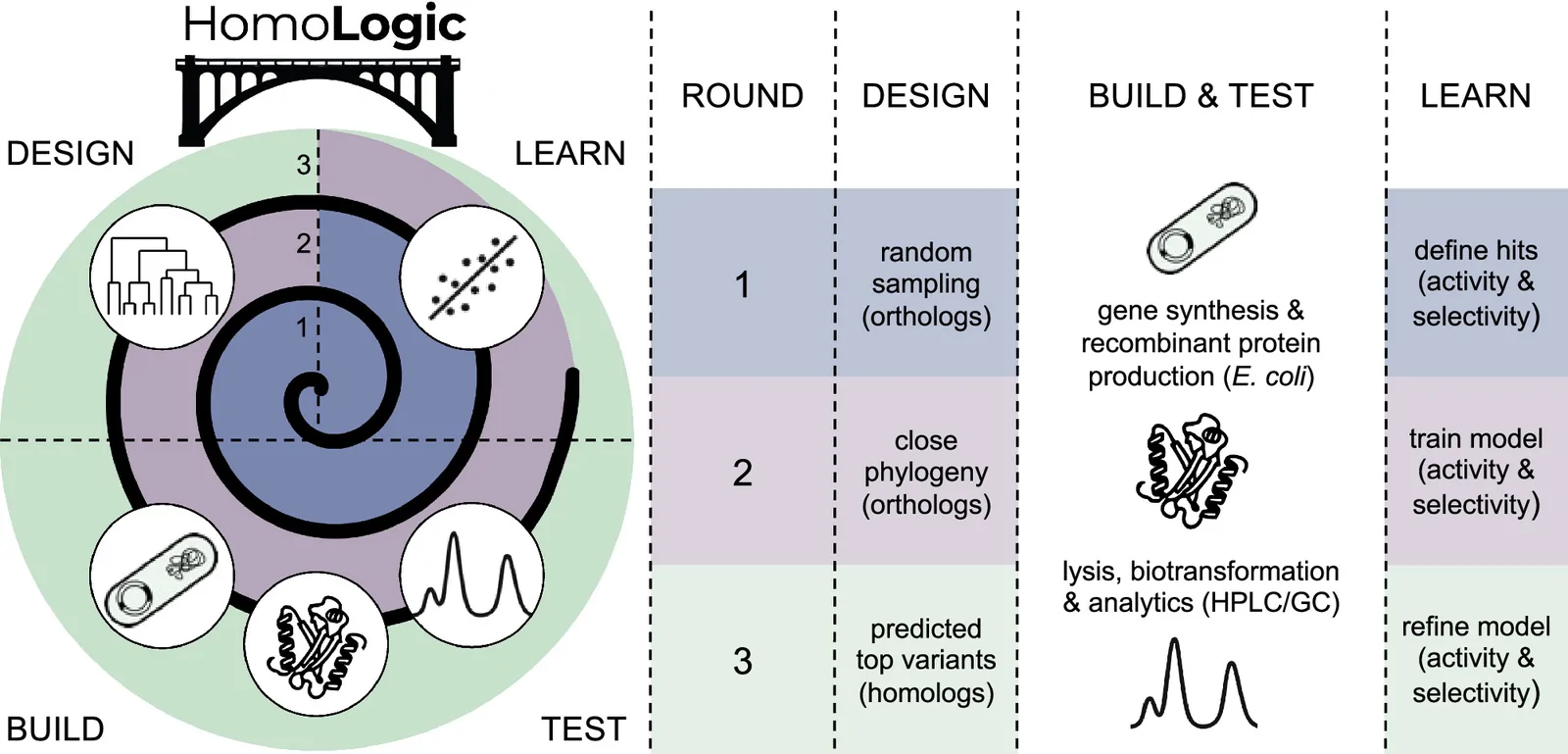
Iterative ortholog mining strategy for expanding the functional landscape of ketoreductases. The suggested data-driven Design-Build-Test-Learn cycle is depicted as an outward-expanding spiral to represent progressive exploration of the sequence space. In Round 1 (blue), the Design phase involves random sampling of orthologs within an orthogroup to generate an initial enzyme library. The Build and Test phases (constant across rounds) comprise gene synthesis, recombinant expression in *Escherichia coli*, and lysate-based biotransformations, with activity and selectivity quantified by HPLC or GC analysis depending on the substrate. The Learn phase in Round 1 identifies initial hits with promising activity and selectivity. These hits guide Round 2 (violet), in which orthologs from phylogenetic proximity are mined to enrich the dataset. To bridge learning from both rounds of ortholog screening and applying the knowledge for data-driven library design, we developed HomoLogic, allowing the extraction of meaningful sequence, structure, and binding-site features for the training of a predictive model. Prospective rounds (e.g., Round 3; green) leverage this model for the targeted identification of functionally similar enzymes across the broader homologous sequence space. In this way, ortholog mining bridges classical comparative genomics and data-driven enzyme discovery, allowing functional homologs to be identified independently of which genomes have been sequenced.

In this work, we present an iterative ortholog mining strategy that explores and expands the functional landscape of ketoreductases. Combining phylogenetic sampling with data-driven refinement, the approach identifies diverse and highly selective biocatalysts while extending the accessible sequence space beyond genomic boundaries. At its core, we introduce HomoLogic, an algorithm that extracts sequence, structure, and binding-site homology features alongside cavity descriptors to bridge conventional genome mining and data-driven discovery (Fig. 1).

## Results

### Round 1: Sampling of Ssal-KRED Orthogroup

**Fig. 2:**
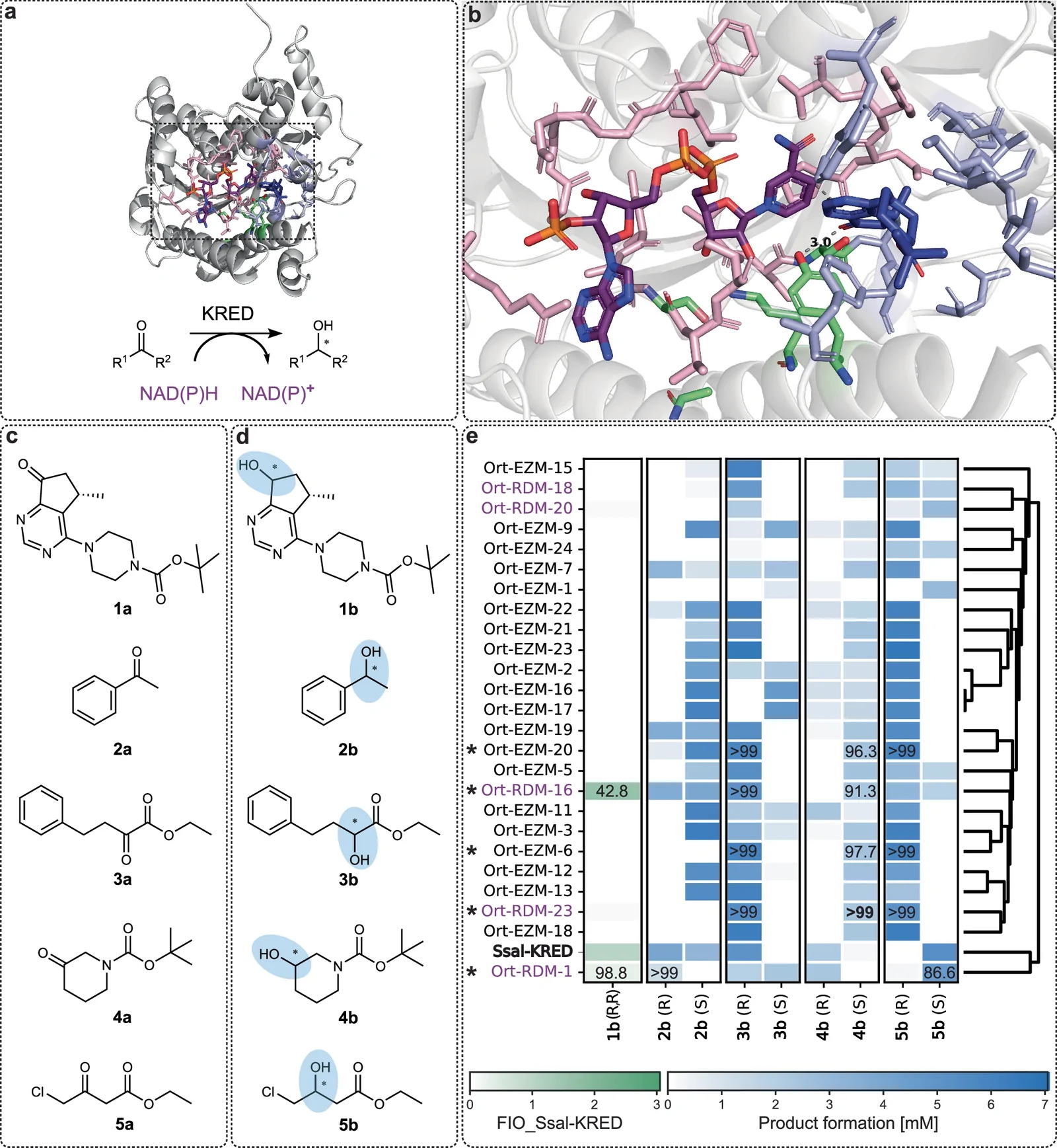
Stereoselective ketoreduction catalyzed by randomly sampled Ssal-KRED orthologs. **a** Depending on the substrate’s orientation relative to the hydride-donating cofactor NAD(P)H, the biocatalytic reduction generates a stereocenter, yielding the corresponding chiral alcohol (blue). The binding-site of one Ssal-KRED monomer is marked with a dashed box in its previously published structural model^16^. **b** Close up view of Ssal-KRED’s binding-site, highlighting catalytic tetrad (light green), substrate binding residues (light blue) and NAD(P)H-binding site (light purple). The NADPH cofactor (dark purple) and substrate **1a** (dark blue) were modeled using PDB 1Y1P and Molecular Operating Environment (MOE 2019.01) in an orientation consistent to hydride transfer from NADPH to **1a** to yield *trans*-*(R,R)*−**1b**. Distance constraints were applied for the interaction of the carbonyl oxygen of **1a** with Tyr177_OH and Ser133_OG (*d* = 2.8 Å) and with the carbon atom of NADPH involved in hydride transfer (*d* = 3.8 Å)^16^. **c** Ketone substrates (**1a**–**5a**) and (**d**) their corresponding alcohol products (**1b**–**5b**) investigated in reduction reactions catalyzed by our panel of KRED orthologs. **e** Functional characterization of the orthologous KRED library sampled in the initial discovery round. Ssal-KRED is highlighted in bold, randomly sampled variants (Set 1, 5 soluble KREDs) in purple, and variants identified via EnzymeMiner (Set 2, 20 soluble KREDs) are depicted in black. Biotransformations were carried out using clarified lysates and 0.1 mg mL^−1^ for **1a** or crude cell extracts and 10 mM **2a**–**5a**. For **1b** formation, the **1a** depletion of each enzyme is normalized to that of Ssal-KRED and displayed as a fold increase over Ssal-KRED (FIO_Ssal-KRED; green color gradient). The blue color gradient depicts the concentration (mM) of the formed *(S)*- and *(R)*- products (**2b**–**5b**), respectively. The background of reactions including lysates from *E. coli* harboring an empty vector has been subtracted from each measurement. The phylogenetic relationship between the characterized scaffolds is represented with a dendrogram (calculated using Biopython’s DistanceCalculator^92^ and SciPy^84^). Top candidates are marked with an asterisk, and enanatiomeric excess (*ee*) of the most selective enzymes are indicated in the respective cells. In cases where values > 99% are given, the opposite enantiomer was not detected. Source data are provided as a Source Data file.

In order to build a library of Ssal-KRED orthologs, we clustered 564 publicly available fungal proteomes from the Ensembl Genomes^41,42^ into orthogroups using OrthoFinder2^32^. Subsequently, the protein identifier of Ssal-KRED (UniProt^43^: Q9UUN9) was utilized to fetch the corresponding orthogroup (Supplementary Fig. S1) from this dataset. As a quality control, we examined the conservation of known functional residues (InterPro^44^ annotation for Q9UUN9) through multiple sequence alignment. Encouragingly, the active-site and NADPH-binding residues (Fig. 2a and b) were highly conserved among orthogroup members, indicating preservation of the canonical catalytic tetrad and cofactor-binding motifs typical of functional KREDs from the Short-chain Dehydrogenase/Reductase (SDR) family^45^. In contrast, substrate-binding residues exhibited considerable variability, suggesting differences in substrate specificity and stereoselectivity (Supplementary Fig. S2).

Orthologs selected for experimental characterization in the first discovery round (Round 1) were grouped into two sets of 24 enzymes (Table 1). For the initial set, we opted to sample randomly from the full orthogroup to maximize sequence diversity (Enzyme nomenclature: Ort-RDM-1 to Ort-RDM-24), while for the second set, EnzymeMiner-filtered candidates with predicted soluble expression were selected (SoluProt^46^ threshold ≥0.75; enzyme nomenclature: Ort-EZM−1 to Ort-EZM-24).

**Table 1:** Library Design of Round 1 Ssal-KRED ortholog library

Set	Strategy	Identifier	N candidates	N solubly expressed
*1*	Random sampling	Ort-RDM-X	24	5
*2*	EnzymeMiner filtering	Ort-EZM-X	24	20
		Total	48	25

All enzyme candidates were ordered as codon optimized gene fragments and cloned into pET22b(+). Recombinant expression in *Escherichia coli* BL21(DE3) was performed under standardized conditions (16 °C, 300 rpm, 30 h), and soluble protein expression was assessed qualitatively by SDS-PAGE (Supplementary Fig. S3). Unsurprisingly, when evaluating the two KRED subsets, pronounced differences in protein expression were observed depending on the mining strategy: the EnzymeMiner-filtered selection showed substantially higher levels of soluble expression (20/24) compared to the randomly sampled set (5/24). However, solubility filtering alone was insufficient for functional enrichment. While three of the randomly selected Set 1 candidates converted the ipatasertib precusor **1a** (Fig. 2c), none of the EnzymeMiner-filtered candidates displayed the desirable activity. In fact, the two most promising candidates, Ort-RDM-1 and Ort-RDM-16 (with solubility scores of 0.47 and 0.62, respectively), would have been excluded by our strictly defined SoluProt threshold of 0.75, underscoring the need to balance selection of sequences correlated with high expression with those contributing functional diversity through careful threshold definition. Post-analysis of our full ortholog dataset (Round 1 & Round 2; *vide infra*) indicated that lowering the solubility threshold to 0.4 would prevent the exclusion of successfully expressed variants (Supplementary Fig. S4).

To further characterize the catalytic potential of the soluble orthologs beyond acceptance of compound **1a**, four structurally diverse ketones were selected to probe the enzymes’ substrate spectra (Fig. 2c): 1-phenylethanone (**2a**), ethyl 2-oxo-4-phenylbutyrate (**3a**), 1-Boc-3-piperidone (**4a**), and 4-chloroacetoacetate (**5a**). 1-phenylethanone (**2a**) represents a well-established model substrate for evaluating the asymmetric reduction capabilities of KREDs, as it contains a small methyl group and a larger phenyl substituent, allowing for assessment of stereoselectivity^9,47^, while the alcohol product *(R)*-**3b** of ethyl 2-oxo-4-phenylbutyrate (**3a)** serves as an intermediate in the synthesis of angiotensin-converting enzyme inhibitors used to treat hypertension and heart failure^48^. A further industrially relevant example is the reduction of 1-Boc-3-piperidone (**4a**) to yield *(S)*-N-Boc-3-hydroxypiperidone (**4b**), a building block in the synthesis of the anticancer drug ibrutinib^49^. Finally, the reduced product *(S)*-**5b** derived from 4-chloroacetoacetate (**5a**) is as a precursor for several pharmacologically active molecules, such as atorvastatin, which is widely used for the treatment of high cholesterol levels (Fig. 2d)^11^.

Biotransformations of substrates **1a** – **5a** were performed using *E. coli* lysates harboring the respective orthologous enzymes, while background reactions were assessed using lysate preparations lacking a target enzyme (empty vector control, EVC). For substrate **1a**, relative activity of the ortholog KRED panel compared to Ssal-KRED was evaluated by recording substrate depletion slopes at 340 nm, allowing us to determine the fold increase over Ssal-KRED (FIO_Ssal-KRED). In this case, the diastereoselectivity of identified hits was verified via liquid chromatography (LC) (*vide infra*). For substrates **2a** – **5a**, chiral gas chromatography (GC) or LC was used to determine enantiomeric excess (*ee*), and product concentrations were quantified by comparison with calibration curves obtained using the corresponding product standards (Supplementary Fig. S5).

Notably, all mined enzymes were active for at least three of the investigated substrates, providing a diverse set of enantiocomplementary biocatalysts for upcoming screening campaigns. Beyond Ssal-KRED, two additional enzymes (Ort-RDM-1 and Ort-RDM−16) were found to efficiently convert **1a**, however with varying diastereoselectivities (*de (R, R)-trans*-**1b** = 98.8% and 42.8%, respectively), while Ort-RDM-23 enantioselectively produced *(R)*-**3b** and *(S)*-**4b** (both *ee* > *99* %). Ort-EZM-6 and Ort-EZM-20 also catalyzed these reductions; however, we found that the reduction of **4a** was not fully *(S)*-selective (*ee* = 97.7% and 96.3%, respectively). Finally, while our query enzyme Ssal-KRED, produced *(S)*-**5b** with excellent enantiomeric excess (*ee* > 99%), within our ortholog panel, only Ort-RDM-1 displayed comparably high selectivity (*ee* = 99.3%).

### Round 2: refined sampling based on Round 1 Hits

To further enrich the pool of active and selective KREDs, a refined set of orthologs in close phylogenetic proximity to the Round 1 hits (Ort-RDM-1, Ort-RDM-16, Ort-RDM-23, Ort-EZM-6, and Ort-EZM-20) was sampled. We defined “hit” as the orthologous KRED with the highest activity and selectivity for each industrially relevant product (*(R,R)-trans*−**1b,** *(R)*-**3b,** *(S)*-**4b**, or *(S)*-**5b**). For each of the five query enzymes, 12 closely related variants were sampled, ensuring diversity by limiting sequence identity to a maximum of 90% (SeqID) relative to the original hit. Of the resulting 60 candidates, 43 (72%) were expressed in soluble form, representing a substantial improvement over the randomly sampled library from Round 1 (Table 2).

**Table 2:** Round 2 library consisting of refined sampling in phylogenetic proximity to Round 1 hits

Set	Hit from Round 1	Identifier in Round 2	N candidates	N solubly expressed
*1*	Ort-RDM−1	Ort-RDM-1_X	12	5
*2*	Ort-RDM-16	Ort-RDM-16_X	12	6
*3*	Ort-RDM-23	Ort-RDM-23_X	12	10
*4*	Ort-EZM-6	Ort-EZM-6_X	12	12
*5*	Ort-EZM-20	Ort-EZM-20_X	12	10
		Total	60	43

X: Candidate identifier within the set.

**Fig. 3:**
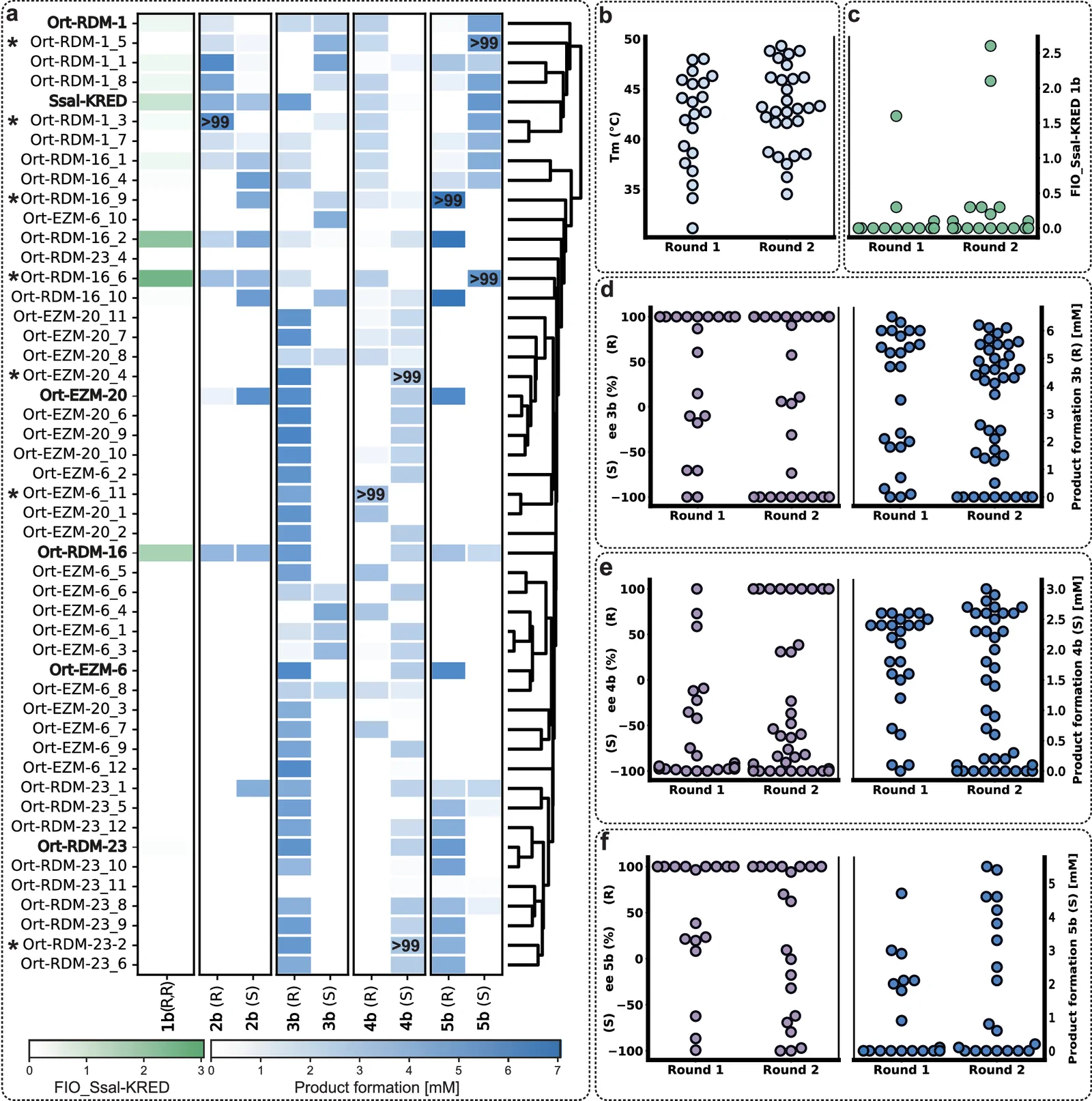
Functional characterization of the orthologous KRED library sampled in the second discovery round. **a** Biotransformations were carried out using clarified lysates and 0.1 mg ml^-1^ for **1a** or crude cell extracts and 10 mM **2a**–**5a**. For **1b** formation, the **1a** depletion of each enzyme is normalized to that of Ssal-KRED and displayed as a fold increase over Ssal-KRED (FIO_Ssal-KRED; green color gradient). The blue color gradient depicts the concentration (mM) of the formed *(S)*- and *(R)*- products (**2b**–**5b**), respectively. The background of *E. coli* expressing an empty vector has been subtracted from each measurement. The phylogenetic relationship between the characterized scaffolds is represented with a dendrogram. Seeds are highlighted with bold font, top candidates are marked with an asterisk and enantiomeric excess (*ee*) values of the most selective enzymes are indicated in the respective cells. In cases where values > 99 % are given, the opposite enantiomer was not detected. Swarm plots are shown to compare variants stemming from discovery Rounds 1 and 2, respectively, in terms of their (**b**) melting temperatures (T_m_) and (**c**) FIO_Ssal-KRED for the conversion of **1b** as well as enantioselectivity (purple) and product formation (dark blue) of (**d**) *(R)*−**3b**; (**e**) (*S*)− **4b**, and (**f**) (*S*)−**5b**. Source data are provided as a Source Data file.

Remarkably, 41 of the 43 soluble orthologs were active toward at least two substrates, and more than half exhibited high enantioselectivity (>95% *ee*) in the formation of their respective target products (Fig. 3a), highlighting the effectiveness of the iterative sampling strategy. Overall, the second sampling round identified ten KREDs that exhibited improved activity and selectivity compared to the Round 1 hits for the formation of *(R,R)-trans*-**1b,** *(S)*-**4b**, and *(S)*-**5b** (Fig. 3b-e; Supplementary Data 3). In addition, our search strategy revealed eight biocatalysts capable of producing the ipatasertib precursor *(R,R)-trans*-**1b**, two of which (Ort-RDM-16_2 and Ort-RDM-16_6) exhibited higher activity than the seed enzyme Ssal-KRED (2.1 and 2.6-fold respectively) while maintaining excellent diastereoselectivtity (*de (R,R)-trans* = 95.9% and 98%, respectively) (Table 3). Interestingly, for all hit enzymes, enhanced activity was accompanied by reduced thermal stability, as reflected by a lower melting temperature (T_m_ between 31.1 °C and 46.8 °C) compared to Ssal-KRED (T_m_ = 59.7 °C) (Table 3). To rationalize these differences in melting temperature, we derived additional structural features (such as solvent accessible surface areas, observed secondary structures, and the frequency of certain non-covalent bonds) with potential relevance for protein stability and analyzed their correlation with the available melting temperatures of all orthologs (Supplementary Fig. S6). This analysis shows that Ssal-KRED stands out with increased local rigidity in the binding-site region and stabilizing secondary structure elements. Specifically, Ssal-KRED ranks highest in median binding-site pLDDT while exhibiting low binding-site pLDDT variability (pLDDT reasonably correlates with conventional flexibility metrics^50^). It also displays the third-lowest fraction of 3₁₀-helices and comparably high bend fractions with greater solvent-exposed surface area (typically α-helices are more stable than 3₁₀-helices while bends are more stable than loops^51–53^). Although the individual correlations between these features and the measured melting temperature were relatively weak (Pearson’s r < 0.4), they reached statistical significance in our dataset (*P* < 0.05; *n* = 54).

**Table 3:** Comparison of **1a** active orthologs with respect to sequence identity (SeqID), melting temperature (T_m_), fold-improvement over Ssal-KRED, conversion and diastereomeric excess (*de*)

Identifier	Round	SeqID^a^ [%]	T_m_ [°C]	FIO_Ssal-KRED^b^	Conversion^c^ [%]	de (R,R)-trans [area %]
*Ssal-KRED*	–	100	59.7	1	39	>99
*Ort-RDM-1*	1	33	31.1	0.34	19	98.8
*Ort-RDM-1_1*	2	41	38.8	0.3	10	97.4
*Ort-RDM-1_3*	2	48	42.7	0.2	8	97.9
*Ort-RDM-1_8*	2	42	38.2	0.26	9	98.4
*Ort-RDM-16*	1	32	46.8	1.6	62	42.8
*Ort-RDM-16_1*	2	28	42.2	0.28	12	95.9
*Ort-RDM-16_2*	2	33	38.3	2.1	83	93.5
Ort-RDM-16_6*	2	29	34.5	2.6	96	98

^a^sequence identity towards Ssal-KRED.

^b,c^Fold-improvement over Ssal-KRED and conversion estimated from UV assay samples containing 300 µM = 0.01% **1a**.

The most active variant is marked with an asterisk.

In total, our iterative ortholog mining approach yielded ten selective (*ee* > 99%) and active (product formation between 2.9 and 7.6 mM) enzymes to form chiral alcohols **2b**–**5b**, with one enantiocomplementary pair identified for each target product (Table 4). Notably, seven of these ten variants originated from the second ortholog sampling round, underscoring the effectiveness of an iterative mining strategy.

**Table 4:** Activity, enantioselectivity (*ee*), and thermal stability (T_m_) of top performing and enantiocomplementary biocatalysts for products **2b** – **5b** compared to the seed enzyme Ssal-KRED (blue font)

Identifier	Round	SeqID^a^ [%]	T_m_ [°C]	Product	Product form. [mM]	Conversion^b^ [%]	ee [area %]
*Ort-RDM-1_3*	2	48	42.7	**2b** *(R)*	5.8	58	99.7 *(R)*
*Ort-EZM-3*	1	30	37.6	**2b** *(S)*	6.3	63	99.7 *(S)*
*Ssal-KRED*	seed	100	59.7	**2b** *(R)*	4.1	41	12.5 *(R)*
*Ort-EZM-23*	1	26	41.1	**3b** *(R)*	6.5	65	>99 *(R)*
*Ort-EZM-17*	1	29	47.9	**3b** *(S)*	4.9	49	>99 *(S)*
*Ssal-KRED*	seed	100	59.7	**3b** *(S)*	2.4	24	10 *(S)*
*Ort-EZM-6_11*	2	27	42.7	**4b** *(R)*	3.4	34	>99 *(R)*
*Ort-EZM-23_2*	2	31	47.4	**4b** *(S)*	3.0	30	99.2 *(S)*
*Ort-EZM-20_4*	2	30	43.2	**4b** *(S)*	2.9	29	>99 *(S)*
*Ssal-KRED*	seed	100	59.7	**4b** *(R)*	2.2	22	82.7 *(R)*
*Ort-RDM-16_9*	2	30	36.2	**5b** *(R)*	7.6	76	>99 *(R)*
*Ort-RDM-16_6*	2	29	34.5	**5b** *(S)*	5.5	55	>99 *(S)*
*Ort-RDM-1_5*	2	38	49.3	**5b** *(S)*	5.4	54	>99 *(S)*
*Ssal-KRED*	seed	100	59.7	**5b** *(S)*	5.3	53	>99 *(S)*

^a^sequence identity towards Ssal-KRED.

^b^conversion to respective product.

### Sequence, structure and substrate binding-site diversity

**Fig. 4:**
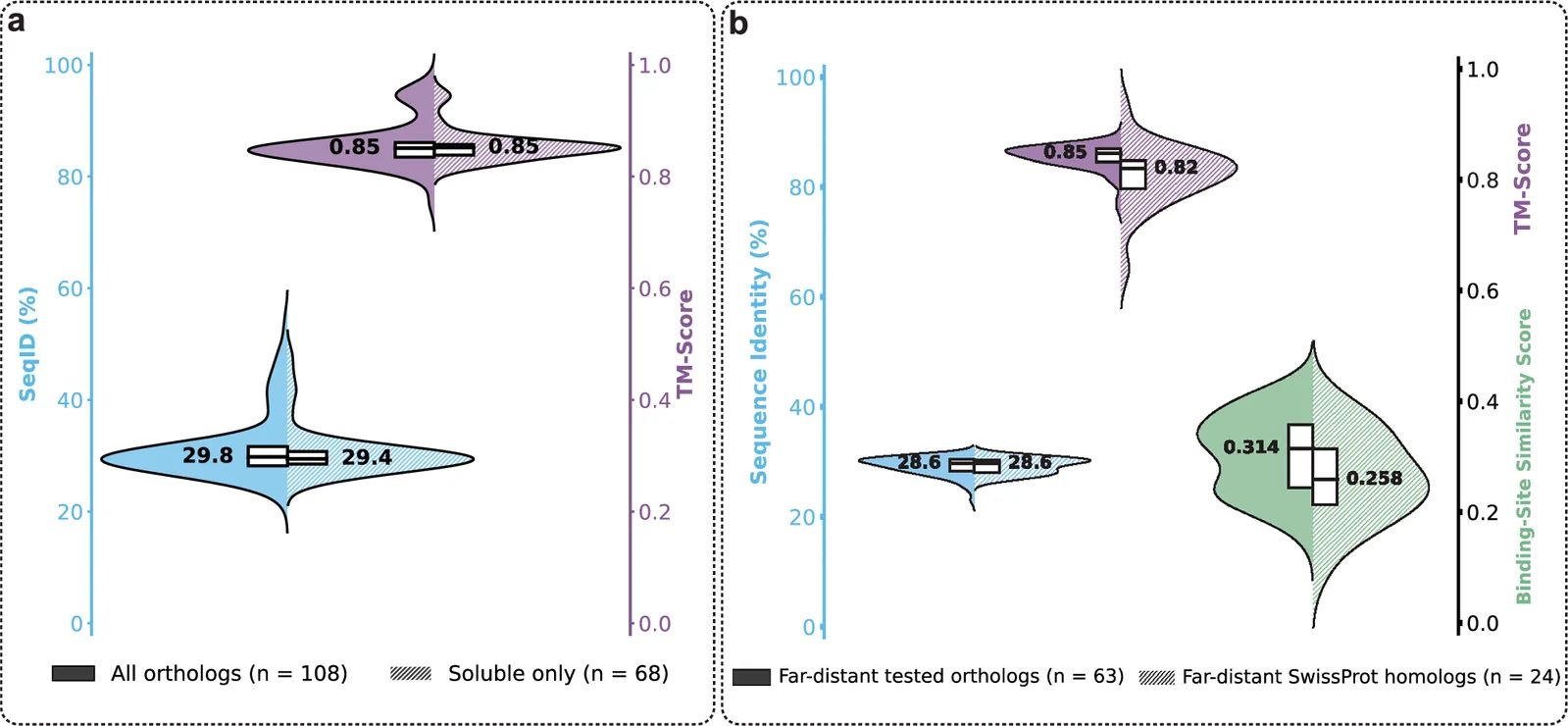
Sequence, structure, and binding-site similarity distributions. **a** Sequence and structure similarity toward Ssal-KRED distributions measured using MMseqs2’s^20^ sequence identity (light blue) and TM-align’s^54^ TM-score (purple) for all orthologs from both discovery rounds in comparison to the soluble expressed subset (hatched filling). **b** Far-distant orthologs (SeqID <= 30%) display significantly shifted structure and binding-site similarity distributions toward Ssal-KRED compared to far-distant SwissProt homologs (SeqID <= 30%). Within each split violin, a white box plot marks the median (horizontal line with text annotation) and the interquartile range (25th–75th percentiles). The median of all distributions is specified by a bold label next to the box plot within the violin representation. Source data are provided as a Source Data file.

In total, the final KRED library (consisting of round 1 and 2 orthologs) comprised 108 candidates, 69 of which (64%) were annotated at the time of writing as hypothetical, putative, or uncharacterized proteins. Although all sequences evolved from a common ancestor, the median sequence identity toward Ssal-KRED was 30% only (range: 22–54%), indicating that our sampling strategy captured sequences that would likely be overlooked by approaches solely relying on high sequence identity. In contrast, the orthologs displayed robust structural similarities with a median TM-score of 0.85 (range: 0.75–0.98), as calculated using TM-align^54^ (Fig. 4a). To contextualize these findings, we compared both TM-scores and sequence identities of all far-distant orthologs (SeqID <30%) to an equivalent set of Ssal-KRED homologs from the SwissProt database. While the SeqID distributions were similar between the two groups (unpaired two-tailed Welch’s t-test), orthologs exhibited significantly higher average TM-scores than SwissProt homologs (0.84 vs. 0.80; *P* < 0.01) (Supplementary Table S2 and S3).

Intrigued by the elevated structural similarity of Round 2 orthologs to Ssal-KRED, we next explored whether this trend also extended to other molecular descriptors. Because orthologous proteins are, by definition, expected to retain the same primary function (even at very low sequence identity)^55^, we hypothesized that geometry, residue composition, and physicochemical properties of the substrate binding-site would show stronger conservation in orthologs than in non-orthologous homologs. This expectation aligns with previous work demonstrating that structural and physicochemical features of protein binding sites carry meaningful functional information^56,57^. Of note, the orthologs exhibited significantly higher binding-site similarity between each other compared to the set of homologs (Fig. 4b; average score 0.31 vs. 0.26; *P* = 0.02), while SwissProt homologs showed larger predicted cavities (mean volume 631 Å³ vs. 512 Å³; *P* = 0.02) composed of fewer aromatic and more negatively charged residues (both *P* ≤ 0.01) (Supplementary Fig. S7 and Supplementary Table S3). While the differences between the two sets were statistically significant, this does not necessarily establish a causal relationship between orthology and the observed binding-site features. We also note that the datasets differ in size (*n* = 63 orthologs vs. *n* = 24 homologs), which may influence statistical power.

### Harnessing ortholog screening data for ML-driven enzyme discovery

A major limitation of using ortholog mining for enzyme discovery is that tools such as OrthoFinder strictly depend on genome data^31,35^. As a result, the accessible natural diversity is limited to those enzyme sequences present in sequenced genomes^58^. The extent of this limitation can be illustrated by the orthogroup of Ssal-KRED, which was derived from only 565 available fungal genomes (Supplementary Data 1) and contained only 4181 orthologs/paralogs (Supplementary Data 2). In contrast, a sequence-based similarity search (MMseqs2 easy-search against UniProt) identified nearly 70,000 sequences that are homologous to our tested orthologs (sharing ≥25% SeqID), highlighting a vast and largely untapped sequence space. Although not all these sequences are expected to share the same function, at least a subset is likely to represent true orthologs that are simply not associated with a sequenced genome (such as those derived from metagenomic sequencing)^58^.

To address this limitation, we propose an iterative enzyme-mining strategy in which ortholog screening serves as an initial exploration of sequence space. The resulting sequence-activity and sequence-selectivity data pairs can then be leveraged by data-driven approaches, such as machine learning (ML), to identify additional promising candidates within the much larger pool of homologous sequences lacking associated genome data. In fact, the characteristics of our ortholog dataset make it particularly well-suited for such data-driven enzyme discovery. Despite its limited size (*N* = 69; 68 of 108 orthologs successfully expressed, plus Ssal-KRED) and generally low sequence identity to Ssal-KRED, the dataset displays consistently high structural conservation (*vide supra*), a narrow length distribution (mean 339 ± 13 residues), and low redundancy (76% singletons at an >80% identity threshold; Supplementary Fig. S8). These features minimize preprocessing challenges, reduce the risk of trivial data leakage, and ensure that each example provides relatively independent, high-quality information^59–61^.

To deal with the limited sample size while maximizing predictive power and interpretability, two complementary strategies seem viable: (a) fine-tuning of protein language models (PLMs) such as ESM-2^62^ or ProtT5^63^, which can leverage learned representations but require careful regularization to avoid overfitting on small n^64,65^; or (b) simple, interpretable predictors (such as LASSO-regularized^66^ multiple linear regression) based on a minimal set of meaningful features to prioritize biological insight over complexity and mitigate overfitting risks^59–61,67^.

**Fig. 5:**
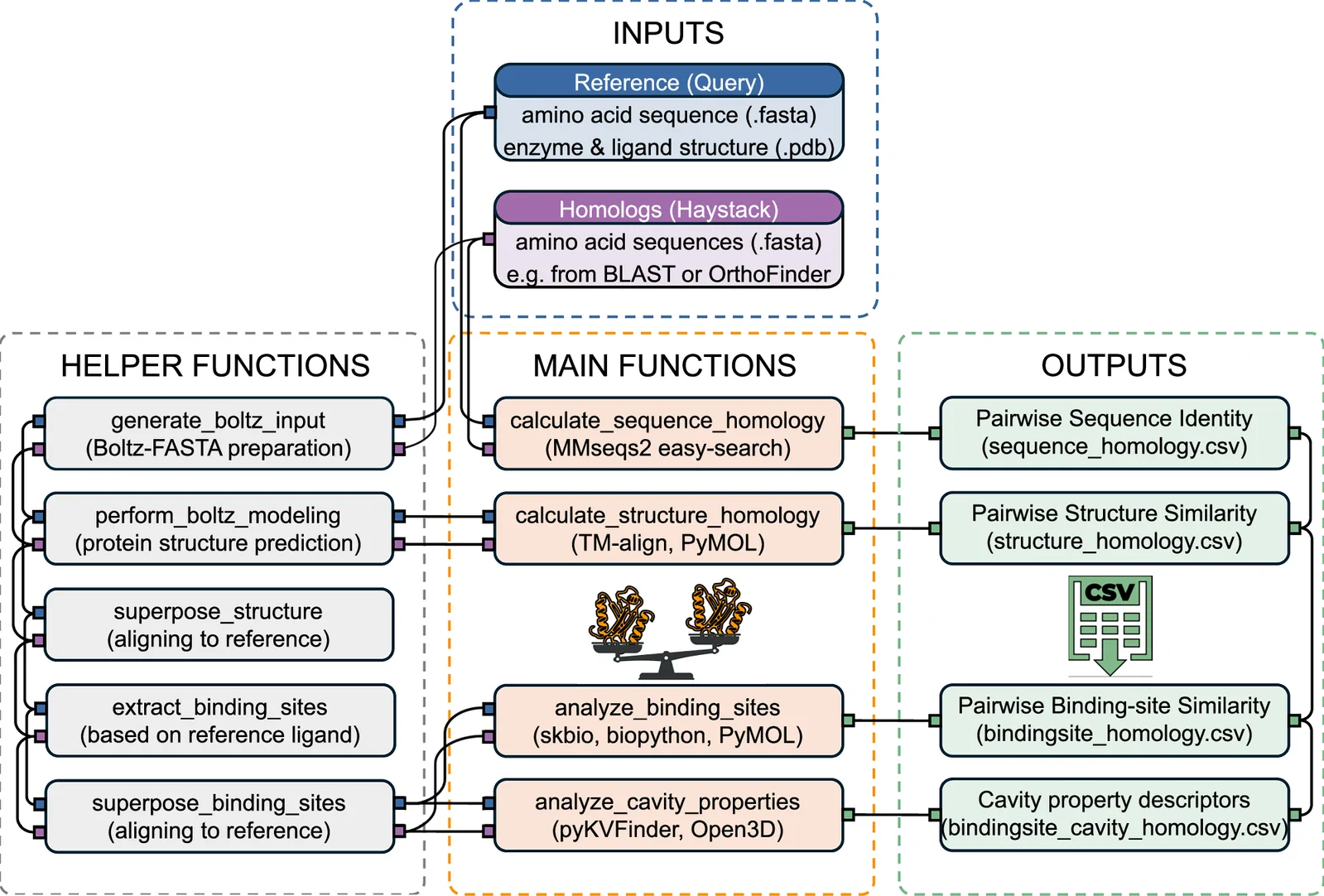
Schematic overview of the HomoLogic-workflow. HomoLogic output table can serve as a feature set for statistical analyses (e.g., correlation analysis) or machine learning. Inputs are shown in the blue dashed box in colored text fields (reference field is blue and homologs field is purple). Helper functions, main functions and outputs are displayed in gray, orange and green, respectively. All logical connections between inputs and outputs are displayed via connecting black lines.

Inspired by the considerably increased structure and binding-site similarity that we observed when comparing our orthologs to SwissProt homologs (*vide supra*), we decided to explore the second approach. To extract evolutionarily and mechanistically meaningful features from our ortholog dataset, we opted to comparatively analyze amino acid sequences, structure models, and substrate binding-sites relative to a reference structure (in our case Ssal-KRED). For this purpose, we developed HomoLogic, a fully automated computational pipeline for functional homology quantification within large sets of enzyme sequences (available at https://github.com/Buller-Lab/HomoLogic). HomoLogic leverages sequence comparisons (MMseqs2^20^), large-scale co-folding to create protein-ligand complex models (Boltz-2^68,69^), structural alignments (TM-align^54^ and PyMOL^70^), cavity detection and analysis (pyKVFinder^71^), as well as binding-site residue mapping to compute a comprehensive set of functional descriptors. These include global measures, such as SeqID, TM-score, RMSD, but also binding-site-specific descriptors, such as binding-site similarity metrics, cavity geometry and physicochemical descriptors (Fig. 5).

**Fig. 6:**
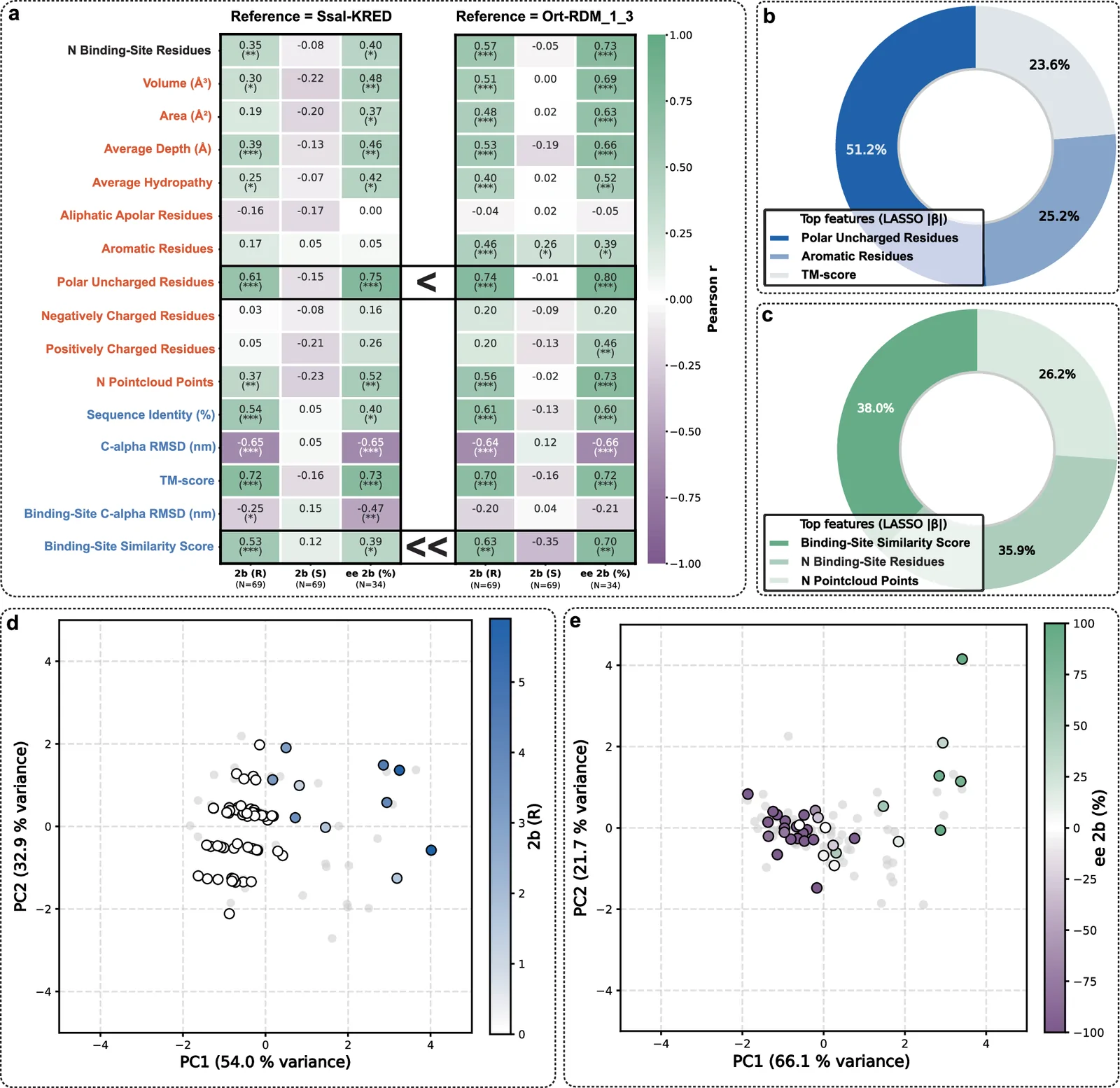
Identification of meaningful features for biocatalytic activity toward 2a. **a** Pearson correlation analysis of all orthologs’ activity and selectivity against model substrate **2a** and HomoLogic-derived descriptors calculated using Ssal-KRED and Ort-RDM-1_3 as reference, respectively. General cavity descriptors and homology metrics relative to the selected reference are colored orange and blue, respectively. Pearson r for the frequency of polar uncharged residues (in contact with the cavity) and the binding-site similarity score (relative to the according reference) are highlighted with a black box. Sample sizes used to derive statistics are defined per column, with *n* = 34 independent samples for *ee* **2b** (%) and *n* = 69 independent samples for all remaining columns. Statistically significant correlations are indicated on the heatmap as follows: * *p* < 0.05, ** *p* < 0.01, and *** *p* < 0.001. *(S)*-selectivity is shown with negative values. Feature importance of top three predictors for (**b**) product formation of **2b** ***(****R)* and (**c**) enantioselectivity were selected by LassoCV (5-fold cross-validation on standardized features) and utilized to train an ordinary least-squares multiple linear regression model. Principal component analysis (PCA) of these three features confirms their relevance to distinguish all orthologs based on their (**d**) product formation of **2b** *(R)* and (**e**) enantioselectivity. Not successfully expressed variants were still considered in the PCA and displayed as faded gray scatters. Source data are provided as a Source Data file.

Initially, we investigated the predictive performance of HomoLogic-derived cavity descriptors such as cavity volume or amino acid frequencies utilizing the previously modeled^16^ complex of Ssal-KRED, NADPH and **1a** as reference. Encouragingly, individual features (such as the frequency of polar residues around the cavity) showed moderate to strong correlations with enzymatic activity across multiple substrates (Supplementary Fig. S9a). These correlations appeared especially pronounced for product **2b** *(R)* (up to Pearson r = 0.75, *P* < 0.001; Fig. 6a).

Calculated values of these descriptors are expected to vary depending on the utilized reference protein structure and ligand coordinates because HomoLogic superposes all query structures onto the reference to ensure a common coordinate space and defines binding sites based on the ligand input (with an adjustable cutoff, 6 Å by default). Beyond general cavity descriptors, HomoLogic also calculates a diverse set of sequence-, structure-, and binding-site similarity metrics relative to a selected reference. To take full advantage of these metrics, the choice of reference should ideally be aligned with the intended prediction task. For example, when building a predictor for the enantioselectivity toward **2a** and the product formation of **2b** *(R)*, a model of the best-performing variant in complex with **2a** (Ort-RDM-1_3) should be used as a reference. To investigate the predictive performance towards **2b** *(R)* and its *ee*, we accordingly modeled Ort-RDM-1_3 in complex with NADPH and **2a** using Boltz-2. The HomoLogic*-*derived descriptors were correlated with the experimental observables (Supplementary Fig. S9b). As anticipated, some descriptors (such as the binding-site similarity score) calculated relative to the reference displayed substantially stronger correlations to the formation of **2b** *(R)* and the product’s *ee* than compared to when Ssal-KRED was used as a reference (Pearson r improved from 0.54 to 0.69 and from 0.39 to 0.75, respectively; Fig. 6a).

After showing that the HomoLogic-derived features are meaningful using single-feature correlations, we explored their combined potential in simple, interpretable predictive models for stereochemical outcomes. We applied a two-step approach: (1) LassoCV (5-fold cross-validation on standardized features) for automatic variable selection, followed by (2) ordinary least-squares multiple linear regression (OLS) limited to the top three features selected by Lasso to preserve statistical robustness given the modest sample sizes. For product formation of 2b *(R)* (*n* = 69 variants with soluble expression), Lasso retained three biologically plausible features, yielding a model with solid explanatory power (R² = 0.658, adjusted R² = 0.642, RMSE = 0.842; Supplementary Fig. S10a). The leading predictors were the frequency of polar uncharged residues in the cavity (β = +0.475), the frequency of aromatic residues in the cavity (β = +0.233), and the global structural similarity to the reference enzyme Ort-RDM-1_3 as captured by TM-score (β = +0.219) (Fig. 6b). Leave-one-out cross-validation (LOO-CV) confirmed good internal predictivity (Q² = 0.592, RMSE = 0.920, MAE = 0.697, PCC = 0.770) with only mild overfitting (Δ(R²-Q²) = 0.066), indicating that the model generalizes reasonably well within the ortholog dataset despite the limited number of training examples. The models consistently selected cavity-composition features (e.g., polar uncharged and aromatic residue) alongside global structural similarity, allowing mechanistic interpretations. Given that **2b** is the aromatic ketone 1-phenylethanone, the positive influence of aromatic residues in the active site likely reflects π-stacking interactions with the phenyl ring, while uncharged polar residues may facilitate hydrogen bonding with the carbonyl oxygen, anchoring the substrate in a productive orientation. That such chemically intuitive features emerge from the selection underscores the capacity of the descriptors to capture functionally relevant differences in active-site polarity, π-stacking potential, and overall structural fold.

Encouragingly, modeling **2b** enantioselectivity (*n* = 34 variants with measurable stereopreference) revealed an even stronger predictive performance using the same two-step pipeline (LassoCV followed by OLS on the top three features), achieving R² = 0.761 (adjusted R² = 0.737, RMSE = 34.419 %; Supplementary Fig. S10b). The model selected three highly relevant descriptors: the binding-site similarity score relative to the reference Ort-RDM-1_3 (β = +21.905), the total number of binding-site residues (β = +20.691), and the number of points in the cavity point cloud (β = +15.104) (Fig. 6c). LOO-CV confirmed excellent internal predictivity (Q² = 0.712, RMSE = 37.814, MAE = 30.795, PCC = 0.844) with minimal overfitting (Δ(R²-Q²) = 0.049), indicating robust generalization within this smaller but functionally informative subset of orthologs. Notably, the selected features differ markedly from those dominating product formation of **2b** *(R)*. For enantioselectivity, the dominance of binding-site-specific metrics is mechanistically intuitive: increased binding-site similarity to the highly selective reference (99.7 % *(R)*) likely preserves a pocket geometry that enforces a consistent substrate orientation (e.g., carbonyl face exposure favoring one enantiomer in a Prelog- or anti-Prelog-like manner^47^).

To probe generalization to unseen sequence space, we performed redundancy reduction via MMseqs2 clustering at sequence identity thresholds of 90%, 80%, 70%, 60%, and 50% retaining one centroid representative per cluster, followed by re-evaluation through LOO-CV using the same fixed three-feature models. Because the retained representatives are mutually dissimilar below each threshold, every held-out sequence lacks a close homolog in the training and can be used to assess generalizability. Predictive performance remained remarkably stable despite the progressive removal of homologous sequences (Supplementary Tables S4-S5; Supplementary Fig. S11) and, notably, the absolute predictive accuracy was retained: Q² for 2b product formation and ee (%) remained ≥ 0.525 down to 60% identity, compared with Q² = 0.59 at 90% identity. Consistently, the R²–Q² gap, our measure of overfitting, stayed below 0.14 for *ee* **2b** (%) down to 15 representatives (50% identity) and below 0.12 for **2b** (R) production down to 36 representatives (60% identity). We did not cluster below 50% identity, as the number of remaining datapoints became too low for reliable LOO-CV. This robustness is consistent with our featurization strategy, which deliberately exploits structural and binding-site homologies rather than raw sequence similarity to identify promising enzymes. Although sequence identity was available as a candidate feature, it was not selected among the top three by LASSO in either model; instead, the models relied primarily on structural (TM-score) and binding-site similarity. Because predictions are therefore driven by structural and active-site relatedness rather than sequence identity, the models retain accuracy even when close sequence homologs are removed, a behaviour expected to generalize toward distant homologs.

To further substantiate the relevance of the derived feature sets, we applied principal component analysis (PCA) (an unsupervised dimensionality reduction technique) to the top three Lasso-selected features for each target, independent of experimental labels. This analysis tests whether the selected descriptors inherently organize the ortholog variants in a manner that reflects their stereochemical performance.

For product formation of **2b** *(R)* (*n* = 109, including not expressed variants), PCA on the top three features captured 86.9 % of the total variance in two dimensions (PC1: 54.0%, PC2: 32.9%). When points were colored according to measured product formation levels, a clear gradient emerged along PC1 (Fig. 6d). The strongest contributions to PC1 originated from the frequency of polar yet uncharged binding residues (+0.703) and TM-score (+0.686). For **2b** enantioselectivity (*n* = 109, including not expressed variants), PCA on binding-site similarity score, number of binding-site residues, and number of points in cavity point-clouds explained 87.7 % of the variance (PC1: 66.1%, PC2: 21.7%). Coloring by *ee* produced a pronounced separation along PC1, with variants exhibiting *(R)*-selectivity clustering toward one side and those with low or opposite stereopreference toward the other (Fig. 6e). All three features contributed comparably and positively to PC1 (loadings ranging from +0.514 to +0.612), consistent with their role in defining binding-site geometry. Thus, the unsupervised PCA results were consistent with the supervised regression models. The *HomoLogic*-derived features produced low-dimensional embeddings that showed good visual alignment with the experimental observables. PC1 remained dominant, revealing clear gradients that correspond to the measured functional variation among orthologs acting on substrate **2b**.

## Discussion

Protein orthology is a powerful organizing principle for enzyme discovery. By inferring and systematically sampling the orthogroup of Ssal-KRED, we obtained functional enzymes across diverse substrates and stereochemical outcomes. In this combined bioinformatic and experimentally driven process, the iterative refinement around initial hits enriched the number of variants with improved catalytic properties while expanding sequence diversity. Beyond the immediate identification of stereoselective KREDs, this study points to a broader conceptual role of ortholog panels: they provide experimentally validated, evolutionarily balanced datasets that are particularly well suited for extracting sequence-function relationships. Our analyses show that even simple structure and binding-site descriptors can capture substantial variance in enzymatic activity and enantioselectivity, suggesting that ortholog-derived datasets support the development of interpretable predictive models. In this sense, ortholog mining can act as a bridge between classical comparative genomics and data-driven enzyme discovery, decoupling the identification of functional homologs from the boundaries of sequenced genomes. Iterative cycles of screening, descriptor extraction, and predictive modeling can guide targeted exploration of the vast pool of unannotated homologous sequences, transforming evolutionary information into scalable search strategies for functional enzymes.

Looking ahead, this discovery framework should be transferable to other enzyme families^72,73^, providing a systematic route to sample natural diversity while generating high-quality datasets required for ML-guided biocatalyst discovery. In this context, two complementary use cases emerge: first, mining for functional homologs of satisfactory hits aiming to surpass their efficiency or process compatibility; and second, broadening substrate scope by identifying more promiscuous orthologs (or example using models such as SPEPP^74^) as conceptually demonstrated with feruloyl esterases^75^. In this context, AI-driven property prediction tools offer complementary capabilities for enzyme selection by forecasting key parameters such as thermostability, solubility, optimal pH, and aggregation propensity^61,76^. An example of this integration is EnzymeMiner 2.0, which pairs large-scale sequence mining with property predictions and filtering to isolate variants tailored to specific user goals^77^. By enabling pre-experimental prioritization of candidates, the models accelerate the overall enzyme mining pipeline while, as demonstrated here, empirical insights from ortholog mining rounds can be used to establish enzyme-family-specific cutoffs for predicted properties. In summary, these perspectives position ortholog mining not only as a tool for zero-shot enzyme mining, but as a complementary technique for modern, iterative, and data-driven enzyme discovery endeavors.

## Methods

### Orthogroup inference

Fungal proteomes were fetched from the Ensembl Genomes website (ftp://ftp.ensemblgenomes.org/pub/fungi/)^42^. The proteomes available in releases 1 to 46 were considered for orthogroup clustering. The protein sequences of *Sporidiobolus salmonicolor* were obtained from NCBI GCA_001373355.1. To reduce the computational burden, we kept only one genome per species and removed any duplicates and multiples. OrthoFinder2^32^ was used using the default setting to cluster the proteome of the 565 fungal species (Supplementary Data 1). Orthogroup clustering was run on 32 CPU (2x Xeon-Gold 6142, 32) and resulted in the identification of 4,181 orthologs and paralogs (Supplementary Data 2).

### Conservation analysis and construction of the phylogenetic tree

To analyze the conservation of cofactor and substrate binding residues, the orthogroups were aligned with MAFFT (v7.450) (applying–*maxiterate 1000* and–*localpair*)^78^. The amino acid distributions at the residues of interest were plotted with ggseqlogo^79^. Similarly, the phylogenetic tree was constructed by first aligning the orthogroup using MAFFT (v7.526) in automatic mode (with the–*auto* and–*leavegappyregion* options). A similarity classification was then performed by comparing each sequence to Ssal-KRED, calculating pairwise identity (considering only non-gap positions), and assigning sequences to similarity groups (High ≥50%, Medium ≥40%, Low ≥30%, Far distant <30%). The aligned sequences were used to infer an approximately maximum-likelihood phylogenetic tree with FastTree^80^ (v2.2.0) in protein mode, employing the LG amino acid substitution model together with gamma-distributed rate variation across sites. The resulting Newick tree file was visualized interactively in iTOL^81^ by uploading the tree file, setting the display mode to Circular, and adding the generated color strip annotation file as a DATASET_COLORSTRIP dataset, which adds colored rings around the tips representing the similarity groups (green for High, yellow-green for Medium, sky blue for Low, and purple for Far distant) together with percentage labels (Supplementary Fig. S1).

### EnzymeMiner-based filtering

EnzymeMiner^23^ is a web server for the in silico identification of enzymes with preserved activity that should be solubly expressible in *E. coli*. Ssal-KRED (UniProt^43^: Q9UUN9) was used as query sequence while keeping the essential residues A108, S133, Y177, and K181 fixed as identified by InterPro^44^. The resulting list of enzymes was then filtered to include only sequences belonging to the Ssal-KRED orthogroup. Subsequently, the sequences were ordered based on their solubility score as predicted by SoluProt^46^ and a cut-off of > 0.75 was applied to bias the selection towards elevated soluble expression.

### Synthesis of genes and expression of enzyme library

All genes were codon optimized for expression in *E. coli* and ordered as gene fragments from Twist Bioscience (USA). Subsequently, the gene fragments were cloned into a pET22b(+) using the available *Nde*I and *Xho*I restriction sites, and the resulting plasmid was transformed into *E. coli* BL21 (DE3) for protein overexpression. The cells harboring the plasmid of interest were cultivated in 0.5 mL LB medium supplemented with 200 µg mL^−1^ ampicillin at 28 °C while shaking at 300 rpm using a Duetz-System (Kühner AG, Switzerland). For KRED expression, 0.5 mL ZYM-5052 medium containing 200 µg mL^−1^ ampicillin was inoculated with 8 µL of the overnight culture. The cultures were incubated at 16 °C for 30 h while shaking at 300 rpm on the Duetz-System. Cells were harvested by centrifugation at 3,320 g at 4 °C for 20 minutes. The supernatant was removed, and the cell pellets were either stored at −20 °C or directly used for the preparation of clarified lysates.

### Preparation of crude cell extracts or clarified lysates

Crude cell extracts were used for reactions with **2a**–**5a**. The bacterial cell pellets were enzymatically lysed using 100 µL lysis buffer (100 mM potassium phosphate buffer pH 7 containing 2 mM MgCl_2_, 1 mg mL^−1^ lysozyme (Sigma Aldrich), 0.75 mg mL^−1^ polymyxin B-sulfate (Carl Roth, Germany), 0.2 mg ml^−1^ DNase (Sigma Aldrich)) for 1 h at 30 °C while shaking at 300 rpm on the Duetz-System. In case of reactions with **1a**, clarified lysates were applied. Cell pellets were resuspended in 200 µL lysis buffer and incubated under the same conditions. Clarified cell lysates were then collected following centrifugation at 3,320 g at 4 °C for 30 minutes.

### Biotransformations of 2a–5a with crude cell extracts

Reactions were carried out in a 96-deepwell plate format. To 100 µL crude cell extracts, 100 µL of 2x biocatalysis mix (100 mM potassium buffer pH 7 containing 2 mM MgCl_2_, 2 mM NADH, 2 mM NADPH, 40 mM glucose, 0.08 mg mL^−1^ glucose dehydrogenase GDH-105 with ~ 50 U mg^−1^, Codexis, USA) were added. The reaction was started by the addition of compounds **2a** – **5a** from a 0.5 M stock solution in DMSO to give a final concentration of 10 mM in the reaction mixture. Biotransformation plates were sealed and incubated at 20 °C for 20 h with shaking at 300 rpm (Duetz-System).

### Reaction monitoring of 1a biotransformation with clarified lysates

Reductase activity was measured in 96-well Greiner microtiter plates using a spectrophotometer (SpectraMAX Plus). Reactions were performed in a total volume of 200 µL containing 178 µL of 100 mM potassium phosphate buffer pH 7 with 2 mM MgCl_2_ and 0.01 mg mL^−1^ NADP^+^ (as sodium salt); 6 µL of clarified cell lysate in 100 M postassium phosphate buffer pH 7 with 2 mM MgCl_2_ to give a final lysate concentration of 3 % (v/v); and 16 µL of a stock solution containing 1.25 mg mL^−1^ **1a** in iPrOH. The assay was run with orbital shaking at 432 rpm and at temperatures oscillating between 28 °C and 32 °C (room temperature plus 5 °C caused by shaking). Substrate depletion at 340 nm was recorded every 5 min for 80 min. Slopes (∆A/min) within the linear range were used for fold-increase over Ssal-KRED calculations. The samples were frozen at −20 °C or immediately processed for selectivity verification via LC-UV.

### Sample processing

Reactions with compound **1a** (UV assay samples, 150 µL) were quenched with 150 µL LC-MS grade methanol. Samples were sealed and then incubated at room temperature for 10 min with shaking at 800 rpm in a thermomixer (Eppendorf). After centrifugation at 4 °C at 3’428 g for 10 min, the samples were analyzed by HPLC-UV. Reactions with compound **2a** were quenched by the addition of 800 µL LC-MS grade methanol:ddH_2_O (62.5:37.5 (v/v)). Plates were sealed and incubated at room temperature for 10 min at 300 rpm (Duetz-system), followed by centrifugation at 4 °C and 3’428 g for 20 min and chiral LC-UV analysis. Reactions of compounds **3a,** **4a** and **5a** were quenched by adding 1 mL GC-MS grade ethyl acetate. Plates were sealed and subjected to incubation and centrifugation following the same protocol indicated above. The organic phases were transferred to a 96-deep well plate, dried over MgSO_4_ anhydrous, centrifuged and sealed again for chiral GC-FID analysis.

### Diastereoselectivity verification of 1a biotransformations

Samples were analyzed by HPLC-UV at 260 nm on an Agilent 1290 HPLC system using a Kinetex XB-C18 column (50 mm×4.6 mm, 2.6 µm) or an Agilent InfinityLab Poroshell 120 Eclipse EC-C18 column (50 mm × 3.0 mm × 2.7 µm). The column was heated to 50 °C, using water and methanol as mobile phases A and B, respectively. The flow rate was 0.8 mL min^−1^. In case of the Kinetex column, the following gradient was used: 0–0.1 min, B = 50%; 0.1–4 min, B = 50–58%; 4–4.1 min, B = 58–95%; 4.1 –4.6 min, B = 95%; 4.6–4.7 min, B = 95 – 50%; 4.7–5 min, B = 50%, 5–5.1 min, B = 50–95%; 5.1–5.4 min, B = 95%; 5.4–5.5 min, B = 95–50%; and 5.5–5.9 min, B = 50%. In case of the Poroshell column, the following gradient was used: 0–0.1 min, B = 40%; 0.1–6 min, B = 40–52.7%; 6–6.2 min, B = 52.7–95%; 6.2–7.2 min, B = 95%; 7.2–8 min, B = 95–40%; 8–9 min, B = 40%. Standards of substrate **1a** as well as products *trans-(R,R)-***1b** and *cis-(R,S)-***1b** were treated as the samples prior to analysis. Diastereomeric excess (*de*) values were estimated using the relative peak areas corresponding to the retention times of *trans-(R,R)-***1b** and *cis-(R,S)-***1b** (*de* specified as >99% when the opposite enantiomer was not observed).

### Chiral HPLC-UV or GC-FID analysis of 2b–5b

Reaction analysis of biotransformations with compound 2a was performed by injecting 4 μL of sample in an Agilent 1290 HPLC-DAD system (Agilent, USA) equipped with a Chiralpak IE-3 column (150 mm × 4.6 mm, 3.0 μm; Daicel Corporation, Japan). Mobile phase A consisted of water with 5% acetonitrile and 0.2% formic acid, while Mobile phase B contained acetonitrile with 0.2% formic acid. The flow rate was 1.2 mL min^−1^ and the the following gradient was used: 10% B for 6 min, 10–55% in 1 min, 55–85% B in 2.5 min. Substrate **2a** was detected at 254 nm with a retention time of 8.97 min, while *(R)-***2b** and *(S)-***2b** were detected at 200 nm with retention times of 7.22 min and 7.57 min, respectively.

The analyses of reactions with compounds **3a,** **4a** and **5a** were performed by injecting 1 µL of sample in an Agilent GC-FID/MS instrument equipped with a MEGA-DEX ASX 2-92 (0.25 mm × 0.25 µm × 30 m; MEGA S.r.1) and using a gas flow of 1.5 mL min^−1^ (helium). The injector was set to 250 °C, the FID to 300 °C, and the MSD to 250 °C. For reactions toward **3a**, the oven was set to 120 °C for 1 min, followed by a ramp to 169 °C at a rate of 2 °C min^−1^, a subsequent ramp to 200 °C at 40 °C min^−1^, and a hold time of 3 min. Retention times of **3a,** *(R)*-**3b** and *(S)*-**3b** were 22.37 min, 24.23 min, and 24.60 min, respectively. For biotransformations containing **4a**, the oven was set to 120 °C for 1 min, followed by a ramp to 138 °C at a rate of 2 °C min^−1^, a subsequent ramp to 145 °C at 0.5 °C min^−1^, then to 200 °C at 40 °C min^−1^, and a hold time of 3 min. Retention times of **4a,** *(R)*-**4b** and *(S)*-**4b** were 18.59 min, 23.17 min, and 23.49, respectively. For reactions containing compound **5a**, the oven was set to 55 °C and a ramp to 120 °C at 1.5 °C min^−1^, followed by a ramp to 200 °C at 40 °C min^−1^, and a hold time of 3 min. Retention times of **5a,** *(R)*-**5b** and *(S)*-**5b** were 30.01 min, 43.83 min, and 44.04 min, respectively. Product quantification, conversion and enantioselectivity values were calculated using calibration curves of each *(R)*- or *(S)*-product standard and the initial substrate concentration (*ee* values were specified as >99% when the opposite enantiomer was not observed).

### Melting temperature determination

Melting temperatures of all wildtypes were determined by differential scanning fluorimetry using a Prometheus Panta (NanoTemper Technologies). Enzymes were expressed in a 96-deep-well plate format as described above. Cell pellets were lysed by three freeze-thaw cycles, alternating between 4 °C (30 min) and −80 °C (30 min)^82^. Clarified lysates were prepared by resuspending pellets in 100 µL of 10 mM potassium phosphate buffer (pH 7.0), agitating for 30 min at 20 °C and 300 rpm on a Duetz system, and centrifuging at 3’320 g for 20 min at 4 °C. Supernatants were transferred to capillaries and thermal unfolding was performed from 20 to 95 °C at a ramp rate of 1 °C min⁻¹. Melting temperatures were extracted from the resulting unfolding profiles using PR.Panta Analysis software (v1.6.3).

### Visualization of ortholog screening data

Multiple sequence alignments of the selected protein sequences were generated using Clustal Omega^83^ (v1.2.3) with default parameters and the BLOSUM62 distance matrix. Hierarchical clustering was performed on the resulting distance matrix using the average (UPGMA) linkage method implemented in scipy^84^ (v1.16.3), and the resulting dendrogram determined the phylogenetic ordering of sequences for visualization. Heatmaps were created with seaborn^85^ (v0.13.2) and matplotlib^86^ (v3.10.8): the first column displayed substrate **1a** screening results (column “1b”, green colormap, vmax = 3.0), while subsequent paired columns showed substrate **2a**–**5a** results for both enantiomeric products (blue-white colormap, vmax = 7.0). All heatmaps used white grid lines for cell separation, black-bordered spines, and were arranged side-by-side with a right-oriented dendrogram to reflect sequence similarity-based ordering.

### HomoLogic: a fully automated computational pipeline for enzyme homology quantification

To enable systematic comparison of homologous enzymes and to generate quantitative descriptors for data-driven enzyme discovery, we developed HomoLogic, a modular Python workflow that integrates sequence, structure and binding-site analyses into a unified computational pipeline (https://github.com/Buller-Lab/HomoLogic). The workflow operates on two input categories: a query enzyme and a haystack of putatively homologous sequences (Fig. 5). The query enzyme is defined by a reference amino-acid sequence, a reference protein structure, coordinates of the bound ligand(s), and optional ligand representations encoded as SMILES strings. The haystack consists of a set of candidate sequences belonging to the same enzyme family or structural fold. Such sequence sets can originate from sequence homology searches (e.g., via BLAST^19^ or MMseqs2^20^ against databases such as UniProt^43^, Swiss-Prot^87^, or NCBI RefSeq^88^), structure- or embedding-based searches (e.g., Foldseek^21^ or pLM-BLAST^89^), position-filtered enzyme mining tools such as EnzymeMiner^23^, orthology-based pipelines including OrthoFinder^33^ and SHOOT^34^, or curated proprietary enzyme libraries.

The HomoLogic workflow first quantifies sequence similarity between the query enzyme and all haystack sequences using MMseqs2 (easy-search mode). The resulting pairwise alignment statistics, including sequence identity and alignment length, are stored in a table (sequence_homology.csv). Structural models for all haystack sequences are subsequently generated using Boltz-2^68,69^, optionally co-folding one or more ligands provided as SMILES strings to preserve the spatial context of the active site. Input FASTA files containing protein sequences and ligand definitions are generated automatically, enabling ligand-aware structure prediction. Resulting models are converted into protein-only structures for downstream analysis. Alternatively, structures may be obtained from external databases such as RCSB Protein Data Bank^90^ and AlphaFold Protein Structure Database^91^, or generated using faster prediction methods such as ESMFold^62^, provided that protein-only PDB files are available for each haystack sequence with PDB filenames corresponding to the header in the haystack FASTA file.

Structural similarity between the reference structure and predicted homolog models is quantified using TM-align^54^, yielding TM-scores and RMSD values (structure_homology.csv). All structures are subsequently superposed onto the reference structure using Cα-atom alignment implemented in PyMOL^70^, ensuring a common coordinate frame. Binding sites are defined as residues located within 6 Å of the reference ligand coordinates using neighbor-search routines implemented in Biopython^92^ (v1.84). Equivalent binding-site regions are extracted from both reference and homolog structures and subsequently refined through local structural superposition to quantify binding-site RMSD and the number of structurally aligned Cα atoms. To capture sequence variation within structurally equivalent active-site regions, HomoLogic constructs binding-site “metasequences.” These sequences represent spatially corresponding residues identified through Cα proximity within a tolerated positional deviation of 1 Å between reference and homolog binding sites. Global pairwise alignment of metasequences is performed using the implementation provided in scikit-bio^93^ (v0.7.0) with substitution matrices from Biopython (default: the BLOSUM62 substitution matrix). Alignment scores are normalized by the self-alignment score of the reference binding-site metasequence to generate a binding-site similarity metric. In addition, structural confidence of predicted models is evaluated using per-residue pLDDT values from Boltz-2 predictions, which are summarized for both full proteins and binding-site residues (bindingsite_similarity.csv).

Finally, the geometric and physicochemical properties of enzyme binding pockets are characterized using pyKVFinder^71^. Cavities are detected using customizable outer probe radius of 4 Å and a default minimum volume threshold of 100 Å³. For each binding site, descriptors including cavity volume, surface area, depth, hydropathy and residue class frequencies are calculated. To compare pocket geometries across homologs, cavity point-clouds are aligned to the reference cavity using iterative closest-point registration implemented in Open3D^94^, an algorithm widely used for point-cloud alignment. All sequence-, structure- and cavity-derived descriptors are integrated into a final output table (bindingsite_cavity_homology.csv), providing a unified feature set for comparative enzyme analysis and machine-learning-guided enzyme discovery.

### MMseqs2-based homology search

Our previously published script (get_homologs.py^95,96^) was utilized to identify all Ssal-KRED sequence homologs from Swiss-Prot database. The code performs a sensitive homology search with MMseqs2 easy-search against the respective pre-built MMseqs2 database, using a minimum sequence identity of 25% and retrieving extensive hits (up to very large numbers of sequences per query) while outputting key fields including query, target, percent identity, taxonomy, and sequence.

### HomoLogic analysis

HomoLogic analysis was performed on both SwissProt-derived Ssal-KRED homologs and solubly expressed orthologs (rounds 1 and 2). Using Ssal-KRED-NADPH-**1a** complex as the reference structure^16^, all HomoLogic functions were applied, and the resulting feature sets were used for statistical analysis. To assess reference structure–dependent effects on derived features, the soluble ortholog dataset was additionally analyzed using Ort-RDM-1_3 as reference. For this purpose, Boltz-2 (v2.2.0) was employed to co-fold Ort-RDM-1_3 with NADPH and **2a**, and the resulting protein–ligand complex served as the reference structure for HomoLogic analysis, with all functions applied and feature sets subjected to statistical evaluation.

### Significance testing Swiss-Prot homologs vs. tested orthologs

Feature sets for significance analysis, including sequence identity to the closest homolog and TM-score-based structural similarity, were generated using the HomoLogic pipeline. Two datasets were compared: orthologs (filtered to sequence identity ≤ 0.3 to focus on far-distant homologs) and Swiss-Prot homologs (*vide supra*; equally filtered), with completely missing columns removed from both data frames. Independent two-sample t-tests (Welch’s version, assuming unequal variances) were applied to assess differences in mean values between the ortholog and Swiss-Prot groups for each common numeric feature, with *p*-values reported and significance declared at α = 0.05. Descriptive statistics (count, mean, median, standard deviation, variance, min/max, skewness, and kurtosis) were computed for each feature and group using the scipy.stats module. All data processing, statistical tests, and output generation were performed in Python (v3.14.2) with pandas (v3.0.0) for data manipulation, numpy (v2.4.2) for numerical operations, and scipy (v1.17.0) for statistics.

### Correlation analysis of HomoLogic-derived features

Pairwise Pearson correlation coefficients were calculated between 16 selected HomoLogic-derived descriptors and experimentally determined product formation (and enantioselectivity) for substrates 1a – 5a, using only complete pairwise observations. The descriptors comprised ten binding pocket properties (number of binding-site residues, cavity volume, surface area, average depth, average hydropathy, amino-acid type frequencies in the cavity, and cavity point count), three global structural similarity metrics (sequence identity, RMSD, TM-score), and three binding-site-specific similarity measures (binding-site RMSD and two HomoLogic*-*specific similarity scores). Statistical significance was assessed via two-tailed *p*-values, with results visualized in a diverging heatmap (purple-white-green scale, r = −1 to +1) that annotated each coefficient (two decimal places) together with significance level (* *p* < 0.05, ** *p* < 0.01, *** *p* < 0.001) and displayed the number of observations (N) for each experimental variable. All computations were performed in Python (v3.14.2) using pandas (v2.3.3), stats.pearsonr from scipy (v1.17.0), seaborn (v0.13.2) and matplotlib (v3.10.8).

### LASSO-regularized linear regression

LASSO-regularized^66^ multiple linear regression was employed to identify the most influential features driving stereochemical outcomes for **2b**, specifically the relative formation of the **2b** *(R)* and the signed enantiomeric excess (*ee* **2b** (%), with positive values indicating *(R)-*excess and negative values indicating *(S)-*excess). The candidate feature set comprised the same 16 HomoLogic-derived descriptors used in the correlation analysis (*vide supra*), covering global sequence and structural similarity, binding-site geometry and similarity, cavity physicochemical properties, and amino-acid type frequencies within the substrate-binding cavity. Features were standardized prior to analysis, and LASSO regression with 5-fold cross-validation was applied separately to each target to perform automatic feature selection by shrinking less relevant coefficients toward zero. The three descriptors with the largest absolute coefficients were selected for each outcome and interpreted as the primary structural drivers of stereoselectivity. Their relative contributions were visualized using pi charts of normalized absolute LASSO coefficients, while ordinary least-squares linear models fitted on these top three features provided supporting performance statistics, including full-dataset R², adjusted R², RMSE, and Leave-one-out cross-validation (LOO-CV) metrics (Q², RMSE, MAE, Pearson correlation coefficient, and overfitting indicator Δ(R²-Q²)). All analyses, feature ranking, model fitting, and visualization were implemented in Python (v3.14.2) using scikit-learn (v1.8.0), scipy (v1.17.0), pandas (v3.0.0), numpy (v2.4.2), and matplotlib (v3.10.8).

### Assessment of generalization capacity

To assess the generalization capacity of the models toward more distant homologs, we performed sequence-based redundancy reduction using MMseqs2 (easy-cluster) at sequence identity thresholds of 90 %, 80 %, 70 %, 60 %, and 50 %. Thresholds below 50 % were not investigated, as the number of remaining datapoints became too low for reliable LOO-CV. For each threshold, only the representative centroid sequences were retained, and the previously selected three-feature linear regression models were retrained and evaluated by LOO-CV on the reduced datasets. This procedure was applied independently to both targets (**2b** *(R)* formation and *ee* **2b** (%)) and implemented in Python (v3.14.2) using scikit-learn (v1.8.0), scipy (v1.17.0), pandas (v3.0.0), numpy (v2.4.2), and matplotlib (v3.10.8).

### Principal component analysis

Principal component analysis (PCA) was performed on the top three HomoLogic-derived structural descriptors previously identified by LASSO regression as the most influential for stereochemical outcomes of **2b** (*vide supra*), in order to visualize the distribution of enzyme variants in the reduced feature space and explore potential clustering or gradients related to catalytic performance. The same three features were standardized (zero mean, unit variance) after median imputation of any missing values, and PCA was computed to extract the first two principal components explaining the majority of variance in this low-dimensional subspace. Results were visualized in a 2D scatter plot where points represent individual enzyme variants, colored continuously by the target property of interest (either **2b** *(R)* formation or signed enantiomeric excess of **2b**) using a diverging colormap; samples with missing target values were plotted in faded gray. Explained variance ratios for PC1 and PC2, as well as their cumulative proportion, were reported, together with feature loadings to identify which descriptors contributed most strongly to each principal component (sorted by absolute loading magnitude). All preprocessing, PCA computation, and visualization were implemented in Python (v3.14.2) using scikit-learn (v1.8.0), pandas (v3.0.0), numpy (v2.4.2), and matplotlib (v3.10.8).

### Reporting summary

Further information on research design is available in the Nature Portfolio Reporting Summary linked to this article.

## Supplementary information

Supplementary Information

Description of Additional Supplementary Files

Supplementary Data 1

Supplementary Data 2

Supplementary Data 3

Supplementary Data 4

Reporting Summary

Transparent Peer Review file

## Source data

Source Data

Source Data for Supplementary Fig. 3

## Data Availability

The experimental data generated in this study, including biocatalytic data (GC/LC peak data, conversions, enantiomeric and diastereomeric excesses, and calibration curves) and enzyme melting temperatures, have been deposited on Zenodo (https://doi.org/10.5281/zenodo.19574983). Third-party sequence data utilized for ortholog and homolog searches were obtained from the publicly available Ensembl Genomes, SwissProt and UniProt databases; the resulting extracted sequences and search outputs are provided within the accompanying GitHub repository (https://github.com/Buller-Lab/Ssal-KRED_orthologs). This repository also contains the source data and input files required for generating the plots and performing post-analysis. For long-term accessibility, the repository has been versioned and deposited on Zenodo (https://doi.org/10.5281/zenodo.20756197. A list with all genome assembly identifiers utilized for orthogroup definition (“Supplementary Data 1”), the fasta file of the full Ssal-KRED orthogroup (“Supplementary Data 2”), and an overview of all tested orthologs with experimental results (“Supplementary Data 3”) are available as Supplementary Data. Experimental raw data and methods are provided with this paper, the Supplementary Information or are available from the authors upon request. Source data are provided with this paper.

## Code availability

The Python code used for phylogenetic analysis, homolog identification, predictive modeling (HomoLogic), and data visualization, along with all corresponding input/output files and Conda environment specifications for reproducibility have been deposited on Zenodo (https://doi.org/10.5281/zenodo.20756197). The repository is additionally maintained and publicly accessible on GitHub (https://github.com/Buller-Lab/Ssal-KRED_orthologs).
