# Supplementary material for: Iterative and data-driven ortholog mining enables reliable discovery of stereoselective ketoreductases
Source: Nat Commun. 2026 Sep 15;17:10854. doi: 10.1038/s41467-026-77715-6 (PMC13672348; doi:10.1038/s41467-026-77715-6)
Supplement: Supplementary file 1 [file 41467_2026_77715_MOESM1_ESM.pdf]

# Supplementary Information

# Iterative and data-driven ortholog mining enables reliable discovery of stereoselective ketoreductases

Peter Stockinger,[1,2,3†] Michael Niklaus,[2†] Kevin M. Yar,[2,4] Nicolas Imstepf,[1,2,5] Ivana Pastierikova,[2,6] Jasmin Küng,[2,7] Steven Hanlon,[8] Hans Iding,[8] Sumire Honda Malca,[2] and Rebecca Buller[1,2]*

[1] Department of Chemistry, Biochemistry and Pharmaceutical Sciences, University of Bern, 3012 Bern, Switzerland

[2] Institute of Chemistry and Biotechnology, Zurich University of Applied Sciences, 8820 Wädenswil, Switzerland

[3] Present address: Process Chemistry and Catalysis, F. Hoffmann-La Roche Ltd., Grenzacherstrasse 124, 4070 Basel, Switzerland

[4] Present address: Omics-OS, Inc., 995 Market Street, 94103 San Francisco, USA

[5] Department of Biotechnology, Delft University of Technology, 2629 HZ Delft, The Netherlands

[6] Present address: Lonza, Drug Product Services, Hochbergerstrasse 60, 4057 Basel, Switzerland

[7] Present address: RGCC International, Baarerstrasse 95, 6300 Zug, Switzerland

[8] Process Chemistry and Catalysis, F. Hoffmann-La Roche Ltd., Grenzacherstrasse 124, 4070 Basel, Switzerland

*Corresponding author: rebecca.buller@unibe.ch

†These authors contributed equally

**Phylogenetic tree and conservation analysis**

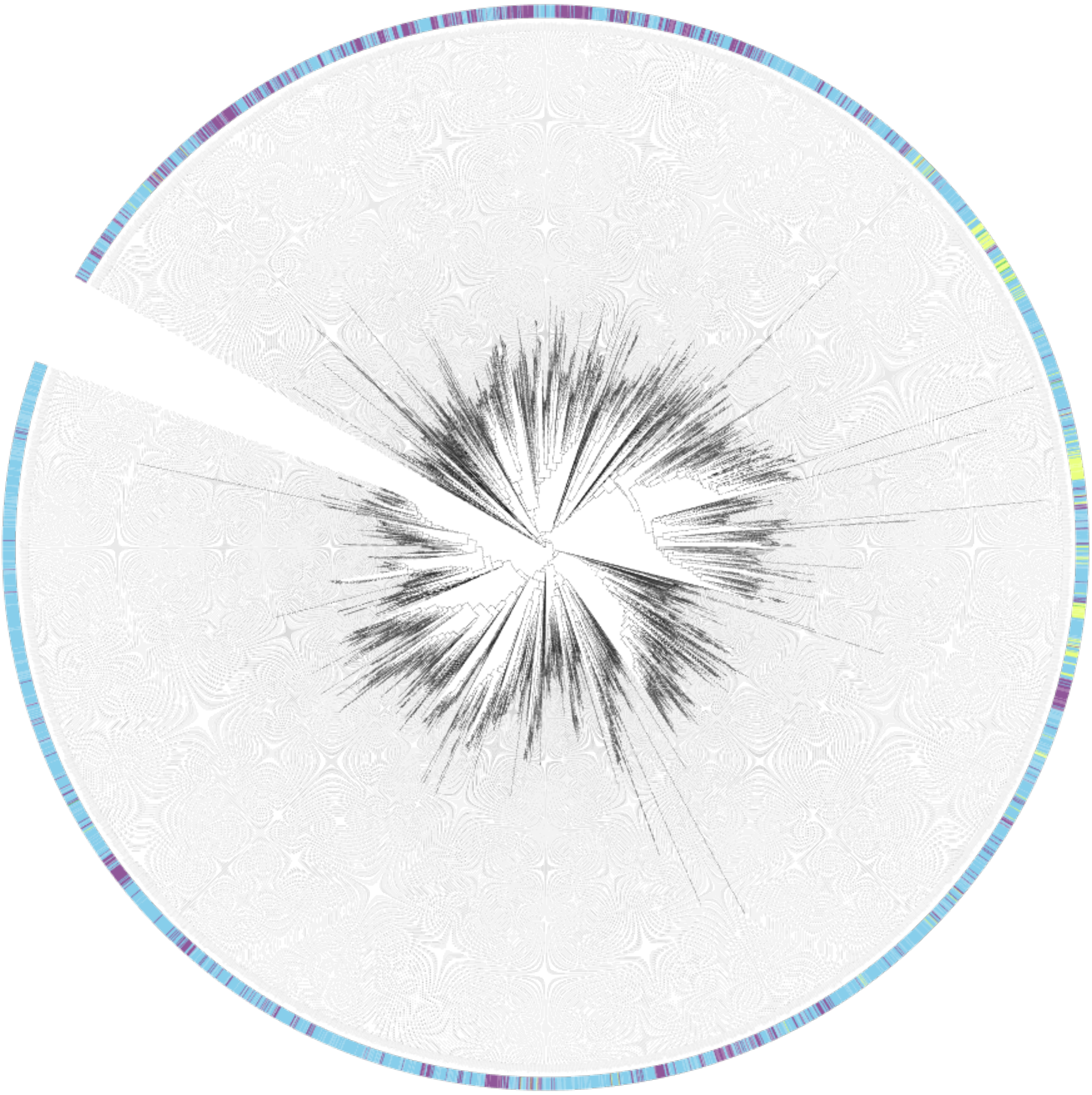

**Figure S1: Neighbor-joining phylogenetic tree of the Ssal-KRED orthogroup** (4181 sequences). Color strips indicate percent sequence identity to the reference sequence Ssal-KRED (green: ≥50%, light yellow: 40–49%, light blue: 30–39%, purple: <30%). The tree was constructed using FastTree[1] from a MAFFT[2] alignment. Tree visualization was performed in iTOL.[3] Code and input sequences and output files for interactive visualization of the phylogenetic tree in iTOL are available on our GitHub Repository (https://github.com/Buller-Lab/Ssal-KRED_orthologs/tree/main/Full_orthogroup_tree_of_life).

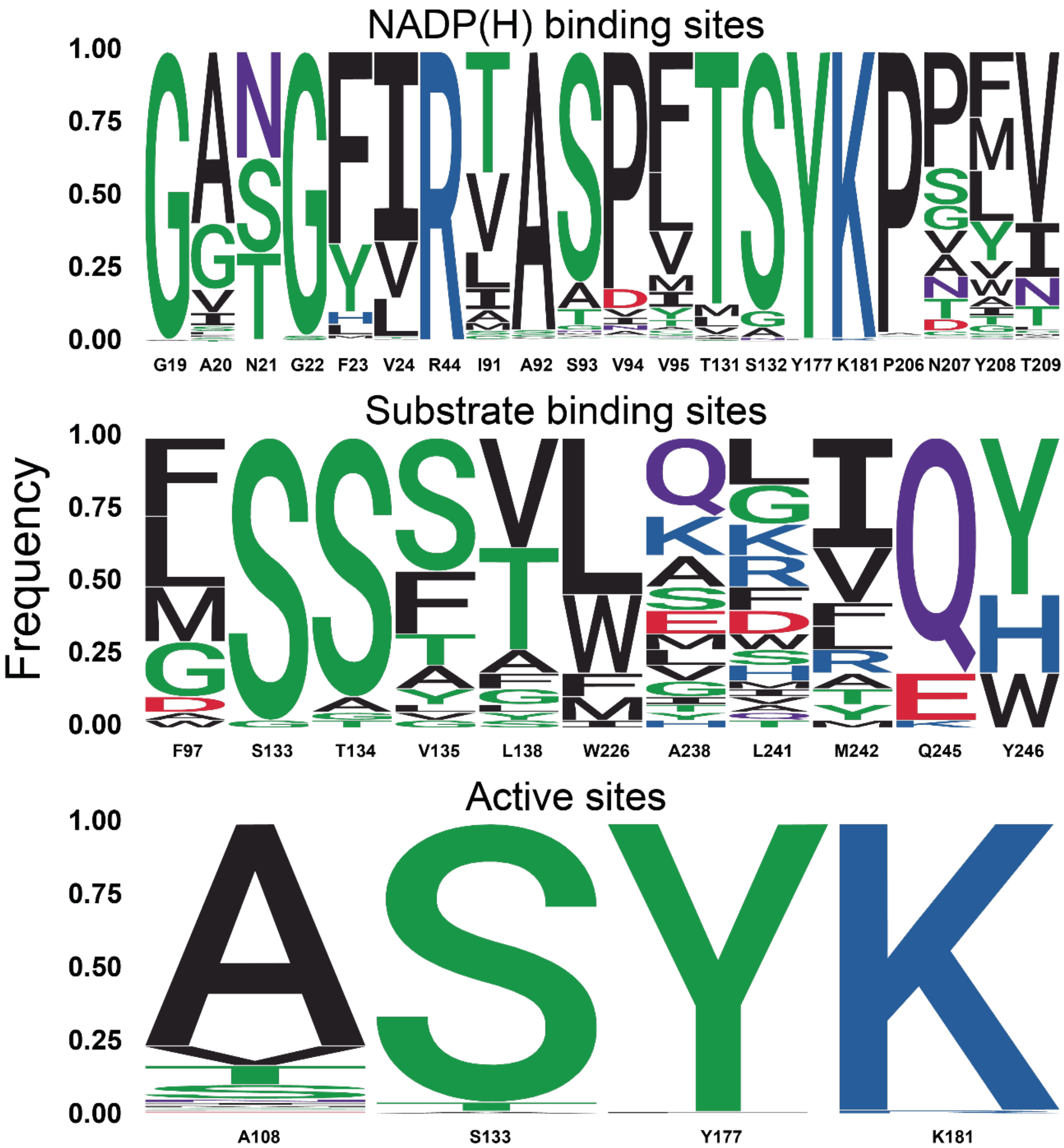


**Figure S2: Natural diversity in functional sites.** Sequence logo (generated using ggseqlogo[4]) representing the occurrence of residues forming NADP(H) binding site, substrate binding site and active site of the enzymes in the Ssal-KRED orthogroup. The numbering of the positions is based on Ssal-KRED from *Sporidiobolus salmonicolor* (UniProt[5] and InterPro[6] accession Q9UUN9).

## SDS-PAGE analysis of Ssal-KRED orthologs

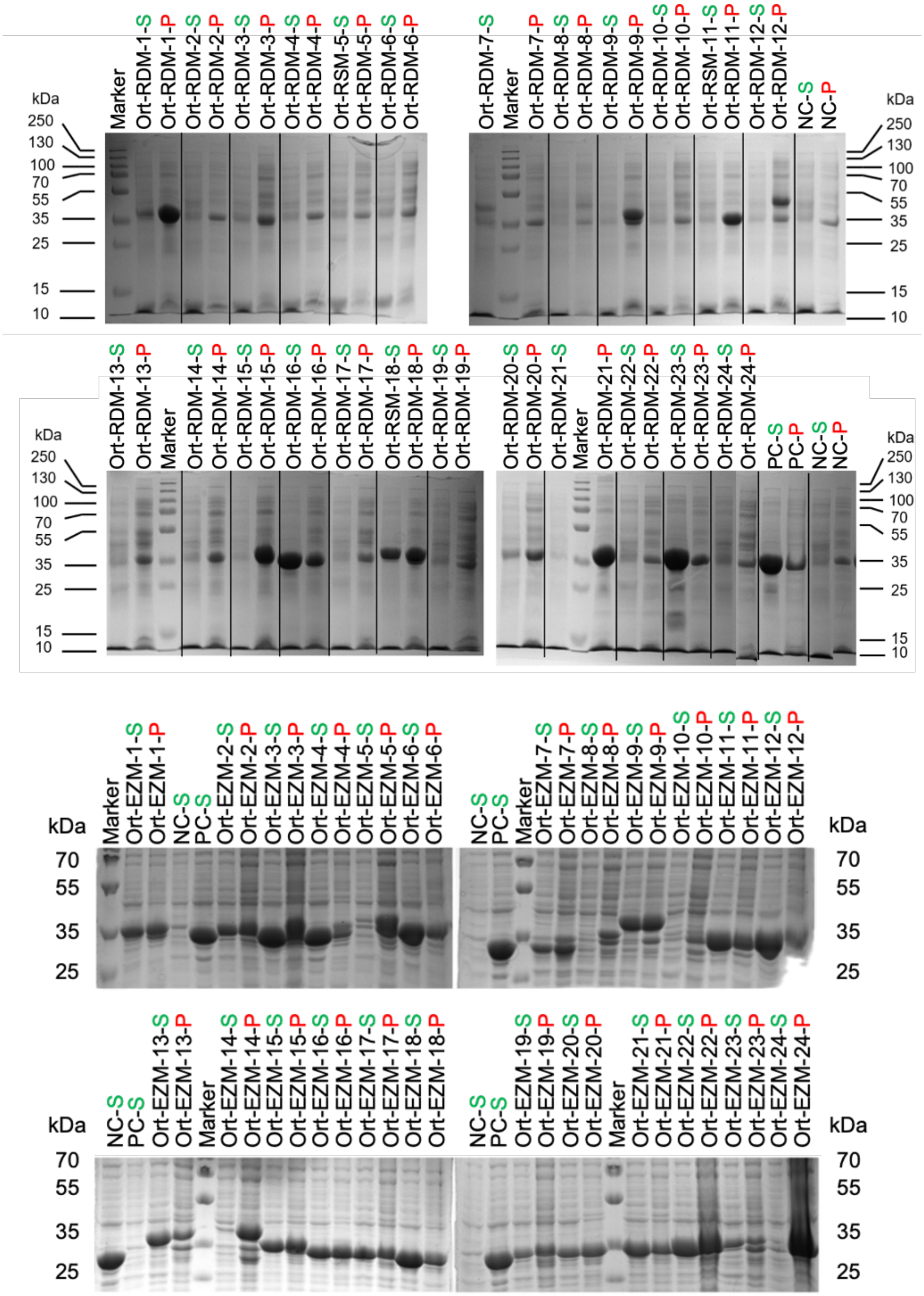


**Figure S3: Qualitative evaluation of protein expression levels.** Soluble (S) and insoluble (P) fractions of the *E. coli* lysates were utilized to analyze the success of the recombinant expression via SDS-PAGE. Negative controls (NC = empty vector) and positive controls (PC = Ssal-KRED) were run in multiple biological replicates. Molecular weight (MW) in kDa was marked with PageRuler with prestrained Protein Ladder (Themo Fisher Scientific, Product No. 2669) are shown on the left and right of the gels. Band positions and marker range may differ between gels due to variations in running time. Uncropped and unedited gel images are available in the Source Data.

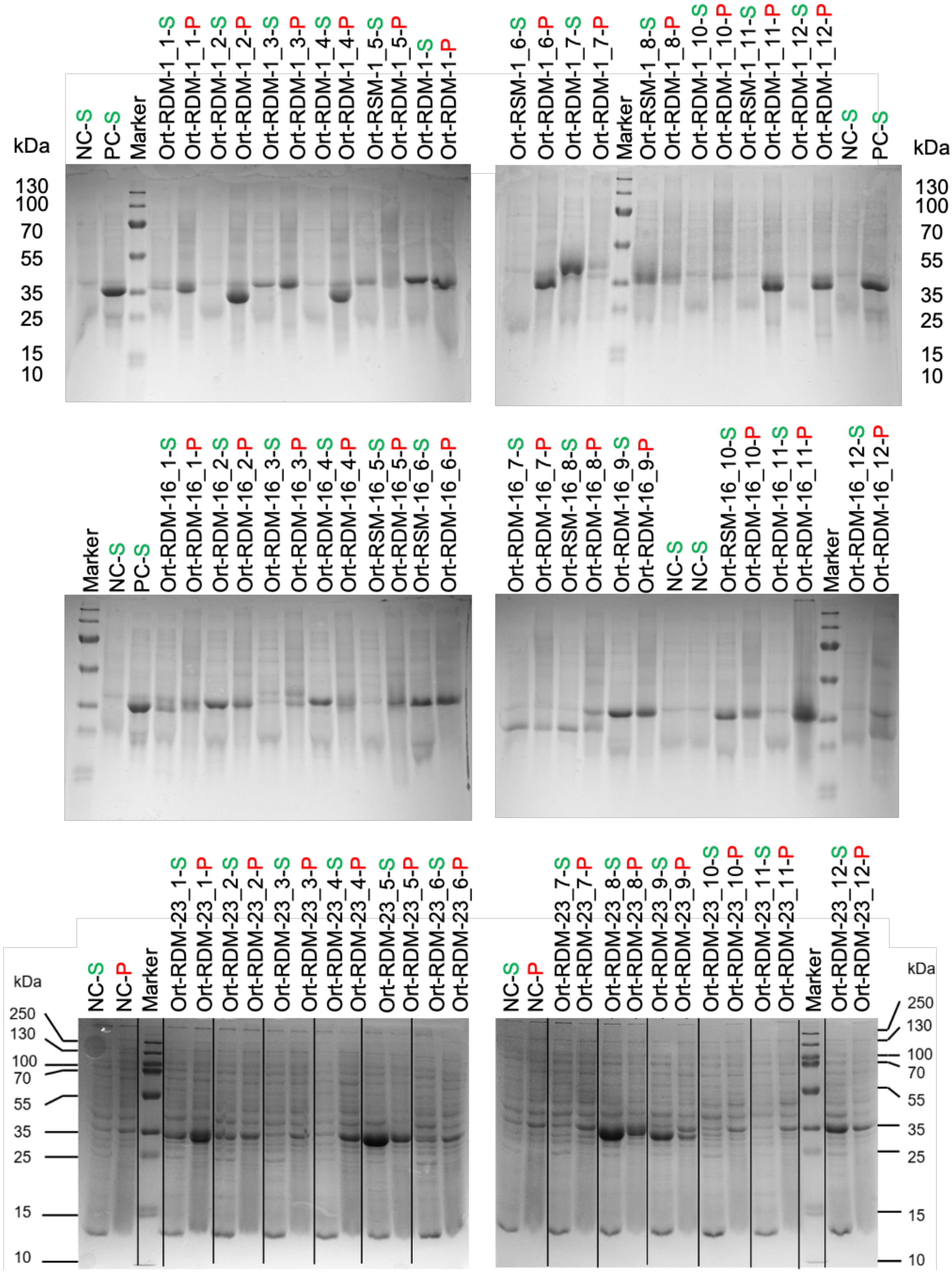


**Figure S3:** SDS-PAGE analysis of Ssal-KRED orthologs (continued).

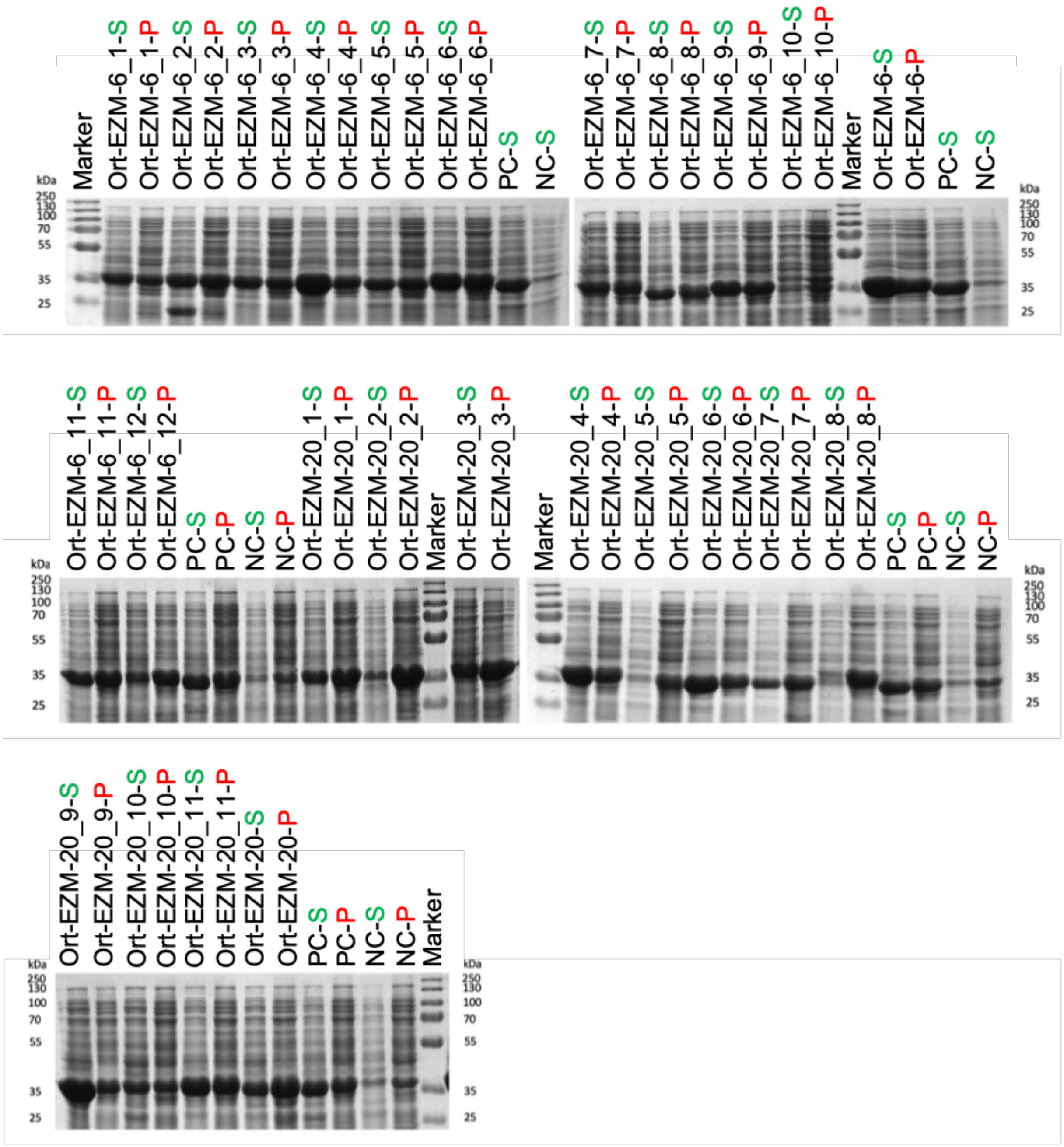


**Figure S3:** SDS-PAGE analysis of Ssal-KRED orthologs (continued).

## Definition of a Solubility Score Cutoff for Ssal-KRED Orthologs

Intrigued by the enrichment of variants that can be solubly produced in *E. coli* utilizing EnzymeMiner-filtering, we analyzed the predictive performance of the underlying solubility predictor SoluProt[7], alongside the widely applied algorithm Protein-Sol[8] and variations of the more recently developed NetSolP[9]. Additionally, filtering-capability of isoelectric point (pI), global sequence identity (SeqID), and structural similarity (TM-score) to Ssal-KRED were investigated to provide a baseline (Figure S3a; Table S1). Solubility scores were calculated by submitting the protein FASTA sequences to the respective web servers. The isoelectric point (pI) was directly extracted from the Protein-sol server output. Sequence identity and structural similarity (TM-score) to the closest homologs were computed with the HomoLogic pipeline utilizing Ssal-KRED as reference. Performance evaluation was carried out in Python (v3.14.2) using the following libraries: pandas (v2.3.3) for data handling, numpy (v2.4.1) for numerical operations, matplotlib[10] (v3.10.8) for plotting, and scikit-learn (v1.8.0) for computation of ROC curves, AUC, Matthews correlation coefficient (MCC), and confusion matrices.[11] ROC-AUC analysis ranked proteins by their continuous predictor scores (higher values indicating higher predicted solubility), plotting true positive rate versus false positive rate across all possible thresholds and computing AUC via the trapezoidal rule; eleven predefined cutoffs (0.30 to 0.80 in 0.05 increments) were then applied to binarize predictions and derive MCC, sensitivity, specificity, and confusion matrices for each predictor-cutoff pair.

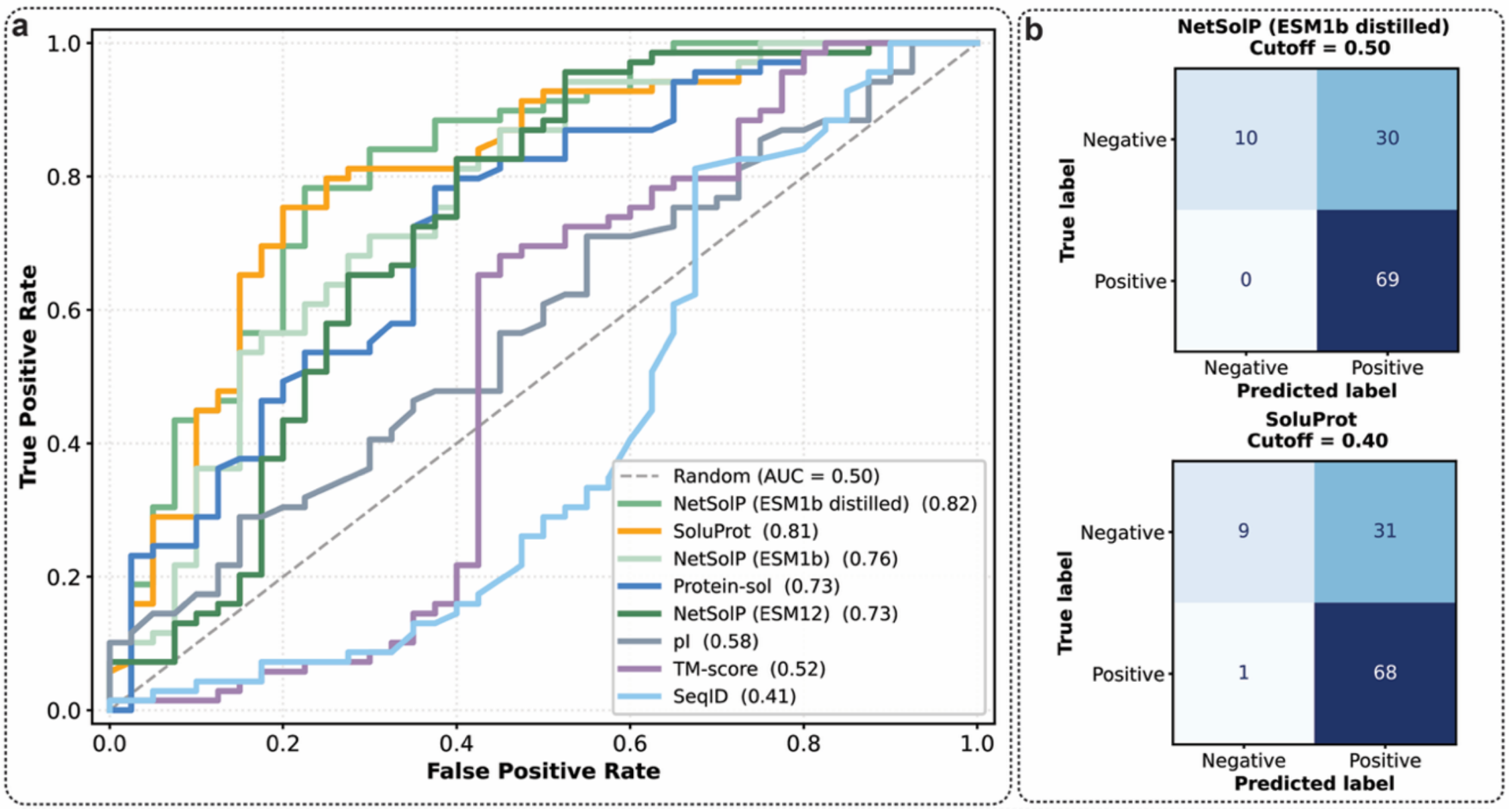


**Figure S4: Retrospective benchmarking of protein solubility predictors. (a)** To quantify the performance of different solubility predictors in discriminating between solubly expressed (n=68) and insoluble enzymes (n=40), their area under the receiver operating characteristic curve (ROC-AUC) was calculated. **(b)** Confusion matrices are shown for the threshold minimizing the amount of false negatives. Input data such as the underlying data, utilized code, and output files can be found on our GitHub Repository (https://github.com/Buller-Lab/Ssal-KRED_orthologs/tree/main/Benchmark_solubility_predictors).

Among the tested predictors, NetSolP (ESM1b distilled) and SoluProt clearly emerged as the strongest performers overall (AUC = 0.82 and 0.81, respectively; Supplementary Figure S3a). To minimize the loss of true positives (i.e., avoid filtering out soluble variants) while still removing as many negatives as possible, we systematically determined optimal operating points. Applying a threshold of 0.5 for NetSolP (ESM1b distilled) achieved perfect sensitivity (FN = 0, Sensitivity = 1.00) while correctly filtering 10 out of 40 negatives (Specificity = 0.25; Table S1; Figure S3b). For SoluProt a slightly relaxed threshold of 0.40 achieved a similar sensitivity (FN = 1, Sensitivity = 0.986; Figure S3b) and filters 9 out of 40 negatives (Specificity = 0.225; Table S1).

**Table S1: Threshold definition to optimize predictivity of different protein solubility predictors.** Input data, and utilized code can be found on our GitHub Repository (https://github.com/Buller-Lab/Ssal-KRED_orthologs/tree/main/Benchmark_solubility_predictors).

| Predictor | Cutoff | MCC | TP | TN | FP | FN | Total | Sensitivity | Specificity |
|---|---|---|---|---|---|---|---|---|---|
| NetSolP (ESM12) | 0.3 | 0 | 69 | 0 | 40 | 0 | 109 | 1 | 0 |
| NetSolP (ESM12) | 0.35 | 0.1264 | 69 | 1 | 39 | 0 | 109 | 1 | 0.025 |
| NetSolP (ESM12) | 0.4 | 0.221 | 69 | 3 | 37 | 0 | 109 | 1 | 0.075 |
| NetSolP (ESM12) | 0.45 | 0.2335 | 68 | 5 | 35 | 1 | 109 | 0.9855 | 0.125 |
| NetSolP (ESM12) | 0.5 | 0.4682 | 64 | 19 | 21 | 5 | 109 | 0.9275 | 0.475 |
| NetSolP (ESM12) | 0.55 | 0.3218 | 42 | 29 | 11 | 27 | 109 | 0.6087 | 0.725 |
| NetSolP (ESM12) | 0.6 | 0.0854 | 9 | 37 | 3 | 60 | 109 | 0.1304 | 0.925 |
| NetSolP (ESM12) | 0.65 | 0.0733 | 1 | 40 | 0 | 68 | 109 | 0.0145 | 1 |
| NetSolP (ESM12) | 0.7 | | 0 | 40 | 0 | 69 | 109 | 0 | 1 |
| NetSolP (ESM12) | 0.75 | | 0 | 40 | 0 | 69 | 109 | 0 | 1 |
| NetSolP (ESM12) | 0.8 | | 0 | 40 | 0 | 69 | 109 | 0 | 1 |
| NetSolP (ESM1b distilled) | 0.3 | 0 | 69 | 0 | 40 | 0 | 109 | 1 | 0 |
| NetSolP (ESM1b distilled) | 0.35 | 0 | 69 | 0 | 40 | 0 | 109 | 1 | 0 |
| NetSolP (ESM1b distilled) | 0.4 | 0 | 69 | 0 | 40 | 0 | 109 | 1 | 0 |
| NetSolP (ESM1b distilled) | 0.45 | 0.221 | 69 | 3 | 37 | 0 | 109 | 1 | 0.075 |
| NetSolP (ESM1b distilled) | 0.5 | 0.4174 | 69 | 10 | 30 | 0 | 109 | 1 | 0.25 |
| NetSolP (ESM1b distilled) | 0.55 | 0.446 | 64 | 18 | 22 | 5 | 109 | 0.9275 | 0.45 |
| NetSolP (ESM1b distilled) | 0.6 | 0.5214 | 54 | 30 | 10 | 15 | 109 | 0.7826 | 0.75 |
| NetSolP (ESM1b distilled) | 0.65 | 0.2361 | 16 | 38 | 2 | 53 | 109 | 0.2319 | 0.95 |
| NetSolP (ESM1b distilled) | 0.7 | -0.0377 | 1 | 39 | 1 | 68 | 109 | 0.0145 | 0.975 |
| NetSolP (ESM1b distilled) | 0.75 | | 0 | 40 | 0 | 69 | 109 | 0 | 1 |
| NetSolP (ESM1b distilled) | 0.8 | | 0 | 40 | 0 | 69 | 109 | 0 | 1 |
| NetSolP (ESM1b) | 0.3 | 0 | 69 | 0 | 40 | 0 | 109 | 1 | 0 |
| NetSolP (ESM1b) | 0.35 | 0 | 69 | 0 | 40 | 0 | 109 | 1 | 0 |
| NetSolP (ESM1b) | 0.4 | 0.1796 | 69 | 2 | 38 | 0 | 109 | 1 | 0.05 |
| NetSolP (ESM1b) | 0.45 | 0.288 | 69 | 5 | 35 | 0 | 109 | 1 | 0.125 |
| NetSolP (ESM1b) | 0.5 | 0.3696 | 69 | 8 | 32 | 0 | 109 | 1 | 0.2 |
| NetSolP (ESM1b) | 0.55 | 0.4258 | 60 | 21 | 19 | 9 | 109 | 0.8696 | 0.525 |
| NetSolP (ESM1b) | 0.6 | 0.3303 | 39 | 31 | 9 | 30 | 109 | 0.5652 | 0.775 |
| NetSolP (ESM1b) | 0.65 | 0.1003 | 5 | 39 | 1 | 64 | 109 | 0.0725 | 0.975 |
| NetSolP (ESM1b) | 0.7 | | 0 | 40 | 0 | 69 | 109 | 0 | 1 |
| NetSolP (ESM1b) | 0.75 | | 0 | 40 | 0 | 69 | 109 | 0 | 1 |
| NetSolP (ESM1b) | 0.8 | | 0 | 40 | 0 | 69 | 109 | 0 | 1 |
| Protein-sol | 0.3 | 0.288 | 69 | 5 | 35 | 0 | 109 | 1 | 0.125 |
| Protein-sol | 0.35 | 0.2855 | 67 | 8 | 32 | 2 | 109 | 0.971 | 0.2 |
| Protein-sol | 0.4 | 0.3834 | 66 | 13 | 27 | 3 | 109 | 0.9565 | 0.325 |

| | | | | | | | | | |
|---|---|---|---|---|---|---|---|---|---|
| Protein-sol | 0.45 | 0.36 | 59 | 19 | 21 | 10 | 109 | 0.8551 | 0.475 |
| Protein-sol | 0.5 | 0.3667 | 50 | 26 | 14 | 19 | 109 | 0.7246 | 0.65 |
| Protein-sol | 0.55 | 0.2899 | 34 | 32 | 8 | 35 | 109 | 0.4928 | 0.8 |
| Protein-sol | 0.6 | 0.2071 | 19 | 36 | 4 | 50 | 109 | 0.2754 | 0.9 |
| Protein-sol | 0.65 | 0.076 | 4 | 39 | 1 | 65 | 109 | 0.058 | 0.975 |
| Protein-sol | 0.7 | -0.1264 | 0 | 39 | 1 | 69 | 109 | 0 | 0.975 |
| Protein-sol | 0.75 | | 0 | 40 | 0 | 69 | 109 | 0 | 1 |
| Protein-sol | 0.8 | | 0 | 40 | 0 | 69 | 109 | 0 | 1 |
| SoluProt | 0.3 | 0.221 | 69 | 3 | 37 | 0 | 109 | 1 | 0.075 |
| SoluProt | 0.35 | 0.2966 | 68 | 7 | 33 | 1 | 109 | 0.9855 | 0.175 |
| SoluProt | 0.4 | 0.3515 | 68 | 9 | 31 | 1 | 109 | 0.9855 | 0.225 |
| SoluProt | 0.45 | 0.3296 | 65 | 12 | 28 | 4 | 109 | 0.942 | 0.3 |
| SoluProt | 0.5 | 0.4901 | 64 | 20 | 20 | 5 | 109 | 0.9275 | 0.5 |
| SoluProt | 0.55 | 0.4324 | 58 | 23 | 17 | 11 | 109 | 0.8406 | 0.575 |
| SoluProt | 0.6 | 0.4866 | 56 | 27 | 13 | 13 | 109 | 0.8116 | 0.675 |
| SoluProt | 0.65 | 0.5068 | 50 | 32 | 8 | 19 | 109 | 0.7246 | 0.8 |
| SoluProt | 0.7 | 0.4453 | 42 | 34 | 6 | 27 | 109 | 0.6087 | 0.85 |
| SoluProt | 0.75 | 0.3323 | 31 | 35 | 5 | 38 | 109 | 0.4493 | 0.875 |
| SoluProt | 0.8 | 0.2361 | 16 | 38 | 2 | 53 | 109 | 0.2319 | 0.95 |

## Calibration curves to quantify product formation

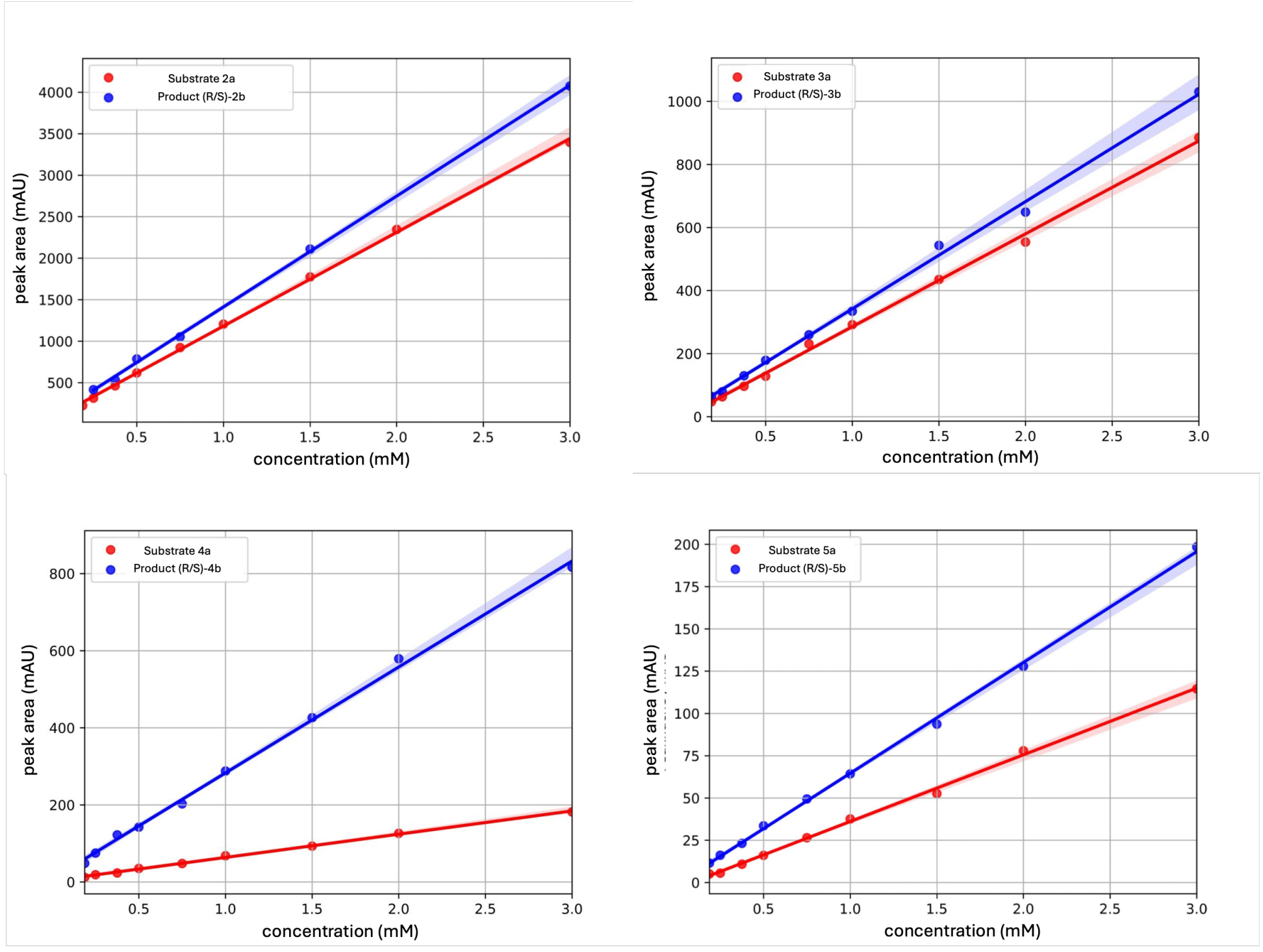


**Figure S5: Product and substrate calibration.** Calibration curves utilized to quantify the formation of products **2b** – **5b**. Quantification of **1b** was performed as previously reported.[12]

## Correlation analysis of stability factors

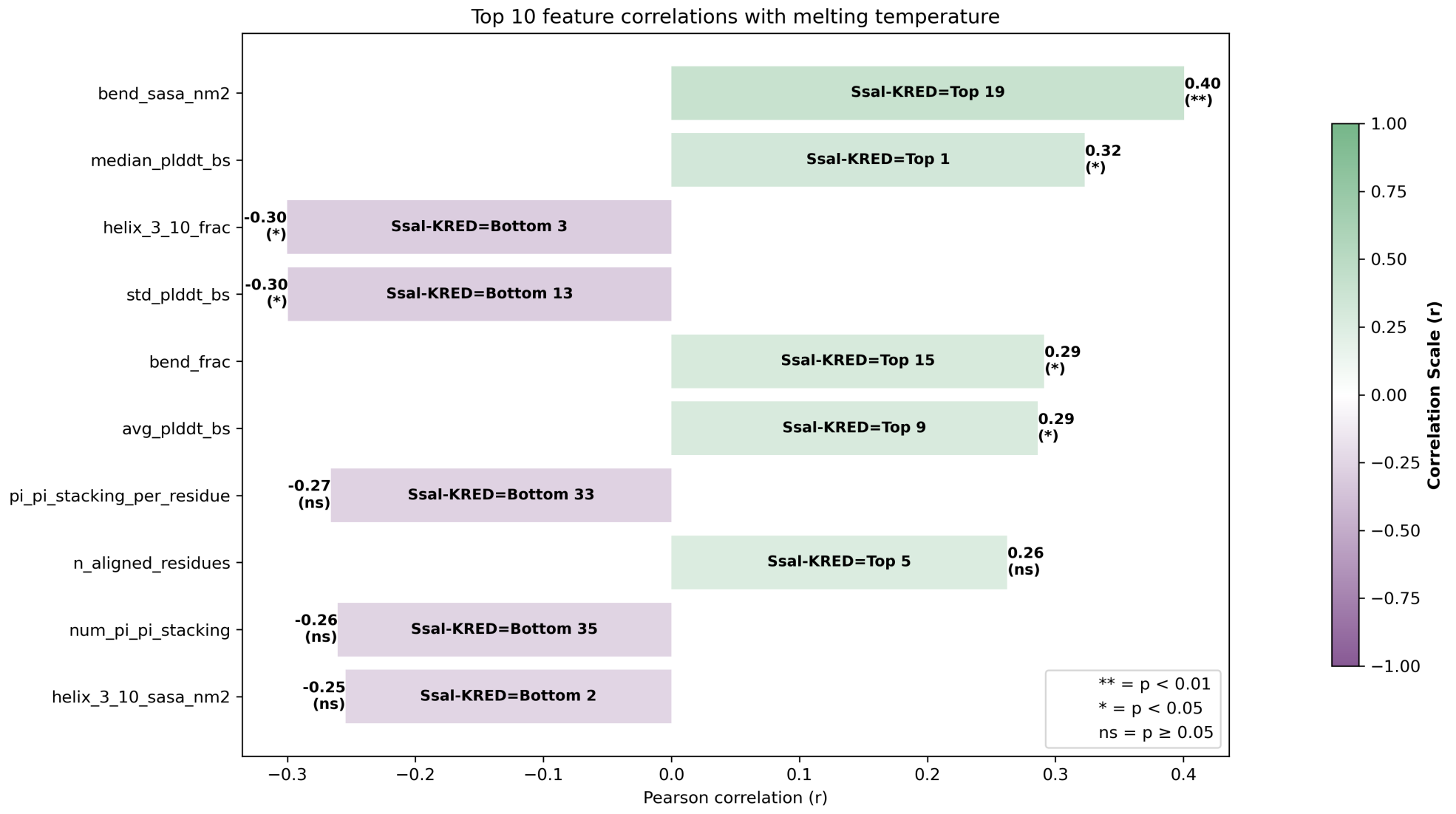


**Figure S6: Top structural features associated with melting temperature.** The ten structure-derived descriptors showing the strongest Pearson correlations with melting temperature across KRED homologs are displayed. The sample size used to derive statistics is defined as n = 54 independent samples for all columns. Statistically significant correlations are indicated on the heatmap as follows: * $p < 0.05$, $p < 0.01$, and *** $p < 0.001$. Bar labels indicate the rank of Ssal-KRED among homologs for each feature. Input data, utilized code and source data underlying the figure can be found on GitHub (https://github.com/Buller-Lab/Ssal-KRED_orthologs/tree/main/Correlation_analysis_stability_factors).

## Statistical analysis of HomoLogic-derived descriptors

**Table S2: Statistical descriptors of HomoLogic-derived descriptors**. Statistical descriptors of HomoLogic-derived descriptors for tested orthologs and homologs from SwissProt database utilizing Ssal-KRED as reference. Input data and utilized code can be found on our GitHub Repository (https://github.com/Buller-Lab/Ssal-KRED_orthologs/tree/main/Statistical_analysis_orthologs_vs_homologs).

| column | group | count | mean | median | std | min | max | skewness | kurtosis |
|---|---|---|---|---|---|---|---|---|---|
| aligned_fraction | orthologs | 63 | 0.4657 | 0.4878 | 0.1721 | 0.1136 | 0.7674 | -0.2 | -1.103 |
| aligned_fraction | swissprot | 24 | 0.415 | 0.4062 | 0.1355 | 0.1489 | 0.7045 | 0.3154 | -0.0416 |
| avg_plddt | orthologs | 63 | 0.95 | 0.958 | 0.0327 | 0.741 | 0.974 | -4.6168 | 25.2704 |
| avg_plddt | swissprot | 24 | 0.9232 | 0.9345 | 0.0383 | 0.8 | 0.975 | -1.5198 | 2.5551 |
| avg_plddt_bs | orthologs | 63 | 0.9584 | 0.968 | 0.0315 | 0.757 | 0.983 | -4.3877 | 24.6814 |
| avg_plddt_bs | swissprot | 24 | 0.9321 | 0.9505 | 0.042 | 0.859 | 0.981 | -0.6667 | -1.115 |
| bindingsite_similarity_score | orthologs | 63 | 0.3037 | 0.3144 | 0.0721 | 0.1528 | 0.4279 | -0.1224 | -1.0779 |
| bindingsite_similarity_score | swissprot | 24 | 0.2609 | 0.2576 | 0.0793 | 0.0917 | 0.4061 | -0.0601 | -0.6234 |
| cavity_area | orthologs | 63 | 370.1295 | 392.7 | 94.0324 | 204.43 | 524.59 | -0.1676 | -1.2601 |
| cavity_area | swissprot | 24 | 407.1167 | 436.795 | 118.3835 | 180.93 | 629.98 | -0.47 | -0.5885 |
| cavity_avg_depth | orthologs | 63 | 1.6106 | 1.56 | 0.3342 | 1.11 | 2.63 | 0.9879 | 0.9444 |
| cavity_avg_depth | swissprot | 24 | 1.8858 | 1.81 | 0.5831 | 0.97 | 3.18 | 0.5629 | -0.2629 |
| cavity_avg_hydropathy | orthologs | 63 | -0.2638 | -0.24 | 0.1577 | -0.63 | 0.1 | -0.2488 | -0.0546 |
| cavity_avg_hydropathy | swissprot | 24 | -0.2275 | -0.24 | 0.1425 | -0.49 | 0.09 | 0.2018 | -0.392 |
| cavity_frequency _Alipathic_apolar | orthologs | 63 | 9.4444 | 10 | 2.4215 | 4 | 15 | -0.3576 | -0.3432 |
| cavity_frequency _Alipathic_apolar | swissprot | 24 | 10.9167 | 11 | 3.309 | 5 | 17 | 0.0012 | -0.874 |
| cavity_frequency_Aromatic | orthologs | 63 | 4.3651 | 4 | 0.9211 | 3 | 7 | 0.714 | 0.6358 |
| cavity_frequency_Aromatic | swissprot | 24 | 3.0833 | 3 | 1.0598 | 2 | 5 | 0.2809 | -1.3309 |
| cavity_frequency _Negatively_charged | orthologs | 63 | 0.6032 | 1 | 0.5547 | 0 | 2 | 0.1572 | -0.9179 |
| cavity_frequency _Negatively_charged | swissprot | 24 | 1.0833 | 1 | 0.8805 | 0 | 3 | 0.6198 | -0.1263 |
| cavity_frequency _Non-standard | orthologs | 63 | 0 | 0 | 0 | 0 | 0 | | |
| cavity_frequency _Non-standard | swissprot | 24 | 0 | 0 | 0 | 0 | 0 | | |
| cavity_frequency _Polar_uncharged | orthologs | 63 | 7.9365 | 8 | 1.874 | 5 | 14 | 0.478 | 0.0586 |
| cavity_frequency _Polar_uncharged | swissprot | 24 | 8.2917 | 8 | 2.255 | 4 | 14 | 0.7439 | 0.6687 |
| cavity_frequency _Positively_charged | orthologs | 63 | 2.3492 | 3 | 1.0342 | 1 | 4 | -0.0317 | -1.2301 |
| cavity_frequency _Positively_charged | swissprot | 24 | 2.7917 | 3 | 1.1788 | 1 | 5 | -0.4016 | -0.7373 |
| cavity_n_points | orthologs | 63 | 2433.397 | 2360 | 526.2873 | 1171 | 3768 | 0.4465 | 0.1285 |
| cavity_n_points | swissprot | 24 | 2925.708 | 3133.5 | 875.4526 | 1140 | 4838 | -0.1133 | -0.3297 |
| cavity_rmsd | orthologs | 63 | 0.4987 | 0.514 | 0.0633 | 0.314 | 0.628 | -1.1492 | 1.8501 |
| cavity_rmsd | swissprot | 24 | 0.5071 | 0.5185 | 0.0843 | 0.303 | 0.689 | -0.416 | 0.3562 |
| cavity_volume | orthologs | 63 | 512.5644 | 526.39 | 143.3047 | 252.94 | 827.06 | 0.1454 | -0.7452 |
| cavity_volume | swissprot | 24 | 630.8733 | 688.28 | 223.3299 | 246.24 | 1101.38 | -0.1555 | -0.674 |
| iqr_plddt | orthologs | 63 | 0.0387 | 0.029 | 0.0447 | 0.015 | 0.352 | 5.7598 | 36.904 |
| iqr_plddt | swissprot | 24 | 0.0631 | 0.046 | 0.0565 | 0.014 | 0.298 | 3.1703 | 10.6967 |
| iqr_plddt_bs | orthologs | 63 | 0.0329 | 0.021 | 0.0403 | 0.006 | 0.312 | 5.4484 | 34.8726 |

| | | | | | | | | | |
|---|---|---|---|---|---|---|---|---|---|
| iqr_plddt_bs | swissprot | 24 | 0.0495 | 0.037 | 0.0347 | 0.006 | 0.152 | 1.3543 | 1.4133 |
| median_plddt | orthologs | 63 | 0.97 | 0.976 | 0.0248 | 0.795 | 0.984 | -5.855 | 38.1023 |
| median_plddt | swissprot | 24 | 0.9597 | 0.963 | 0.0163 | 0.914 | 0.984 | -1.0923 | 0.9358 |
| median_plddt_bs | orthologs | 63 | 0.9728 | 0.978 | 0.0208 | 0.835 | 0.987 | -4.934 | 28.938 |
| median_plddt_bs | swissprot | 24 | 0.9627 | 0.9705 | 0.0173 | 0.93 | 0.987 | -0.5525 | -1.0564 |
| n_aligned_residues | orthologs | 63 | 20.3492 | 20 | 7.7712 | 5 | 33 | -0.1358 | -1.2145 |
| n_aligned_residues | swissprot | 24 | 18.5417 | 18 | 5.9416 | 7 | 31 | 0.2943 | -0.1902 |
| n_bindingsite_residues | orthologs | 63 | 43.5556 | 44 | 2.2272 | 38 | 50 | -0.0477 | 0.0953 |
| n_bindingsite_residues | swissprot | 24 | 44.875 | 45 | 2.6591 | 39 | 50 | -0.0188 | -0.1798 |
| n_res_plddt | orthologs | 63 | 387.5238 | 389 | 12.2657 | 341 | 441 | -0.3925 | 9.944 |
| n_res_plddt | swissprot | 24 | 395.125 | 393.5 | 37.0115 | 265 | 494 | -1.0255 | 6.327 |
| n_res_plddt_bs | orthologs | 63 | 43.5556 | 44 | 2.2272 | 38 | 50 | -0.0477 | 0.0953 |
| n_res_plddt_bs | swissprot | 24 | 44.875 | 45 | 2.6591 | 39 | 50 | -0.0188 | -0.1798 |
| non_equivalent_residues | orthologs | 63 | 7 | 7 | 1.1914 | 5 | 11 | 0.3461 | 0.6446 |
| non_equivalent_residues | swissprot | 24 | 7.9167 | 7 | 2.5862 | 4 | 15 | 0.9872 | 0.9307 |
| p10_plddt | orthologs | 63 | 0.8934 | 0.911 | 0.0788 | 0.452 | 0.952 | -3.8493 | 16.6382 |
| p10_plddt | swissprot | 24 | 0.8094 | 0.862 | 0.1347 | 0.431 | 0.95 | -1.462 | 1.3153 |
| p10_plddt_bs | orthologs | 63 | 0.9126 | 0.935 | 0.0753 | 0.434 | 0.973 | -4.2899 | 24.0946 |
| p10_plddt_bs | swissprot | 24 | 0.8088 | 0.899 | 0.1736 | 0.456 | 0.969 | -0.9781 | -0.6704 |
| rmsd | orthologs | 63 | 2.7751 | 2.74 | 0.1628 | 2.36 | 3.22 | 0.2522 | 0.6229 |
| rmsd | swissprot | 24 | 2.7229 | 2.76 | 0.2104 | 1.92 | 3.02 | -2.2792 | 6.9672 |
| rmsd_bindingsite | orthologs | 63 | 0.6693 | 0.6483 | 0.2057 | 0.1404 | 1.1226 | -0.0578 | -0.4247 |
| rmsd_bindingsite | swissprot | 24 | 0.6228 | 0.6173 | 0.158 | 0.3747 | 1.0316 | 0.9388 | 0.7887 |
| sequence_identity | orthologs | 63 | 0.2807 | 0.286 | 0.0169 | 0.219 | 0.3 | -1.2389 | 1.7111 |
| sequence_identity | swissprot | 24 | 0.281 | 0.286 | 0.0138 | 0.256 | 0.299 | -0.3136 | -1.2479 |
| std_plddt | orthologs | 63 | 0.0599 | 0.054 | 0.0272 | 0.026 | 0.187 | 2.4876 | 7.7606 |
| std_plddt | swissprot | 24 | 0.0982 | 0.0885 | 0.0472 | 0.025 | 0.219 | 0.7521 | 0.1006 |
| std_plddt_bs | orthologs | 63 | 0.0381 | 0.029 | 0.03 | 0.009 | 0.19 | 2.5558 | 9.0536 |
| std_plddt_bs | swissprot | 24 | 0.0766 | 0.058 | 0.0603 | 0.013 | 0.18 | 0.7193 | -1.0732 |
| tm_score | orthologs | 63 | 0.8383 | 0.8454 | 0.0227 | 0.7452 | 0.8761 | -1.807 | 4.4696 |
| tm_score | swissprot | 24 | 0.8046 | 0.818 | 0.0552 | 0.6357 | 0.9256 | -0.9356 | 2.4258 |

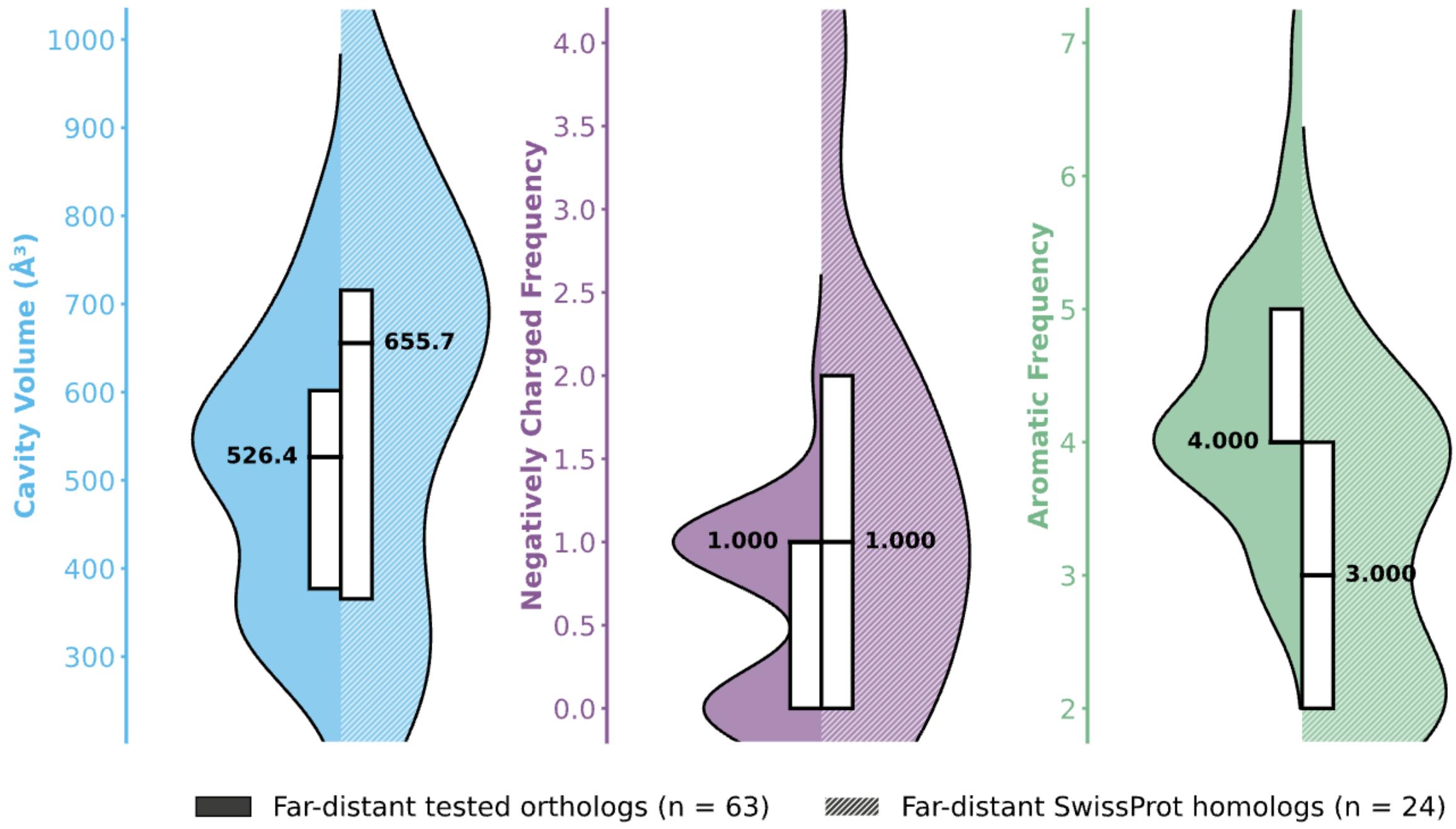


**Figure S7: Cavity property distributions of tested orthologs vs. SwissProt homologs.** Far-distant orthologs (SeqID <= 30 %) display significantly shifted cavity volume, as well as a different frequency of negatively charged and aromatic residues in the binding-site compared to far-distant SwissProt homologs (SeqID <= 30%). Within each split violin, a white box plot marks the median (horizontal line with text annotation) and the interquartile range (25th–75th percentiles). The median of all distributions is specified by a label next to the box plot within the violin representation. Input data and utilized code can be found on our GitHub Repository (https://github.com/Buller-Lab/Ssal-KRED_orthologs/tree/main/Splitted_histogram_Orthologs_vs_SwissProt).

**Table S3: Welch test of HomoLogic descriptors: orthologs vs. SwissProt homologs.** Welch test results comparing average values of our HomoLogic-derived descriptors for both tested orthologs and homologs from SwissProt[13] database utilizing Ssal-KRED as reference. Statistical significance was evaluated using two-sided, unadjusted Welch's two-sample t-tests ($\alpha = 0.05$). Reported metrics include exact test statistics (t), group sample sizes (n), group means, and exact P-values without adjustment for multiple comparisons. Input data and utilized code can be found on our GitHub Repository (https://github.com/Buller-Lab/Ssal-KRED_orthologs/tree/main/Statistical_analysis_orthologs_vs_homologs).

| *column* | *average (orthologs)* | *average (swissprot)* | *t_stat* | *p_value* | *significant (p < 0.05)* | *n_orthologs* | *n_swissprot* |
|---|---|---|---|---|---|---|---|
| *cavity_frequency_Aromatic* | 4.3651 | 3.0833 | 5.2211 | 0.000007 | WAHR | 63 | 24 |
| *std_plddt* | 0.0599 | 0.0982 | -3.7479 | 0.000789 | WAHR | 63 | 24 |
| *avg_plddt* | 0.95 | 0.9232 | 3.0331 | 0.004437 | WAHR | 63 | 24 |
| *std_plddt_bs* | 0.0381 | 0.0766 | -2.9859 | 0.005885 | WAHR | 63 | 24 |
| *p10_plddt* | 0.8934 | 0.8094 | 2.8747 | 0.007471 | WAHR | 63 | 24 |
| *tm_score* | 0.8383 | 0.8046 | 2.8967 | 0.007548 | WAHR | 63 | 24 |
| *avg_plddt_bs* | 0.9584 | 0.9321 | 2.7864 | 0.008723 | WAHR | 63 | 24 |
| *p10_plddt_bs* | 0.9126 | 0.8088 | 2.8309 | 0.008771 | WAHR | 63 | 24 |
| *cavity_n_points* | 2433.397 | 2925.708 | -2.5829 | 0.015001 | WAHR | 63 | 24 |
| *cavity_frequency _Negatively_charged* | 0.6032 | 1.0833 | -2.4898 | 0.018505 | WAHR | 63 | 24 |
| *cavity_volume* | 512.5644 | 630.8733 | -2.4129 | 0.022036 | WAHR | 63 | 24 |
| *median_plddt_bs* | 0.9728 | 0.9627 | 2.2896 | 0.026323 | WAHR | 63 | 24 |
| *bindingsite_similarity_score* | 0.3037 | 0.2609 | 2.3038 | 0.026744 | WAHR | 63 | 24 |
| *median_plddt* | 0.97 | 0.9597 | 2.2676 | 0.026788 | WAHR | 63 | 24 |
| *cavity_avg_depth* | 1.6106 | 1.8858 | -2.1798 | 0.037554 | WAHR | 63 | 24 |
| *n_bindingsite_residues* | 43.5556 | 44.875 | -2.1594 | 0.037561 | WAHR | 63 | 24 |
| *n_res_plddt_bs* | 43.5556 | 44.875 | -2.1594 | 0.037561 | WAHR | 63 | 24 |
| *cavity_frequency _Alipathic_apolar* | 9.4444 | 10.9167 | -1.9864 | 0.055386 | FALSCH | 63 | 24 |
| *iqr_plddt_bs* | 0.0329 | 0.0495 | -1.905 | 0.062785 | FALSCH | 63 | 24 |
| *iqr_plddt* | 0.0387 | 0.0631 | -1.9026 | 0.065451 | FALSCH | 63 | 24 |
| *non_equivalent_residues* | 7 | 7.9167 | -1.6702 | 0.106514 | FALSCH | 63 | 24 |
| *cavity_frequency _Positively_charged* | 2.3492 | 2.7917 | -1.617 | 0.114311 | FALSCH | 63 | 24 |
| *aligned_fraction* | 0.4657 | 0.415 | 1.4418 | 0.155282 | FALSCH | 63 | 24 |
| *cavity_area* | 370.1295 | 407.1167 | -1.3743 | 0.178164 | FALSCH | 63 | 24 |
| *n_aligned_residues* | 20.3492 | 18.5417 | 1.1596 | 0.251276 | FALSCH | 63 | 24 |
| *rmsd_bindingsite* | 0.6693 | 0.6228 | 1.1236 | 0.266156 | FALSCH | 63 | 24 |
| *rmsd* | 2.7751 | 2.7229 | 1.096 | 0.280782 | FALSCH | 63 | 24 |
| *cavity_avg_hydropathy* | -0.2638 | -0.2275 | -1.0309 | 0.307986 | FALSCH | 63 | 24 |
| *n_res_plddt* | 387.5238 | 395.125 | -0.9857 | 0.333738 | FALSCH | 63 | 24 |
| *cavity_frequency _Polar_uncharged* | 7.9365 | 8.2917 | -0.6865 | 0.496807 | FALSCH | 63 | 24 |
| *cavity_rmsd* | 0.4987 | 0.5071 | -0.4433 | 0.660402 | FALSCH | 63 | 24 |
| *sequence_identity* | 0.2807 | 0.281 | -0.1016 | 0.91945 | FALSCH | 63 | 24 |
| *cavity_frequency _Non-standard* | 0 | 0 | | | FALSCH | 63 | 24 |

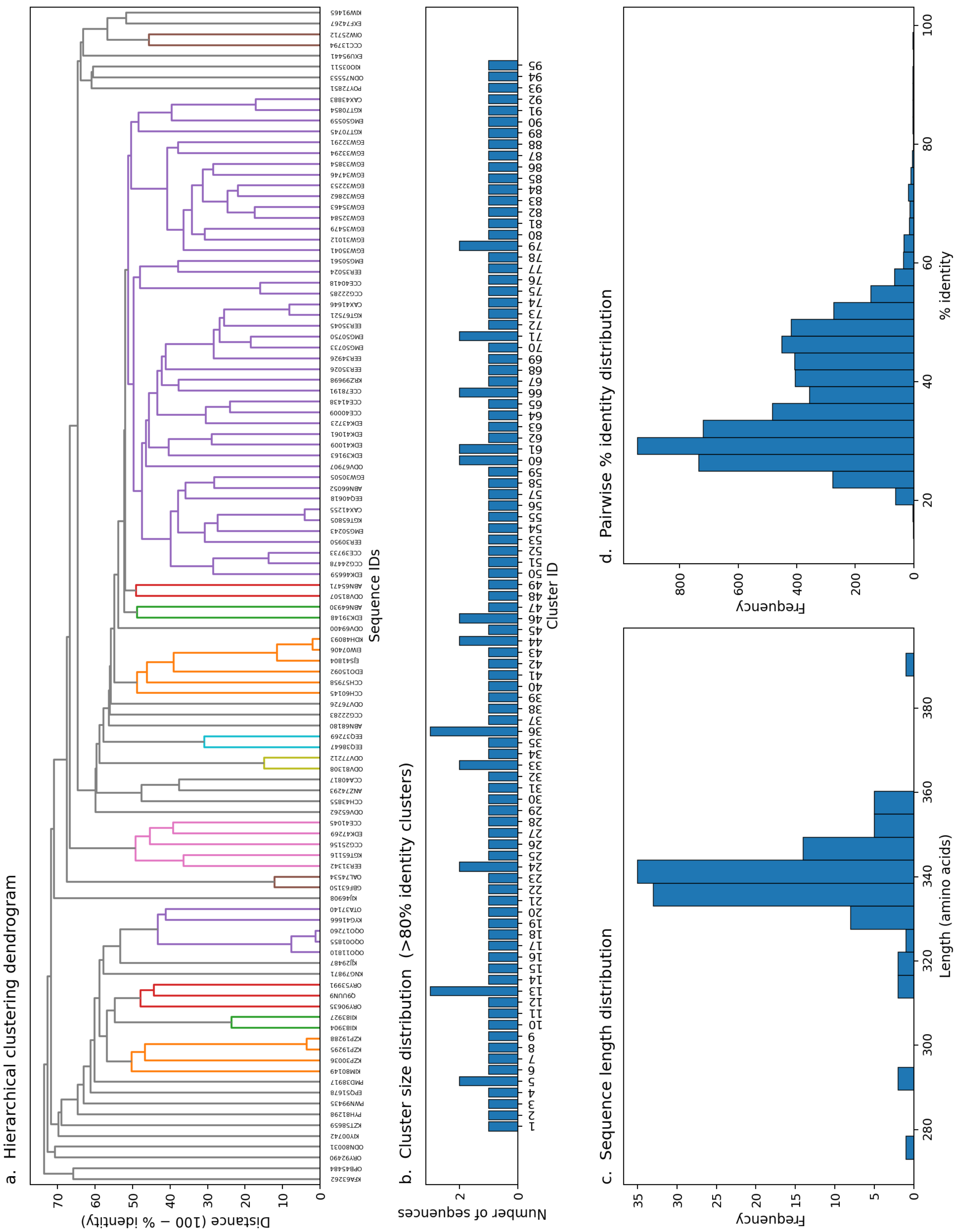


**Figure S8: Quality assessment of sequence similarity of mined orthologs. (a)** Hierarchical clustering dendrogram based on pairwise global alignment identity, **(b)** distribution of cluster sizes at ~80% identity threshold, **(c)** sequence length distribution, and **(d)** distribution of all pairwise percent identities (excluding self-comparisons). Input data and utilized code can be found on GitHub (https://github.com/Buller-Lab/Ssal-KRED_orthologs/tree/main/Ortholog_sequences_distribution_analysis).

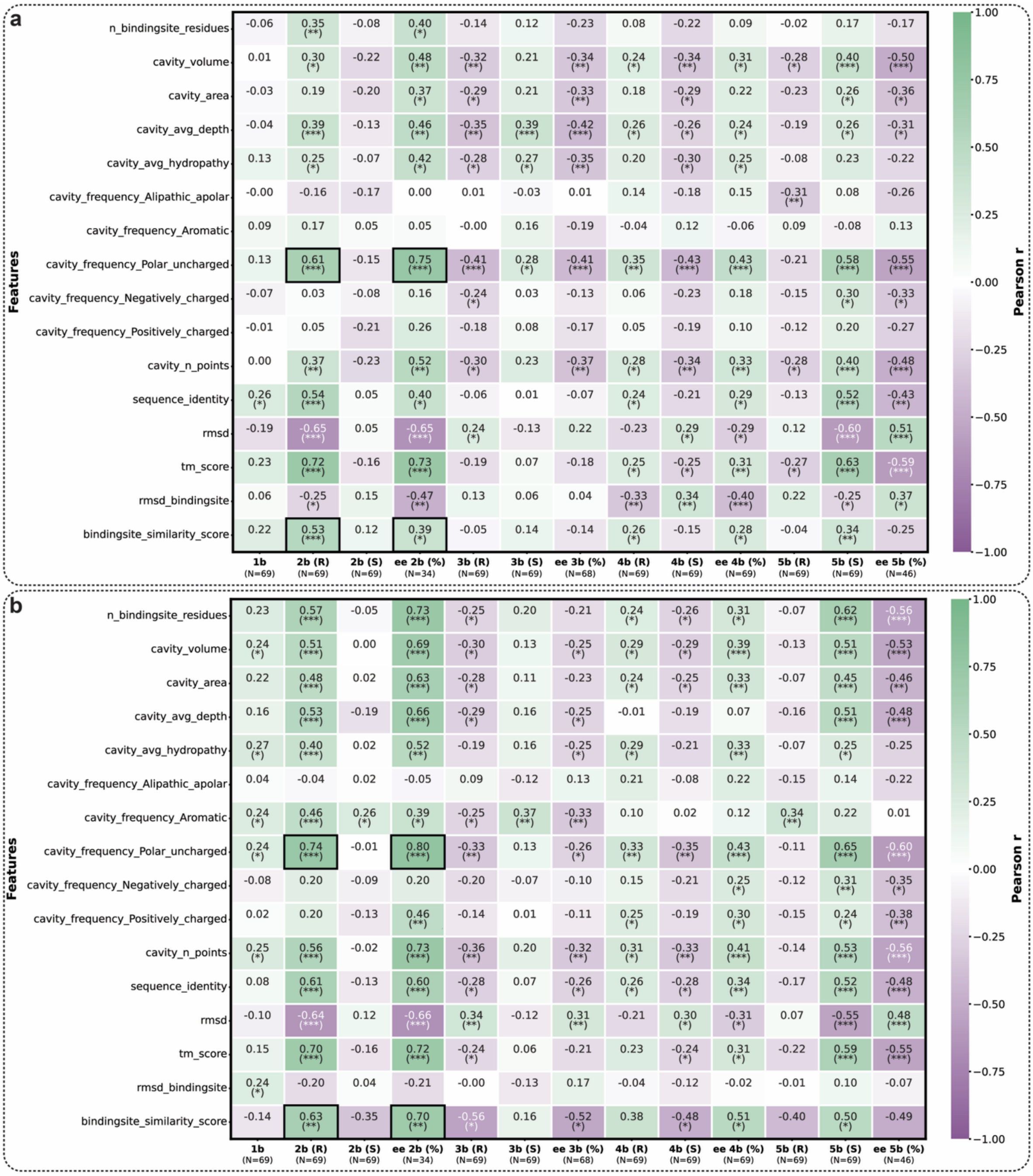

**Figure S9: Single-feature predictivity of HomoLogic-derived descriptors.** Heatmap showing pairwise Pearson correlation coefficients (r) between diverse biocatalytic target parameters and HomoLogic-derived descriptors calculated using **(a)** Ssal-KRED modeled with **1a** and **(b)** Ort-RDM-1 modeled with **2a**. Statistical significance was determined using two-tailed Pearson correlation tests. Sample sizes used to derive statistics are defined per column, with n = 34 independent samples for *ee* **2b** (%), n = 68 independent samples for *ee* **3b** (%), n = 46 independent samples for *ee* **5b** (%), and n = 69 independent samples for all remaining columns. Statistically significant correlations are indicated on the heatmap as follows: * $p < 0.05$, ** $p < 0.01$, and *** $p < 0.001$. Input data and utilized code can be found on our GitHub Repository (https://github.com/Buller-Lab/Ssal-KRED_orthologs/).

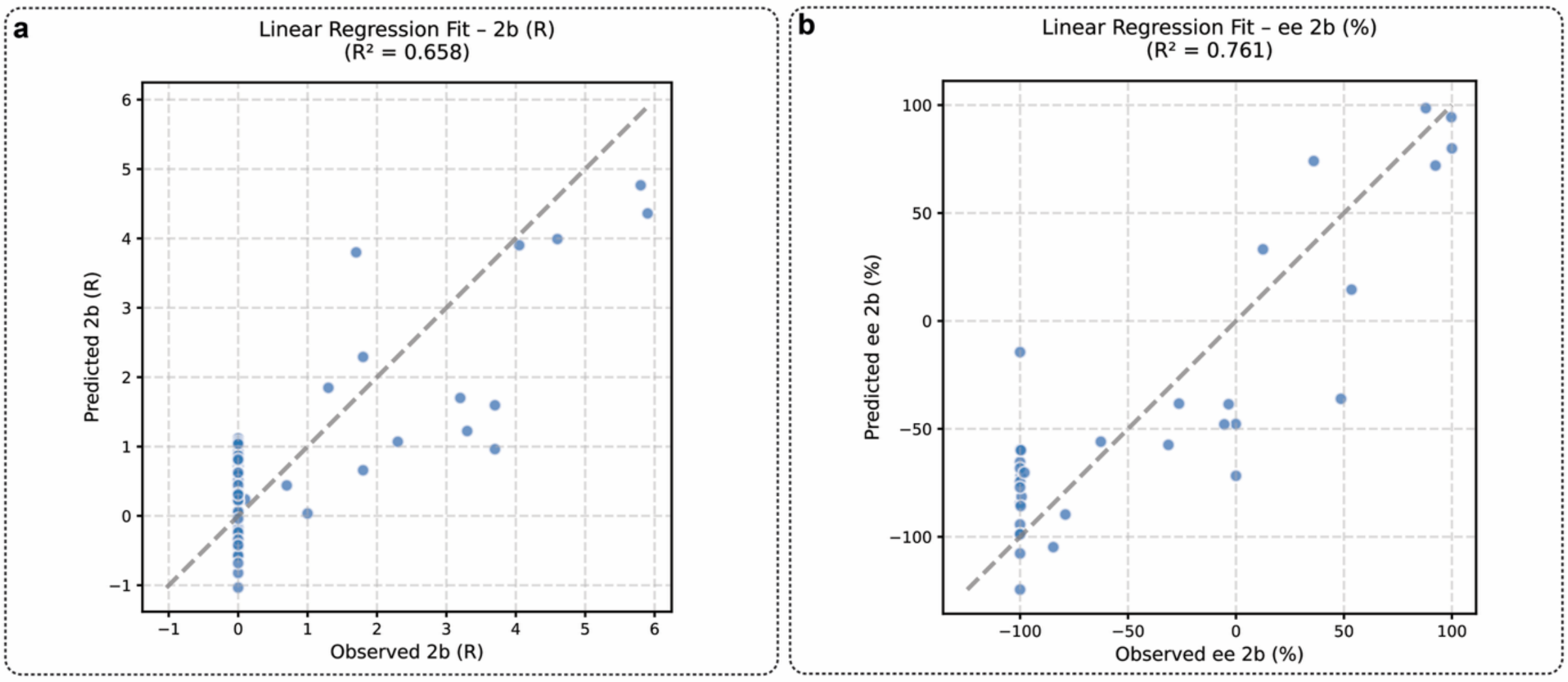


**Figure S10: Exploration of predictive performance for explainable machine-learning models.** Multiple linear regression models trained on 3 Lasso features for prediction of **(a)** product formation of **2b** (*R)* and **(b)** enantioselectivity towards substrate **2a**. Input data and utilized code can be found on our GitHub Repository (https://github.com/Buller-Lab/Ssal-KRED_orthologs/tree/main/Multiple_linear_regression_and_PCA).

**Table S4: Three-feature model performance for 2b (R) across sequence-identity thresholds.** Performance of the three-feature linear regression model for 2b *(R)* product formation after MMseqs2[14]-based homology reduction (easy-cluster) at different sequence identity thresholds. Input data and utilized code can be found on our GitHub Repository (https://github.com/Buller-Lab/Ssal-KRED_orthologs/tree/main/Regressor_generalization_capacity).

| *threshold* | *n_remaining* | *r2_full* | *adj_r2* | *rmse_full* | *q2_loo* | *rmse_loo* | *mae_loo* | *pcc_loo* | *delta_r2_q2* |
|---|---|---|---|---|---|---|---|---|---|
| *90%* | 67 | 0.657 | 0.641 | 0.853 | 0.59 | 0.933 | 0.71 | 0.769 | 0.067 |
| *80%* | 61 | 0.667 | 0.65 | 0.873 | 0.598 | 0.96 | 0.749 | 0.774 | 0.07 |
| *70%* | 48 | 0.631 | 0.605 | 0.909 | 0.537 | 1.018 | 0.823 | 0.737 | 0.093 |
| *60%* | 36 | 0.648 | 0.615 | 0.921 | 0.525 | 1.069 | 0.868 | 0.731 | 0.123 |
| *50%* | 22 | 0.675 | 0.621 | 0.945 | 0.464 | 1.212 | 1.047 | 0.697 | 0.21 |

**Table S5: Three-feature model performance for ee 2b (%) across sequence-identity thresholds.** Performance of the three-feature linear regression model for *ee* **2b** (%) after MMseqs2[14]-based homology reduction (easy-cluster) at different sequence identity thresholds. Input data and utilized code can be found on our GitHub Repository (https://github.com/Buller-Lab/Ssal-KRED_orthologs/tree/main/Regressor_generalization_capacity).

| *threshold* | *n_remaining* | *r2_full* | *adj_r2* | *rmse_full* | *q2_loo* | *rmse_loo* | *mae_loo* | *pcc_loo* | *delta_r2_q2* |
|---|---|---|---|---|---|---|---|---|---|
| *90%* | 33 | 0.76 | 0.735 | 34.674 | 0.71 | 38.129 | 31.236 | 0.843 | 0.05 |
| *80%* | 32 | 0.755 | 0.729 | 35.181 | 0.702 | 38.788 | 32.107 | 0.838 | 0.053 |
| *70%* | 29 | 0.73 | 0.698 | 35.425 | 0.655 | 40.08 | 32.691 | 0.811 | 0.075 |
| *60%* | 26 | 0.785 | 0.756 | 31.907 | 0.715 | 36.748 | 30.251 | 0.847 | 0.07 |
| *50%* | 15 | 0.72 | 0.644 | 34.831 | 0.582 | 42.585 | 35.361 | 0.77 | 0.138 |

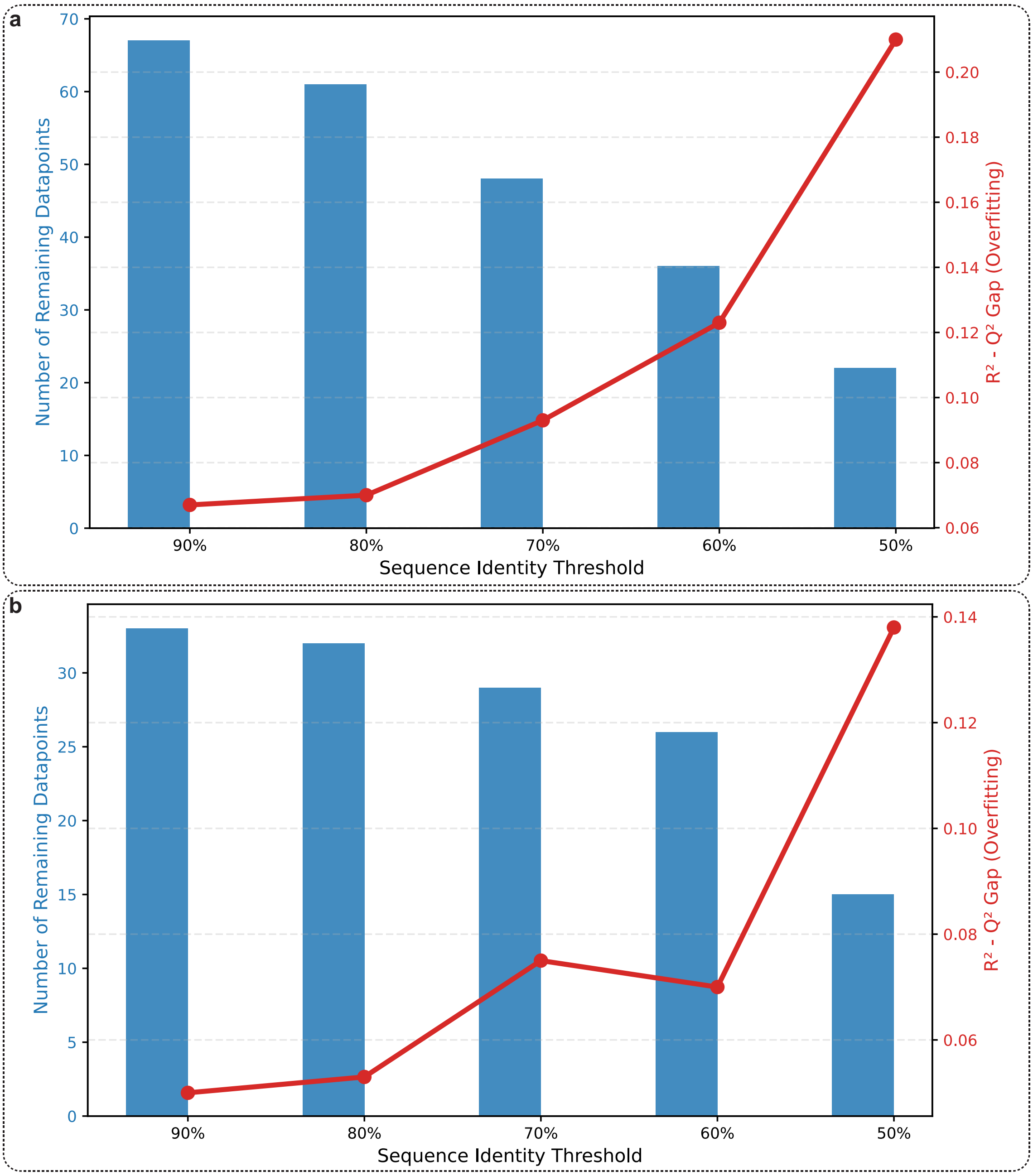


**Figure S11: Generalization capacity towards distant homologs.** Evaluation of generalization capacity via removal of sequence identity clusters for **(a)** product formation of **2b** (*R)* and **(b)** enantioselectivity towards substrate **2a**. Input data, utilized code, and source data of the plots can be found on our GitHub Repository (https://github.com/Buller-Lab/Ssal-KRED_orthologs/tree/main/Regressor_generalization_capacity).

**Supplementary References**


1. Price, M. N., Dehal, P. S. & Arkin, A. P. FastTree 2 – Approximately Maximum-Likelihood Trees for Large Alignments. *PLOS ONE* **5**, e9490 (2010).
2. Katoh, K. & Standley, D. M. MAFFT Multiple Sequence Alignment Software Version 7: Improvements in Performance and Usability. *Mol. Biol. Evol.* **30**, 772–780 (2013).
3. Letunic, I. & Bork, P. Interactive Tree of Life (iTOL) v6: recent updates to the phylogenetic tree display and annotation tool. *Nucleic Acids Res.* **52**, W78–W82 (2024).
4. Wagih, O. ggseqlogo: a versatile R package for drawing sequence logos. *Bioinformatics* **33**, 3645–3647 (2017).
5. The UniProt Consortium *et al.* UniProt: the Universal Protein Knowledgebase in 2025. *Nucleic Acids Res.* **53**, D609–D617 (2025).
6. Blum, M. *et al.* InterPro: the protein sequence classification resource in 2025. *Nucleic Acids Res.* **53**, D444–D456 (2025).
7. Hon, J. *et al.* SoluProt: prediction of soluble protein expression in *Escherichia coli*. *Bioinformatics* **37**, 23–28 (2021).
8. Hebditch, M., Carballo-Amador, M. A., Charonis, S., Curtis, R. & Warwicker, J. Protein-Sol: A web tool for predicting protein solubility from sequence. *Bioinformatics* https://doi.org/10.1093/bioinformatics/btx345 (2017) doi:10.1093/bioinformatics/btx345.
9. Thumuluri, V. *et al.* NetSolP: predicting protein solubility in Escherichia coli using language models. *Bioinformatics* **38**, 941–946 (2022).
10. Hunter, J. D. Matplotlib: A 2D Graphics Environment. *Comput. Sci. Eng.* **9**, 90–95 (2007).
11. Pedregosa, F. *et al.* Scikit-learn: Machine Learning in Python. https://doi.org/10.48550/ARXIV.1201.0490 (2012) doi:10.48550/ARXIV.1201.0490.
12. Honda Malca, S. *et al.* Effective engineering of a ketoreductase for the biocatalytic synthesis of an ipatasertib precursor. *Commun. Chem.* **7**, 46 (2024).

13. Gasteiger, E., Jung, E. & Bairoch, A. SWISS-PROT: connecting biomolecular knowledge via a protein database. *Curr. Issues Mol. Biol.* **3**, 47–55 (2001).
14. Steinegger, M. & Söding, J. MMseqs2 enables sensitive protein sequence searching for the analysis of massive data sets. *Nat. Biotechnol.* **35**, 1026–1028 (2017).
