# Supplementary material for: Iterative and data-driven ortholog mining enables reliable discovery of stereoselective ketoreductases
Source: Nat Commun. 2026 Sep 15;17:10854. doi: 10.1038/s41467-026-77715-6 (PMC13672348; doi:10.1038/s41467-026-77715-6)
Supplement: Supplementary file 2 [file 41467_2026_77715_MOESM2_ESM.pdf]

Description of Additional Supplementary Files

Supplementary Data 1: Spreadsheet listing all genome assembly identifiers and database accessions utilized for Ssal-KRED orthogroup definition.

Supplementary Data 2: Multi-FASTA format sequence file containing the full set of 4,181 identified ketoreductase sequences of the Ssal-KRED orthogroup.

Supplementary Data 3: Master spreadsheet containing the complete list of tested orthologs alongside all biocatalytic conversion measurements, enantioselectivities, and experimental results.

Supplementary Data 4: Spreadsheet containing the raw and calculated numerical values used to construct Figure S1, S2, S4 – S11.
