# Supplementary material for: Iterative and data-driven ortholog mining enables reliable discovery of stereoselective ketoreductases
Source: Nat Commun. 2026 Sep 15;17:10854. doi: 10.1038/s41467-026-77715-6 (PMC13672348; doi:10.1038/s41467-026-77715-6)
Supplement: Supplementary file 3 [file 41467_2026_77715_MOESM7_ESM.pdf]

nature portfolio

Corresponding author(s): Rebecca Buller

Last updated by author(s): Aug 24, 2026

Reporting Summary

Nature Portfolio wishes to improve the reproducibility of the work that we publish. This form provides structure for consistency and transparency in reporting. For further information on Nature Portfolio policies, see our Editorial Policies and the Editorial Policy Checklist.

Statistics

For all statistical analyses, confirm that the following items are present in the figure legend, table legend, main text, or Methods section.

n/a	Confirmed	
☐	☒	The exact sample size (*n*) for each experimental group/condition, given as a discrete number and unit of measurement
☐	☒	A statement on whether measurements were taken from distinct samples or whether the same sample was measured repeatedly
☐	☒	The statistical test(s) used AND whether they are one- or two-sided<br>*Only common tests should be described solely by name; describe more complex techniques in the Methods section.*
☐	☒	A description of all covariates tested
☐	☒	A description of any assumptions or corrections, such as tests of normality and adjustment for multiple comparisons
☐	☒	A full description of the statistical parameters including central tendency (e.g. means) or other basic estimates (e.g. regression coefficient) AND variation (e.g. standard deviation) or associated estimates of uncertainty (e.g. confidence intervals)
☐	☒	For null hypothesis testing, the test statistic (e.g. *F*, *t*, *r*) with confidence intervals, effect sizes, degrees of freedom and *P* value noted<br>*Give P values as exact values whenever suitable.*
☒	☐	For Bayesian analysis, information on the choice of priors and Markov chain Monte Carlo settings
☒	☐	For hierarchical and complex designs, identification of the appropriate level for tests and full reporting of outcomes
☐	☒	Estimates of effect sizes (e.g. Cohen's *d*, Pearson's *r*), indicating how they were calculated

Our web collection on statistics for biologists contains articles on many of the points above.

Software and code

Policy information about availability of computer code

Data collection	OpenLab CDS Acquisition v2.4 and v2.8, Spark Control (3.1), MassLynx v4.1, PR.Panta Analysis (v1.6.3), Boltz-2 (v2.2.0), OrthoFinder (v2), EnzymeMiner (v1)
Data analysis	OpenLab CDS Data Analysis v2.7, MassLynx v4.1, PR.Panta Analysis (v1.6.3), Pymol 2.5.2, Excel version 2502, FastTree (v2.2.0), Biopython (v1.84), scikit-bio (v0.7.0), Clustal Omega (v1.2.3), scipy (v1.16.3/v1.17.0), seaborn (v0.13.2), matplotlib (v3.10.8), pandas (v2.3.3/v3.0.0), numpy (v2.4.2), scikit-learn (v1.8.0), Python 3.14.2

For manuscripts utilizing custom algorithms or software that are central to the research but not yet described in published literature, software must be made available to editors and reviewers. We strongly encourage code deposition in a community repository (e.g. GitHub). See the Nature Portfolio guidelines for submitting code & software for further information.

Data

Policy information about availability of data

All manuscripts must include a data availability statement. This statement should provide the following information, where applicable:

- Accession codes, unique identifiers, or web links for publicly available datasets
- A description of any restrictions on data availability
- For clinical datasets or third party data, please ensure that the statement adheres to our policy

The experimental data generated in this study, including biocatalytic data (GC/LC peak data, conversions, enantiomeric and diastereomeric excesses, and calibration

curves) and enzyme melting temperatures, have been deposited on Zenodo (https://doi.org/10.5281/zenodo.19574983). Third-party sequence data utilized for ortholog and homolog searches were obtained from the publicly available Ensembl Genomes, SwissProt and UniProt databases; the resulting extracted sequences and search outputs are provided within the accompanying GitHub repository (https://github.com/Buller-Lab/Ssal-KRED_orthologs). This repository also contains the source data and input files required for generating the plots and performing post-analysis. For long-term accessibility, the repository has been versioned and deposited on Zenodo (https://doi.org/10.5281/zenodo.20756197. A list with all genome assembly identifiers utilized for orthogroup definition ("Supplementary Data 1"), the fasta file of the full Ssal-KRED orthogroup ("Supplementary Data 2"), and an overview of all tested orthologs with experimental results ("Supplementary Data 3") are available as Supplementary Data. Experimental raw data and methods are provided with this paper, the Supplementary Information or are available from the authors upon request. Source data are provided with this paper.

Research involving human participants, their data, or biological material

Policy information about studies with human participants or human data. See also policy information about sex, gender (identity/presentation), and sexual orientation and race, ethnicity and racism.

Reporting on sex and gender	not applicable
Reporting on race, ethnicity, or other socially relevant groupings	not applicable
Population characteristics	not applicable
Recruitment	not applicable
Ethics oversight	not applicable

Note that full information on the approval of the study protocol must also be provided in the manuscript.

Field-specific reporting

Please select the one below that is the best fit for your research. If you are not sure, read the appropriate sections before making your selection.

☒ Life sciences ☐ Behavioural & social sciences ☐ Ecological, evolutionary & environmental sciences

For a reference copy of the document with all sections, see nature.com/documents/nr-reporting-summary-flat.pdf

Life sciences study design

All studies must disclose on these points even when the disclosure is negative.

Sample size	No statistical methods were used to pre-determine sample size.<br>For experimental characterization, all biocatalytic reactions were performed in biological triplicates.Three replicate measurements are sufficient to have confidence in average measured values and to determine a standard deviation.
Data exclusions	No data were excluded from the analysis.
Replication	All enzyme activity measurements and melting temperature (Tm) determinations were performed in biological triplicates (n=3). Biological replicates were generated by picking three independent colonies for each enzyme followed by separate growth of pre-cultures and main cultures, and preparation of individual bacterial lysates. The mean value of the three biological replicates is reported for each enzyme (or enzyme–substrate pair).<br><br>Assay quality and reproducibility for both activity and Tm measurements were continuously monitored by including two negative controls (empty vector) and three positive controls (Ssal-KRED) on each plate. These intra-plate controls allowed assessment of background activity, signal stability, plate-to-plate variation<br><br>All attempts at replication were successful.
Randomization	Randomization was not relevant because no experimental groups were assigned; all measurements were performed under standardized conditions.
Blinding	This study does not involve human/ animal subjects and goup allocation, therefore blinding was not necessary. Nevertheless, appropriate control experiments were performed.

Reporting for specific materials, systems and methods

We require information from authors about some types of materials, experimental systems and methods used in many studies. Here, indicate whether each material, system or method listed is relevant to your study. If you are not sure if a list item applies to your research, read the appropriate section before selecting a response.

Materials & experimental systems

n/a	Involved in the study
☒	☐ Antibodies
☒	☐ Eukaryotic cell lines
☒	☐ Palaeontology and archaeology
☒	☐ Animals and other organisms
☒	☐ Clinical data
☒	☐ Dual use research of concern
☒	☐ Plants

Methods

n/a	Involved in the study
☒	☐ ChIP-seq
☒	☐ Flow cytometry
☒	☐ MRI-based neuroimaging

Plants

Seed stocks	not applicable
Novel plant genotypes	not applicable
Authentication	not applicable
