# Supplementary material for: Iterative and data-driven ortholog mining enables reliable discovery of stereoselective ketoreductases
Source: Nat Commun. 2026 Sep 15;17:10854. doi: 10.1038/s41467-026-77715-6 (PMC13672348; doi:10.1038/s41467-026-77715-6)
Supplement: Supplementary file 4 [file 41467_2026_77715_MOESM8_ESM.pdf]

nature portfolio

Peer Review File

Iterative and data-driven ortholog mining enables reliable discovery of stereoselective ketoreductases

Corresponding Author: Professor Rebecca Buller

This file contains all reviewer reports in order by version, followed by all author rebuttals in order by version.

Version 0:

Reviewer comments:

Reviewer #1

(Remarks to the Author)
In this manuscript, Buller and coworkers present a novel approach to discovering stereoselective ketoreductases (KREDs) by coupling ortholog mining with machine learning. The authors successfully identify multiple highly selective biocatalysts for the synthesis of pharmaceutically relevant chiral alcohols. This work establishes an informative workflow that stands out from previous strategies by utilizing an iterative sampling strategy to mine orthologs. Furthermore, to overcome the reliance of sequenced genomes, the authors introduce data-driven approaches using the ortholog screening as input. HomoLogic, an automated tool to extract functional descriptors for predictive modeling is developed to facilitate the practise. Overall, the authors demonstrate a practical and transferable enzyme mining strategy and I recommend the manuscript for publication after the authors address a few issues listed below.

1. To enhance the clarity of the study, the authors should include a conceptual diagram (potentially as Figure 1) that explicitly outlines the enzyme mining workflow, highlighting how it fundamentally diverges from existing methods. Similarly, the machine learning section requires a flowchart to illustrate the input data, feature extraction, and processing algorithms.

2. The authors note that all identified hits exhibit lower melting temperatures compared to the parent Ssal-KRED. Please elaborate on the underlying reasons for this observation.

3. The success of the enzyme mining is evaluated based on reaction conversion. However, conversion is a combined result of kcat, Km, and overall enzyme stability. As the enzyme are primarily mined based on microscopic features, wouldn’t they correlate better with kintetic data? Could the authors provide a rationale for this?

(Remarks on code availability)

Reviewer #2

(Remarks to the Author)
In this manuscript, Stockinger et al. describe an iterative ortholog-mining strategy for the discovery of stereoselective ketoreductases (KREDs). Starting from a previously engineered KRED scaffold, the authors perform two rounds of ortholog sampling and experimentally characterize more than one hundred candidates. The study further introduces HomoLogic, a computational pipeline that extracts sequence-, structure-, and binding-site-based descriptors and uses these features to build interpretable machine learning models for predicting KRED activity and stereoselectivity.

The combination of ortholog mining, experimental validation, and descriptor-based modeling is interesting and potentially valuable for enzyme discovery. The manuscript is generally well written, the experimental work is extensive, and several highly selective KREDs were identified. However, there are several comments that should be addressed before the manuscript can be considered for publication.

I recommend adding a graphical overview of the proposed workflow. A schematic figure summarizing the complete enzyme discovery pipeline, from ortholog mining and experimental screening to HomoLogic-based descriptor generation and ML-

assisted candidate prioritization, would greatly improve the accessibility of the manuscript.

In lines 255–257, the authors state: “Of note, the orthologs exhibited significantly higher binding-site similarity between each other compared to the set of homologs (Figure 3c; average score 0.30 vs. 0.26; P = 0.02)”. However, this should refer to Figure 3b rather than Figure 3c. In addition, the reported binding-site similarity values appear inconsistent with the values shown in the figure (approximately 0.31 vs. 0.26 rather than 0.30 vs. 0.26). Please check and correct accordingly.

The statements regarding cavity volume, aromatic residue frequency, and negatively charged residue frequency (lines 257–259) are not supported by any figure. Please provide the corresponding data visualization and statistical comparisons, either in Figure 3 or in the Supplementary Information.

The central computational claim of the manuscript is that HomoLogic-derived descriptors can accurately predict enzyme activity and enantioselectivity. However, all reported evaluations appear to be based on the same ortholog dataset used for model development and therefore do not adequately demonstrate the model's ability to generalize to truly unseen sequence space. The authors are encouraged to perform cluster-based cross-validation, in which closely related sequences are grouped before data splitting, to provide a more rigorous assessment of model generalizability.

Given the growing interest in AI-driven enzyme discovery, the authors may consider expanding the Conclusion and Discussion section to include recent advances in this area. In particular, it would be useful to cite recent review articles, such as Yuan et al., "Enzyme Property Prediction Using Artificial Intelligence" (Current Opinion in Chemical Engineering, 2026, 51:101208), which provides a comprehensive overview of AI approaches for enzyme property prediction and discovery.

(Remarks on code availability)
The code is publicly available, well documented, and easy to navigate. The README provides sufficient instructions for installation and use, and the repository should be a valuable resource for the community. Overall, the code availability and documentation are satisfactory.

Reviewer #3

(Remarks to the Author)
I recommend publication of the manuscript, “Iterative and data-driven ortholog mining enables reliable discovery of stereoselective ketoreductases,” pending minor revisions.

The authors present a compelling study describing the development of an efficient workflow for generating a panel of novel ketoreductases that exhibit promiscuous activity and stereoselectivity toward a set of substrates relevant to the pharmaceutical industry. The workflow is based on iterative application of computational tools to filter for progressively more stringent, yet increasingly important, enzyme properties in each “round,” incorporating the intuition and logic of an expert biocatalysis practitioner into the machine-learning model HomoLogic. Each round narrows the set of ketoreductase sequences to those that better fit the desired criteria, and the results are corroborated by screening data that support the validity of the approach.

The authors describe the workflow steps in detail and clearly demonstrate the success of their approach on relevant substrates. Overall, the data is thorough, the conclusions are well supported, the figures effectively illustrate the findings, and the references are recent and appropriately comprehensive. The supplementary information provides useful additional details and validation methods.

The described workflow represents an efficient approach to enzyme discovery and provides valuable starting points for further development. It is of considerable importance and relevance to the scientific community in the fields of biocatalysis and enzyme engineering, as it enables functional and property fingerprinting at the early stages of identifying enzymes for specific applications. In doing so, it increases the quality of the enzyme panel and the likelihood of success for a given application. This is also important to the field as a strategy for mining previously undefined enzymes or novel reactions through a combined experimental and computational effort, thereby accelerating enzyme discovery in a field that continues to grow in importance as a major methodology in synthetic chemistry.

I recommend that the authors expand their discussion of the leading predictors of activity in their case study. While the authors propose reasonable explanations for binding-site-specific metrics affecting the predictability of enantioselectivity, they do not speculate beyond polarity and pi-stacking potential on why and how the frequency of polar residues or aromatic residues in the active-site cavity so strongly influences activity prediction. Since activity prediction is central to new enzyme discovery, the rationale behind these properties would likely be of interest to readers.

Finally, please check references 35 and 36, as they appear to be duplicates by accident.

(Remarks on code availability)

Version 1:

Reviewer comments:

Reviewer #1

(Remarks to the Author)
The authors have addressed all my suggestions/questions and the revision has further strengthened the paper. I am satisfied with the revision and recommend publication in Nat. Commun.

(Remarks on code availability)

Reviewer #2

(Remarks to the Author)
The authors have satisfactorily addressed all of my previous comments and concerns, and the quality and clarity of the manuscript have been significantly improved. I have no further comments and believe that the manuscript is suitable for publication in Nature Communications.

(Remarks on code availability)

Reviewer #3

(Remarks to the Author)
Recommendation: Accept the revised manuscript "Iterative and data-driven ortholog mining enables reliable discovery of stereoselective ketoreductases," for publication.
The authors have strengthened the manuscript by adding text that expands the mechanistic rationale underlying the relationship among their best-performing parameters. In particular, the new observation that the models consistently group residues according to chemical properties is noteworthy, and the accompanying discussion of how specific residue characteristics may interact with the corresponding substrates is especially interesting. These chemically intuitive features support the idea that HomoLogic captures meaningful differences in active-site chemistry and overall structure.

(Remarks on code availability)

Open Access This Peer Review File is licensed under a Creative Commons Attribution 4.0 International License, which permits use, sharing, adaptation, distribution and reproduction in any medium or format, as long as you give appropriate credit to the original author(s) and the source, provide a link to the Creative Commons license, and indicate if changes were made.
In cases where reviewers are anonymous, credit should be given to 'Anonymous Referee' and the source.
The images or other third party material in this Peer Review File are included in the article's Creative Commons license,

unless indicated otherwise in a credit line to the material. If material is not included in the article's Creative Commons license and your intended use is not permitted by statutory regulation or exceeds the permitted use, you will need to obtain permission directly from the copyright holder.
To view a copy of this license, visit https://creativecommons.org/licenses/by/4.0/

Reviewer #1

In this manuscript, Buller and coworkers present a novel approach to discovering stereoselective ketoreductases (KREDs) by coupling ortholog mining with machine learning. The authors successfully identify multiple highly selective biocatalysts for the synthesis of pharmaceutically relevant chiral alcohols. This work establishes an informative workflow that stands out from previous strategies by utilizing an iterative sampling strategy to mine orthologs. Furthermore, to overcome the reliance of sequenced genomes, the authors introduce data-driven approaches using the ortholog screening as input. HomoLogic, an automated tool to extract functional descriptors for predictive modeling is developed to facilitate the practise. Overall, the authors demonstrate a practical and transferable enzyme mining strategy and I recommend the manuscript for publication after the authors address a few issues listed below.

We thank the reviewer for this positive evaluation of our work and for the constructive suggestions for further optimizing our manuscript.

Comment 1
To enhance the clarity of the study, the authors should include a conceptual diagram (potentially as Figure 1) that explicitly outlines the enzyme mining workflow, highlighting how it fundamentally diverges from existing methods. Similarly, the machine learning section requires a flowchart to illustrate the input data, feature extraction, and processing algorithms.

We thank the reviewer for this constructive suggestion. We agree that incorporating visual overviews will improve the clarity of our manuscript for a broader audience. In accordance with your recommendation, we have added Figure 1 to provide a conceptual diagram that illustrates our iterative enzyme mining workflow. Furthermore, we have introduced a flowchart (now integrated as Figure 5) that maps out the *HomoLogic*-workflow.

Comment 2
The authors note that all identified hits exhibit lower melting temperatures compared to the parent Ssal-KRED. Please elaborate on the underlying reasons for this observation.

We thank the reviewer for raising this interesting question regarding the thermal stability differences. In short, we believe that Ssal-KRED stands out as exceptionally stable. All other orthologs including the less active ones proved less stable, consistent with their phylogenetic origin in mesophilic fungi.

However, to analyze this aspect in more detail, we derived several additional structural features with potential impact on protein stability, analyzed their correlation with all available melting temperatures (n=54), and examined Ssal-KRED's ranking across them. Our analysis indicates that Ssal-KRED has increased local rigidity: it ranks highest in median pLDDT around the binding-site with comparably low pLDDT variability, which can be seen as a proxy for rigidity. Moreover it has the third lowest fraction of 3_{10}-helices (which are known to be less stable than normal alpha helices), and shows comparably high bend fractions (which are usually more stable than loops). The isolated correlation of these features was weak (< 0.4) but significant ($p < 0.05$). We have added a brief paragraph in the according section referencing Supplementary Figure S6 which shows the top 10 correlating features highlighting the rank of Ssal-KREDs in the overall dataset:

To rationalize these considerable differences in melting temperature, we derived additional structural features with suspected relevance for protein stability and analyzed their correlation with the available melting temperatures of all orthologs (Supplementary Figure S6). Our analysis suggests that Ssal-KRED possesses increased local rigidity in the binding-site region and beneficial secondary structure elements. Specifically, it ranks highest in median binding-site pLDDT while exhibiting low binding-site pLDDT variability (pLDDT reasonably correlates with conventional flexibility metrics). It also displays the third-

lowest fraction of 3_{10}-helices and comparably high bend fractions with greater solvent-exposed surface area (typically α-helices are more stable than 3_{10}-helices while bends are more stable than loops). Although the individual correlations between these features and the measured melting temperature were relatively weak (Pearson's $r < 0.4$), they reached statistical significance in our dataset ($P < 0.05$; $n = 54$).

Comment 3

The success of the enzyme mining is evaluated based on reaction conversion. However, conversion is a combined result of kcat, Km, and overall enzyme stability. As the enzyme are primarily mined based on microscopic features, wouldn't they correlate better with kinetic data? Could the authors provide a rationale for this?

We thank the reviewer for this insightful comment. The reviewer is of course entirely correct that microscopic structural features govern intrinsic enzymatic kinetic parameters (k_{cat} and K_m), and we agree that separately analyzing the correlations between structural descriptors and protein production yields, k_{cat}, K_m, and specific activities would be a valuable line of investigation.

Our decision to evaluate success on the basis of "effective activity" (conversion in lysates) rather than kinetic parameters of purified enzymes was deliberate, and reflects the specific application and data requirements of our pipeline. Three considerations motivated this choice :

1. First, the practical performance of an enzyme depends not only on its specific activity but also on how well it is produced, and these two properties can be improved through complementary routes: enzyme engineering and expression optimization. Screening directly in lysate captures both contributions simultaneously. This avoids biasing selection against variants with high specific activity but low production yields, ensuring that the candidates we identify are genuinely viable for practical application.
2. Our approach depends on machine learning models, which perform best with large, consistent datasets. Determining true kinetic parameters (k_{cat}, K_m) would have required purifying and testing over 100 variants, constituting a throughput-determining experimental bottleneck that would have substantially reduced the size of our training dataset. Effective activity provided us with a consistent, scalable readout across all variants.
3. HomoLogic descriptors are not restricted to kinetic determinants; by comparing variants at the sequence and structure level, they capture broader features relevant to overall functional performance, including stability and solubility. Conversion in lysate is therefore the label most consistent with what these descriptors encode. We additionally use dedicated solubility and stability predictors to set operational thresholds, allowing users to filter out poorly behaved variants while minimizing false negatives.

Reviewer #2

In this manuscript, Stockinger et al. describe an iterative ortholog-mining strategy for the discovery of stereoselective ketoreductases (KREDs). Starting from a previously engineered KRED scaffold, the authors perform two rounds of ortholog sampling and experimentally characterize more than one hundred candidates. The study further introduces HomoLogic, a computational pipeline that extracts sequence-, structure-, and binding-site-based descriptors and uses these features to build interpretable machine learning models for predicting KRED activity and stereoselectivity.

The combination of ortholog mining, experimental validation, and descriptor-based modeling is interesting and potentially valuable for enzyme discovery. The manuscript is generally well written, the experimental work is extensive, and several highly selective KREDs were identified. However, there are several comments that should be addressed before the manuscript can be considered for publication.

We thank the reviewer for this positive assessment of our work. We have carefully addressed all comments and believe that the revised manuscript has been strengthened as a result.

Comment 1
I recommend adding a graphical overview of the proposed workflow. A schematic figure summarizing the complete enzyme discovery pipeline, from ortholog mining and experimental screening to HomoLogic-based descriptor generation and ML-assisted candidate prioritization, would greatly improve the accessibility of the manuscript.

We appreciate this recommendation, which aligns with the feedback from Reviewer #1. As noted in our response to Reviewer #1, we have added two graphical overviews. Please see Figure 1 and 5.

Comment 2
In lines 255–257, the authors state: "Of note, the orthologs exhibited significantly higher binding-site similarity between each other compared to the set of homologs (Figure 3c; average score 0.30 vs. 0.26; P = 0.02)". However, this should refer to Figure 3b rather than Figure 3c. In addition, the reported binding-site similarity values appear inconsistent with the values shown in the figure (approximately 0.31 vs. 0.26 rather than 0.30 vs. 0.26). Please check and correct accordingly.

We thank the reviewer for their careful reading of our manuscript and for catching these errors. We have corrected the text to reference **Figure 3b** instead of Figure 3c. Furthermore, we have updated the values to **0.31 vs. 0.26**.

Comment 3
The statements regarding cavity volume, aromatic residue frequency, and negatively charged residue frequency (lines 257–259) are not supported by any figure. Please provide the corresponding data visualization and statistical comparisons, either in Figure 3 or in the Supplementary Information.

We apologize for this oversight. We agree that providing the underlying data visualization will help to support our claims. We have generated the corresponding plots and statistical comparisons for cavity volume, aromatic residue frequencies, and negative charge distributions. These visualizations have been compiled into a new **Supplementary Figure S8**. Moreover Figure S8, as well as Table S3 are now referenced in the main text.

Comment 5

The central computational claim of the manuscript is that HomoLogic-derived descriptors can accurately predict enzyme activity and enantioselectivity. However, all reported evaluations appear to be based on the same ortholog dataset used for model development and therefore do not adequately demonstrate the model's ability to generalize to truly unseen sequence space. The authors are encouraged to perform cluster-based cross-validation, in which closely related sequences are grouped before data splitting, to provide a more rigorous assessment of model generalizability.

We thank the reviewer for highlighting the importance of evaluating generalization to unseen sequence space. In response, we used cluster-based redundancy reduction (MMseqs2 easy-cluster) at progressively stricter sequence identity thresholds (90%, 80%, 70%, 60%, and 50%) to build increasingly non-redundant datasets, and re-evaluated on each. Because only one representative is retained per cluster, the representatives within each dataset are mutually dissimilar below the corresponding threshold. Consequently, in the subsequent leave-one-out cross validation (LOO-CV) every held out sequence has no close homolog remaining in the training set, precisely the condition that the reviewer's suggested cluster-based cross validation is designed to create. The procedure therefore delivers the requested stringent test of generalizability. To ensure that any change in performance reflects reduces redundancy rather than re-tuning, we kept the model definition fixed throughout (the same three LASSO-selected features and linear regression). The results (Supplementary Tables S4-S5 and Supplementary Figure S11) show that predictive performance remains stable as homologous sequences are removed. Notably, the absolute predictive accuracy is retained: Q^2 for 2b product formation and ee (%) remained ≥ 0.525 down to 60% identity, compared with Q^2 = 0.59 at 90 % identity. Accordingly, the R^2-Q^2 gap, our measure of overfitting, stayed below 0.14 for ee 2b (%) down to 15 representative sequences (50% identity)and below 0.12 for 2b (R) down to 36 representatives (60% identity), increasing only moderately at 50% identity. We did not cluster below 50% sequence identity, as the number of remaining datapoints became too low for reliable LOO-CV. The robustness is consistent with our featurization strategy. Although sequence identity was available as a candidate feature, it was not among the three features selected by LASSO; the models instead relied on structural (TM-score) and binding site similarity. Because predictions are driven by structural and active-site relatedness rather than raw sequence identity, the models retain accuracy even when close sequence homologs are removed, exactly the behaviour to generalize to distant homologs. These observations and their implications are now discussed in a newly added paragraph:

To probe generalization to unseen sequence space, we performed redundancy reduction via MMseqs2 clustering at sequence identity thresholds of 90%, 80%, 70%, 60%, and 50% retaining one centroid representative per cluster, followed by re-evaluation through LOO-CV using the same fixed three-feature models. Because the retained representatives are mutually dissimilar below each threshold, every held-out sequence lacks a close homolog in the training and can be used to assess generalizability. Predictive performance remained remarkably stable despite the progressive removal of homologous sequences (Supplementary Tables S4-S5; Supplementary Figure S11) and, notably, the absolute predictive accuracy was retained: Q^2 for 2b product formation and ee (%) remained ≥ 0.525 down to 60% identity, compared with Q^2 = 0.59 at 90 % identity. Consistently, the R^2–Q^2 gap, our measure of overfitting, stayed below 0.14 for ee 2b (%) down to 15 representatives (50% identity) and below 0.12 for 2b (R) production down to 36 representatives (60% identity). We did not cluster below 50% identity, as the number of remaining datapoints became too low for reliable LOO-CV. This robustness is consistent with our featurization strategy, which deliberately exploits structural and binding-site homologies rather than raw sequence similarity to identify promising enzymes. Although sequence identity was available as a candidate feature, it was not selected among the top three by LASSO in either model; instead, the models relied primarily on structural (TM-score) and binding-site similarity. Because predictions are therefore driven by structural and active-site relatedness rather than sequence identity, the models retain accuracy even when close sequence homologs are removed, a behavior expected to generalize toward distant homologs.

Comment 6

Given the growing interest in AI-driven enzyme discovery, the authors may consider expanding the Conclusion and Discussion section to include recent advances in this area. In particular, it would be useful to cite recent review articles, such as Yuan et al., "Enzyme Property Prediction Using Artificial Intelligence" (Current Opinion in Chemical Engineering, 2026, 51:101208), which provides a comprehensive overview of AI approaches for enzyme property prediction and discovery.

We thank the reviewer for directing our attention to this relevant literature. We have expanded our Conclusion and Discussion section to contextualize our strategy within the landscape of AI-driven enzyme discovery. Specifically, we have integrated extended our conclusion with a paragraph on recent machine learning approaches for property prediction and have cited the review by Yuan et al.

In this context, AI-driven property prediction tools offer complementary capabilities for enzyme selection by forecasting key parameters such as thermostability, solubility, optimal pH, and aggregation propensity.[63,76] An example of this integration is EnzymeMiner 2.0, which pairs large-scale sequence mining with property predictions and filtering to isolate variants tailored to specific user goals.[77]. By enabling pre-experimental prioritization of candidates, the models accelerate the overall enzyme mining pipeline while, as demonstrated here, empirical insights from ortholog mining rounds can be used to establish enzyme-family-specific cutoffs for predicted properties.

Comment 7

The code is publicly available, well documented, and easy to navigate. The README provides sufficient instructions for installation and use, and the repository should be a valuable resource for the community. Overall, the code availability and documentation are satisfactory.

Thank you for this positive feedback.

Reviewer #3

I recommend publication of the manuscript, "Iterative and data-driven ortholog mining enables reliable discovery of stereoselective ketoreductases," pending minor revisions.

The authors present a compelling study describing the development of an efficient workflow for generating a panel of novel ketoreductases that exhibit promiscuous activity and stereoselectivity toward a set of substrates relevant to the pharmaceutical industry. The workflow is based on iterative application of computational tools to filter for progressively more stringent, yet increasingly important, enzyme properties in each "round," incorporating the intuition and logic of an expert biocatalysis practitioner into the machine-learning model HomoLogic. Each round narrows the set of ketoreductase sequences to those that better fit the desired criteria, and the results are corroborated by screening data that support the validity of the approach.

The authors describe the workflow steps in detail and clearly demonstrate the success of their approach on relevant substrates. Overall, the data is thorough, the conclusions are well supported, the figures effectively illustrate the findings, and the references are recent and appropriately comprehensive. The supplementary information provides useful additional details and validation methods.

The described workflow represents an efficient approach to enzyme discovery and provides valuable starting points for further development. It is of considerable importance and relevance to the scientific community in the fields of biocatalysis and enzyme engineering, as it enables functional and property fingerprinting at the early stages of identifying enzymes for specific applications. In doing so, it increases the quality of the enzyme panel and the likelihood of success for a given application. This is also important to the field as a strategy for mining previously undefined enzymes or novel reactions through a combined experimental and computational effort, thereby accelerating enzyme discovery in a field that continues to grow in importance as a major methodology in synthetic chemistry.

We thank the reviewer for their thorough evaluation and for recognizing the value of our iterative, data-driven ortholog mining strategy and the HomoLogic pipeline for the biocatalysis community.

Comment 1
I recommend that the authors expand their discussion of the leading predictors of activity in their case study. While the authors propose reasonable explanations for binding-site-specific metrics affecting the predictability of enantioselectivity, they do not speculate beyond polarity and pi-stacking potential on why and how the frequency of polar residues or aromatic residues in the active-site cavity so strongly influences activity prediction. Since activity prediction is central to new enzyme discovery, the rationale behind these properties would likely be of interest to readers.

We thank the reviewer for encouraging us to elaborate on the mechanistic rationale behind our top-performing features. We agree that that these relationships are of interest to the reader and have therefore added the following rationalization into the corresponding praragraph:

The models consistently selected cavity-composition features (e.g., polar uncharged and aromatic residue) alongside global structural similarity, allowing mechanistic interpretations. Given that 2b is the aromatic ketone 1-phenylethanone, the positive influence of aromatic residues in the active site likely reflects π-stacking interactions with the phenyl ring, while uncharged polar residues may facilitate hydrogen bonding with the carbonyl oxygen, anchoring the substrate in a productive orientation. That such chemically intuitive features emerge from the selection underscores the capacity of the descriptors to capture functionally relevant differences in active-site polarity, π-stacking potential, and overall structural fold.

That said, we have deliberately refrained from over-interpreting the role of individual amino acid positions and interactions with the substrate reflecting the design intention of the *HomoLogic* framework, which is to bypass case-by-case structural speculation. Rather than modeling specific interactions, HomoLogic has been build to extract predictive value from feature patterns across diverse sequence space while preserving model interpretability through global feature importances.

Comment 2

Finally, please check references 35 and 36, as they appear to be duplicates by accident.

Thanks for bringing this error to our attention. We have checked our reference library, corrected the duplicate entry, and updated the bibliography:

35. Stamboulian, M., Guerrero, R. F., Hahn, M. W. & Radivojac, P. The ortholog conjecture revisited: the value of orthologs and paralogs in function prediction. Bioinformatics 36, i219–i226 (2020).

36. Altenhoff, A. M., Studer, R. A., Robinson-Rechavi, M. & Dessimoz, C. Resolving the Ortholog Conjecture: Orthologs Tend to Be Weakly, but Significantly, More Similar in Function than Paralogs. PLOS Comput. Biol. 8, e1002514 (2012).
