# Supplementary material for: Iterative and data-driven ortholog mining enables reliable discovery of stereoselective ketoreductases
Source: Nat Commun. 2026 Sep 15;17:10854. doi: 10.1038/s41467-026-77715-6 (PMC13672348; doi:10.1038/s41467-026-77715-6)

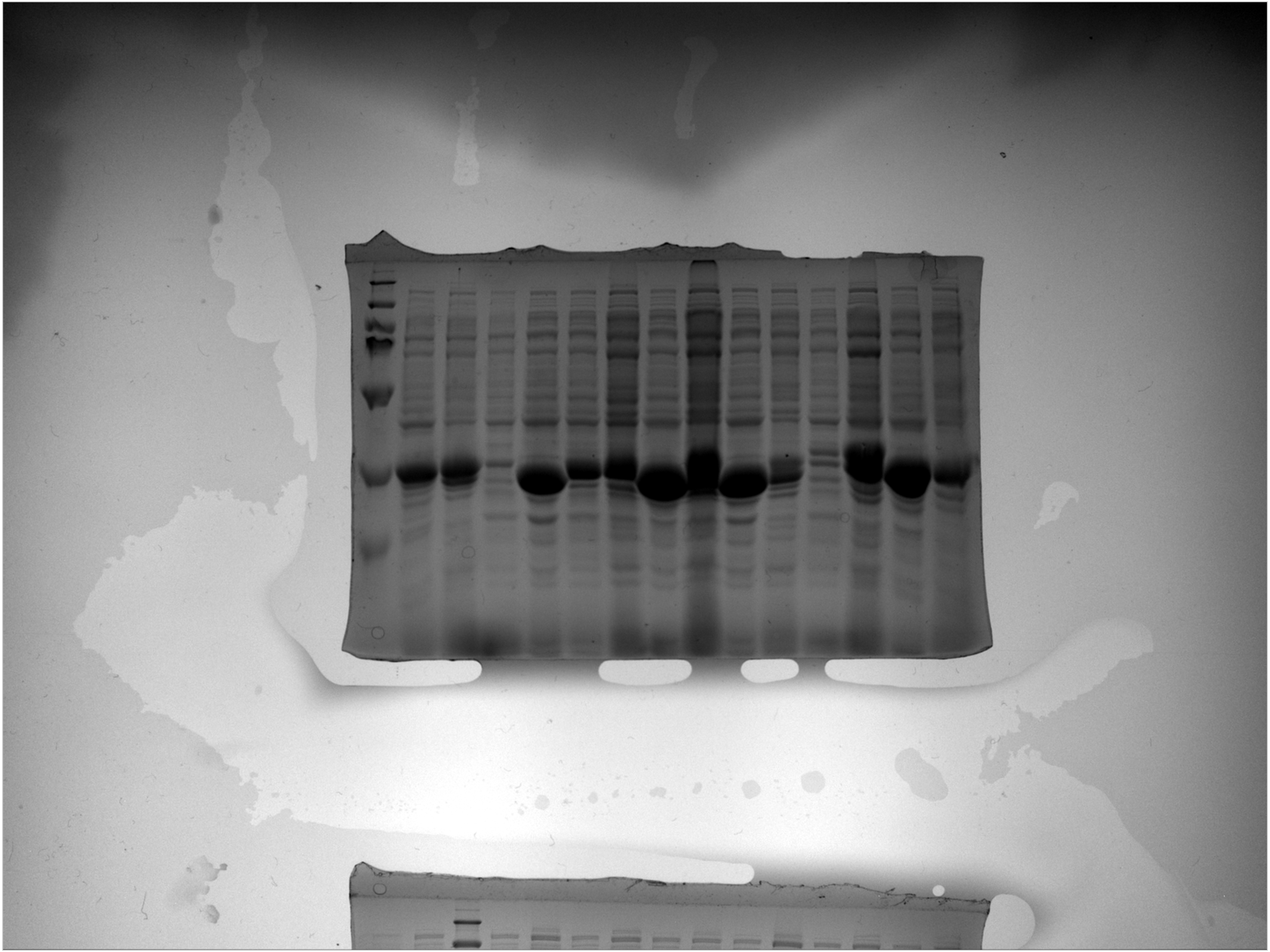

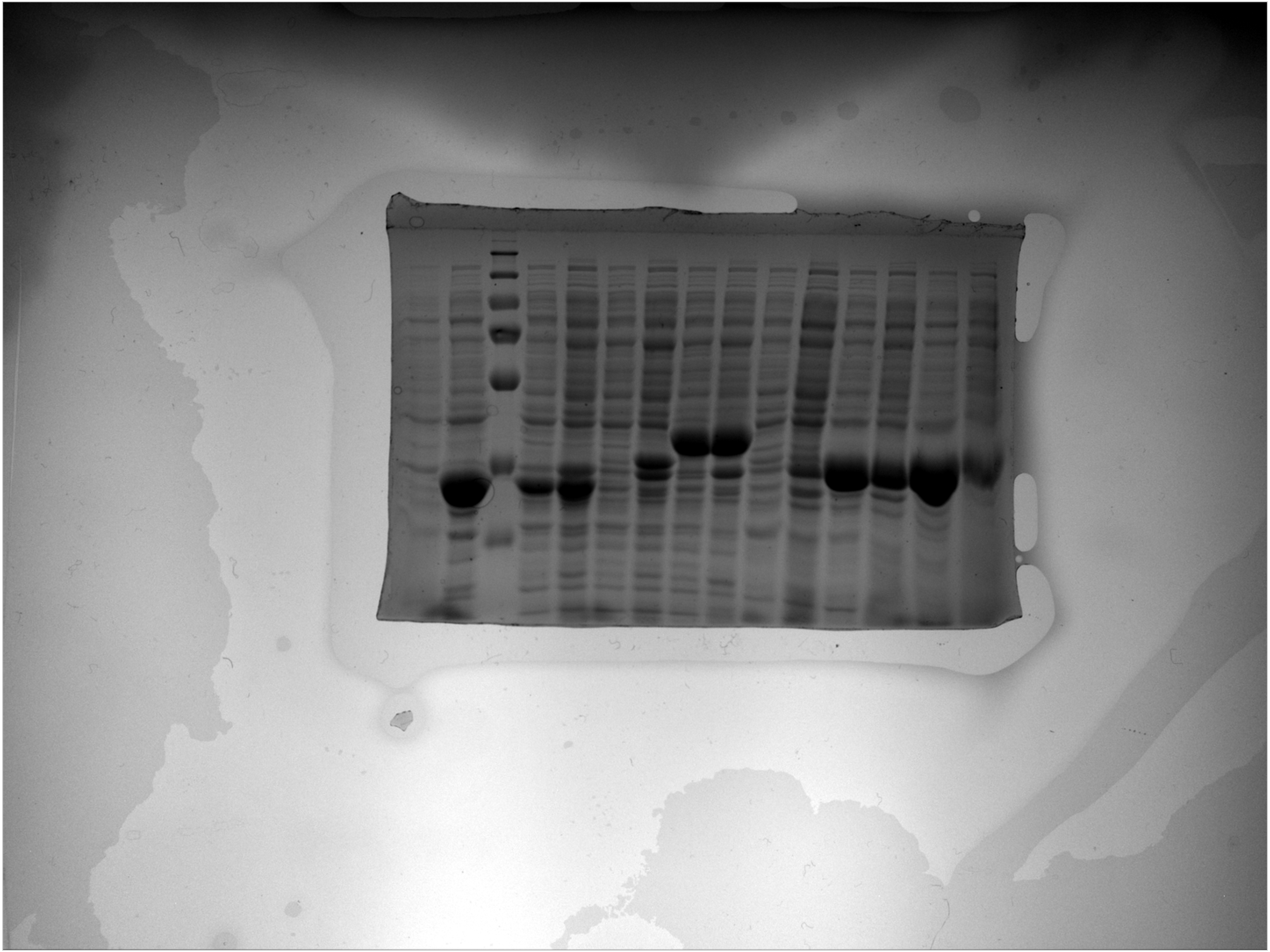

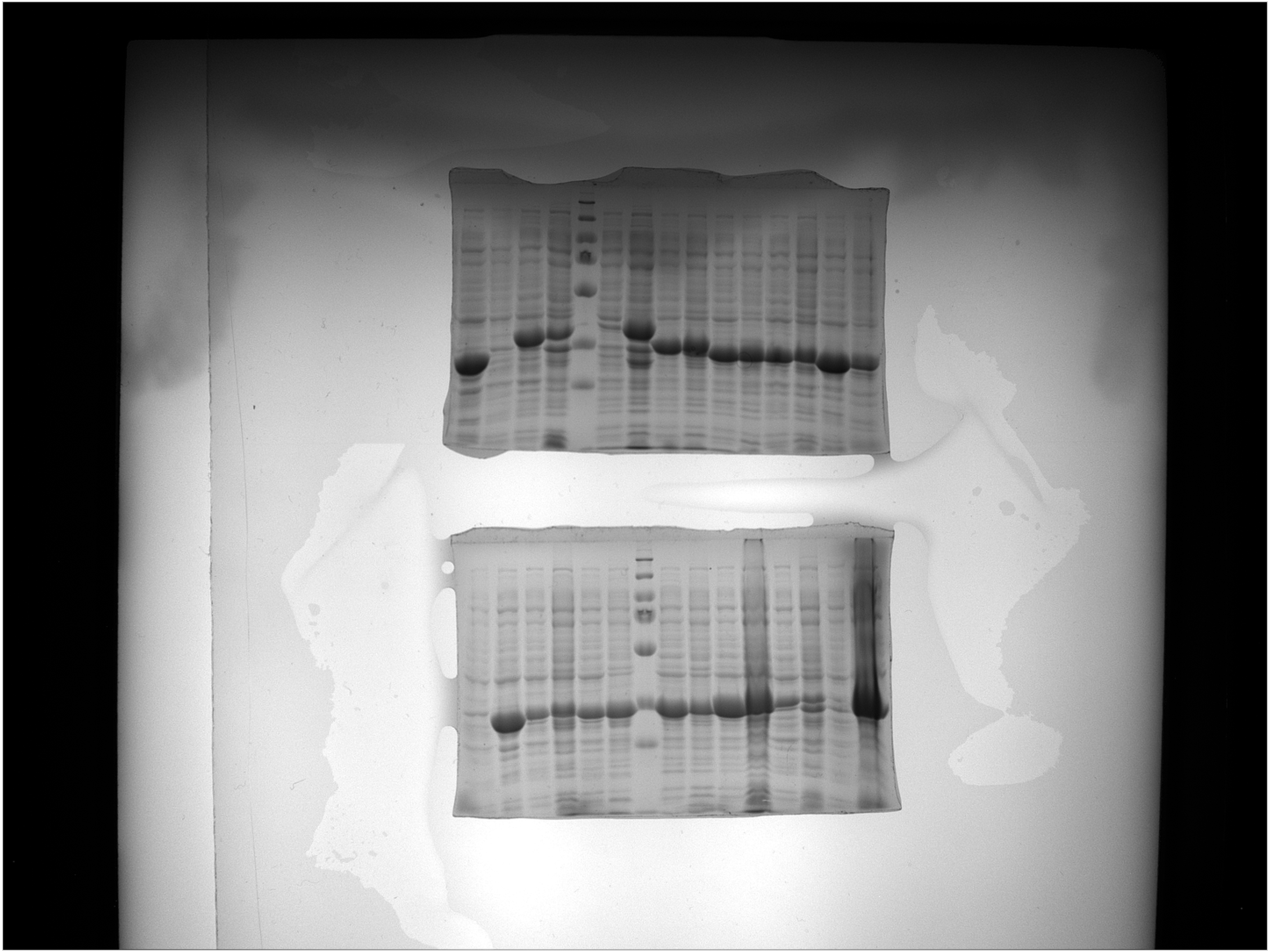

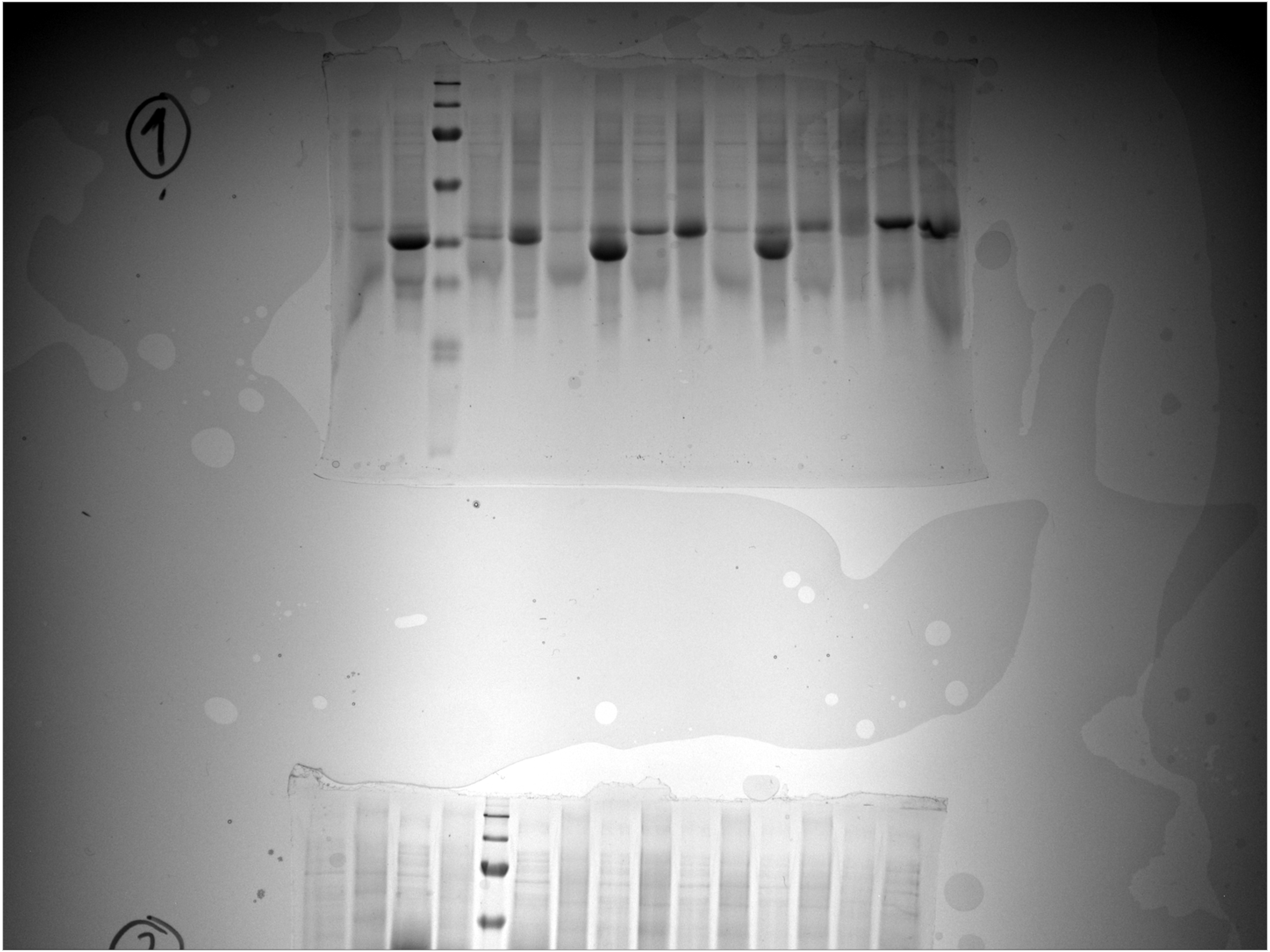
1

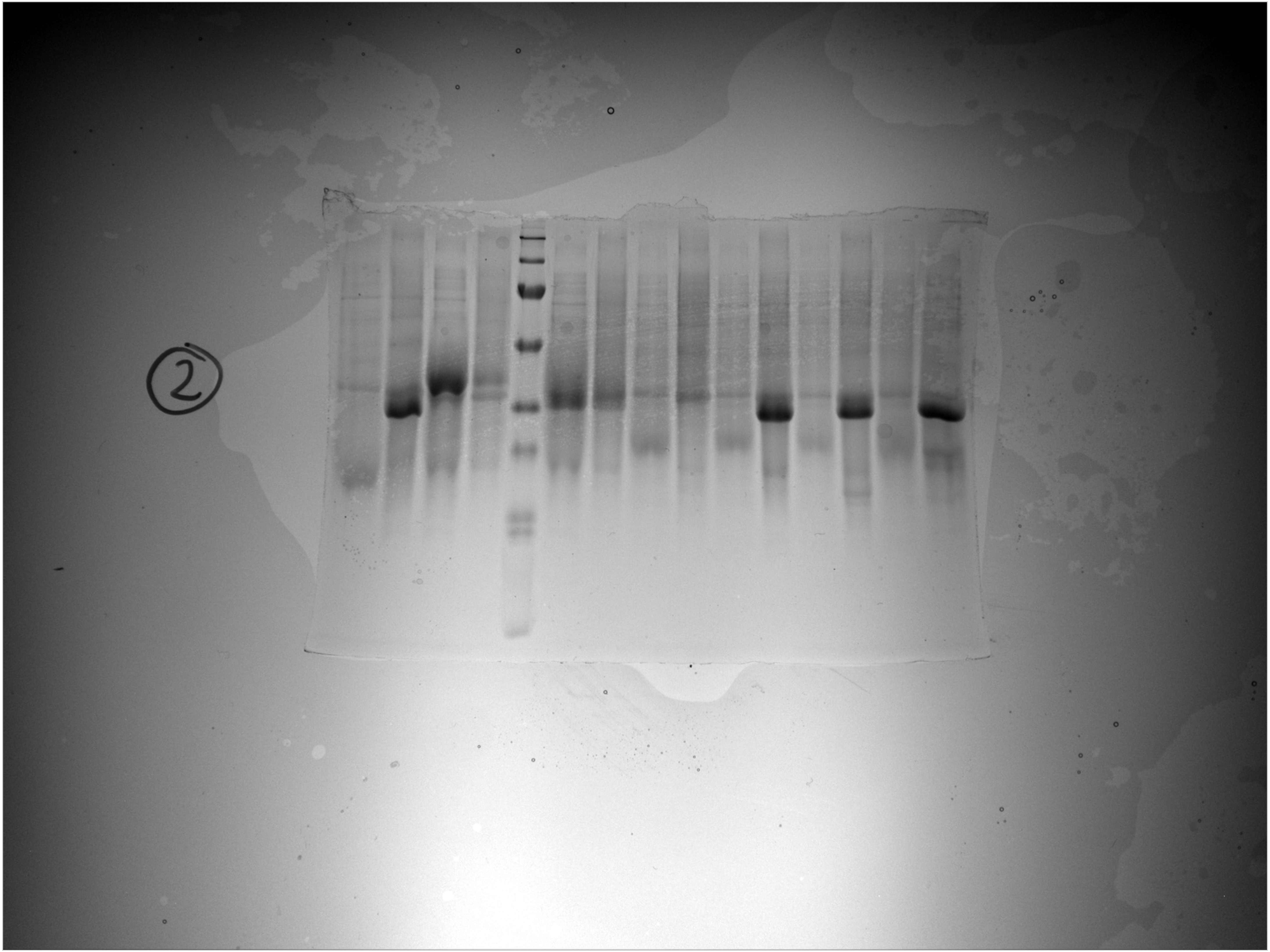
2

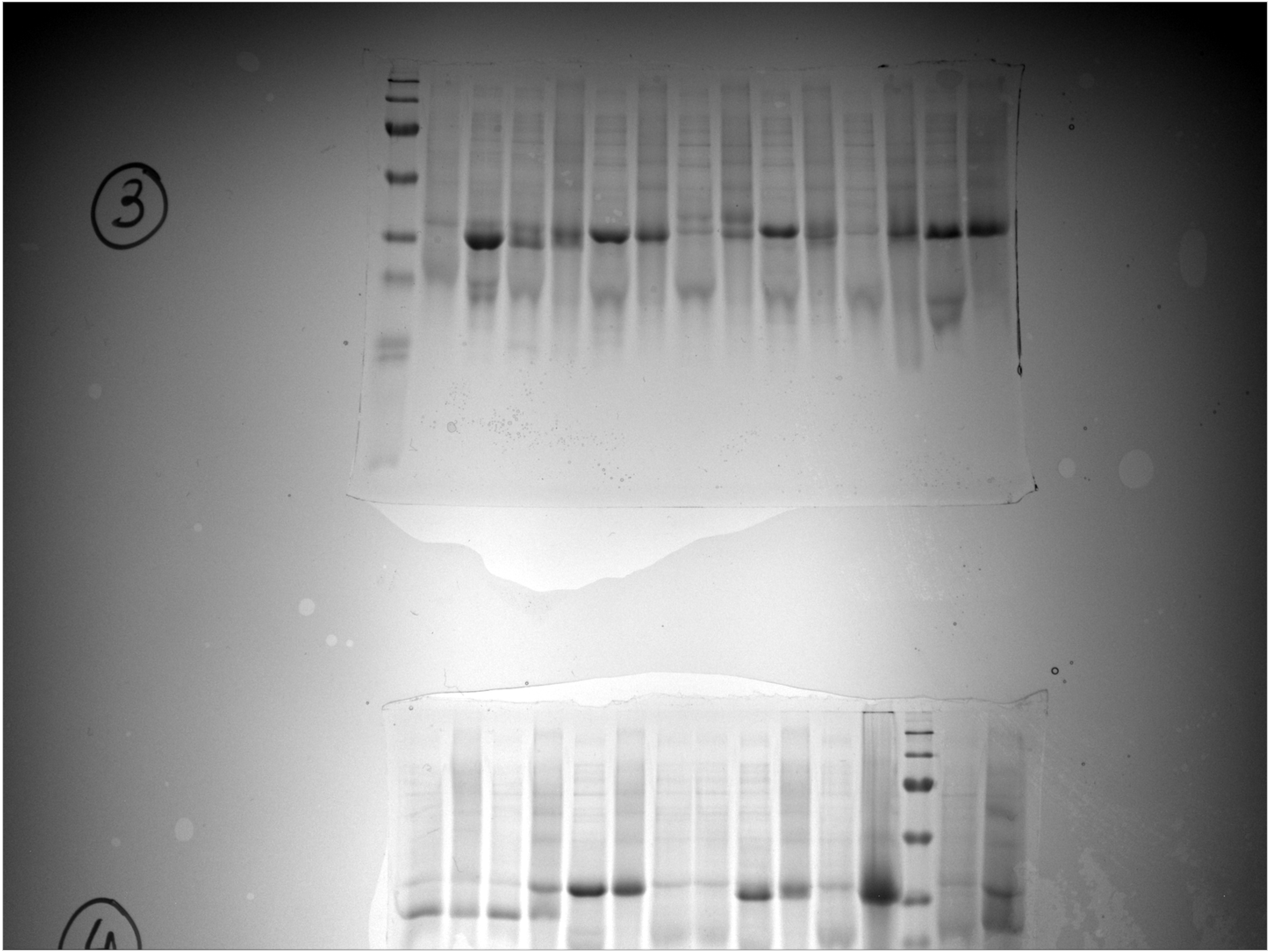
3
4

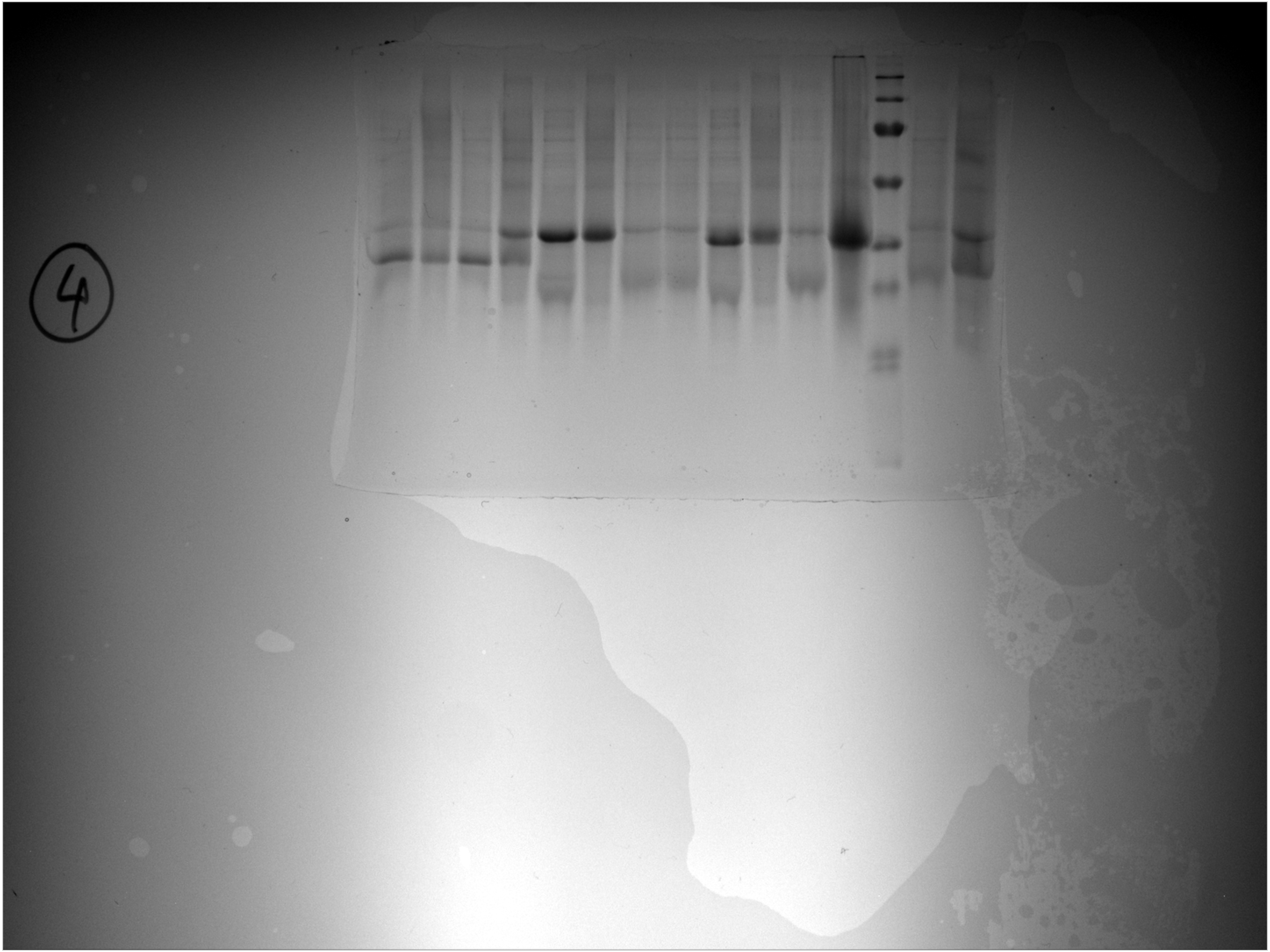
4

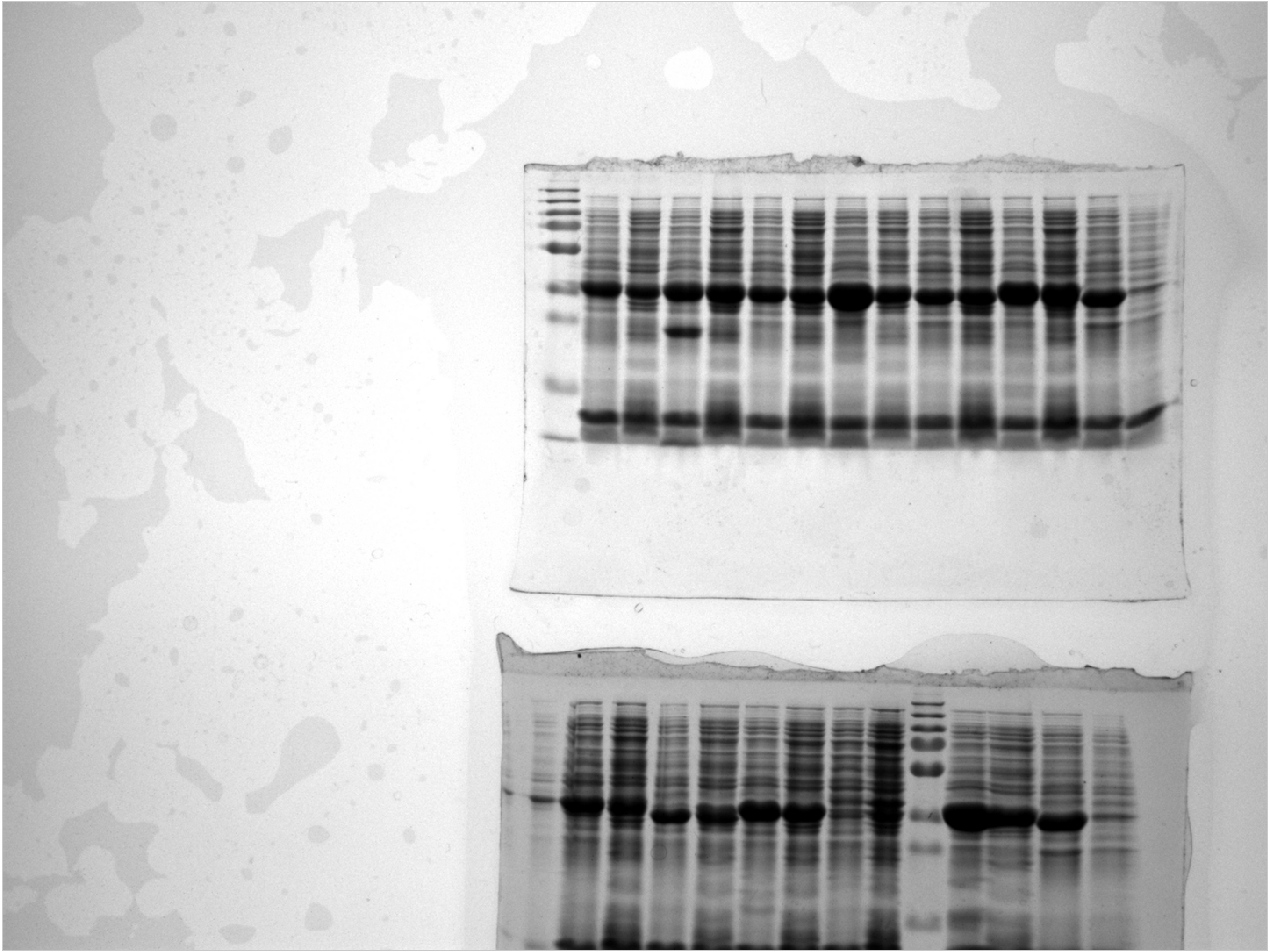

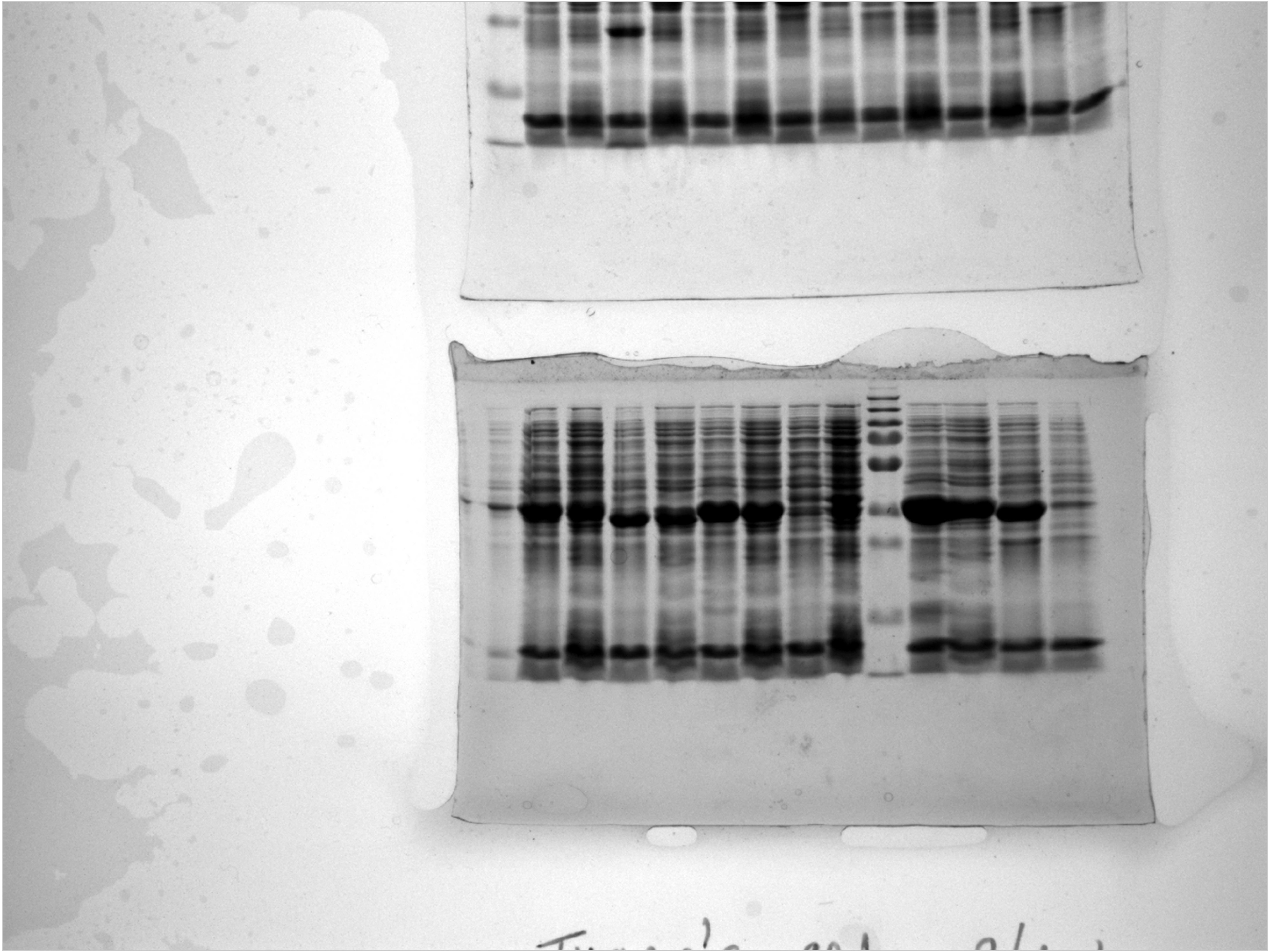

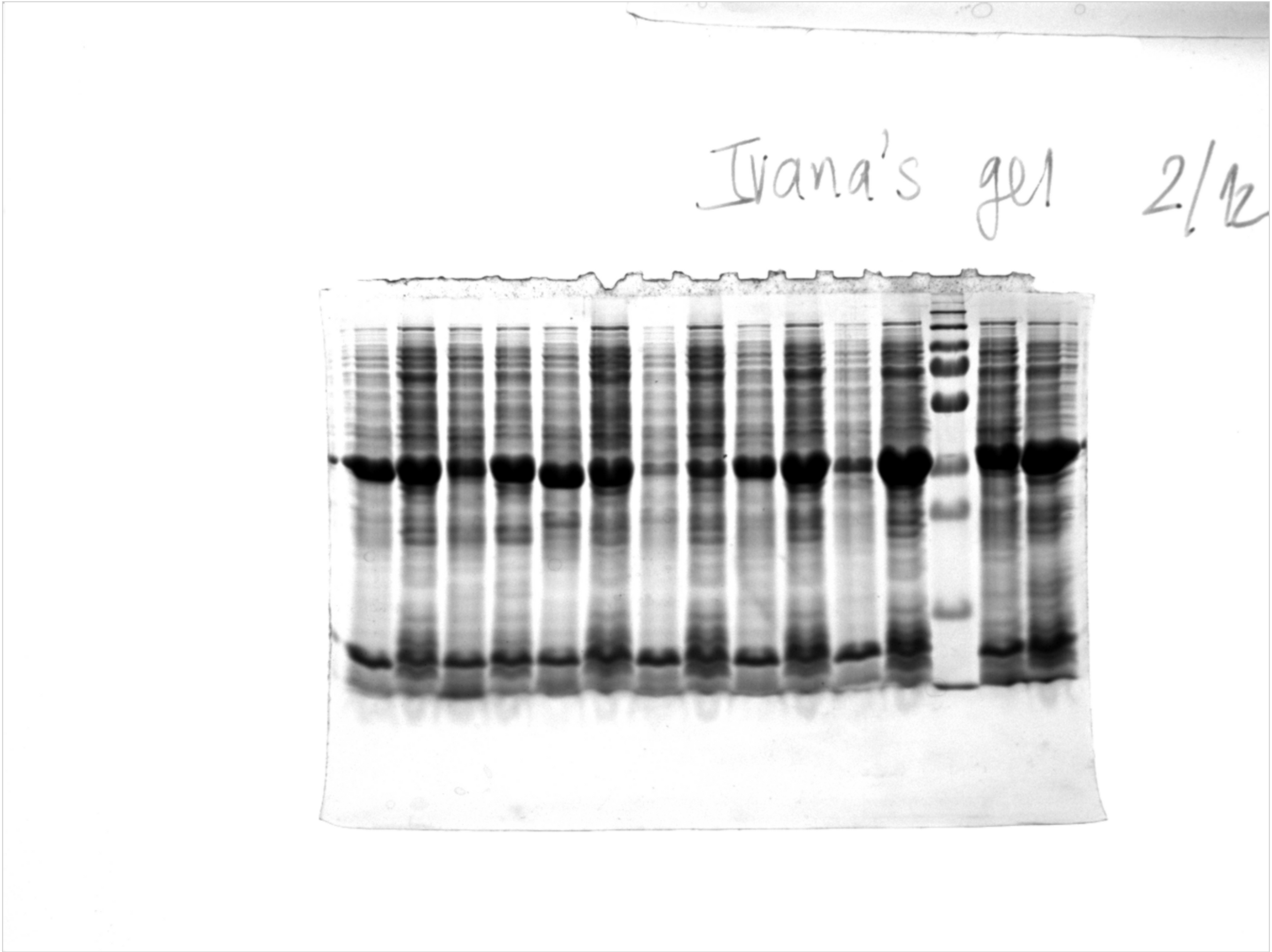
Ivana's gel 2/12

r_Scaffolds_Sca20_16deg



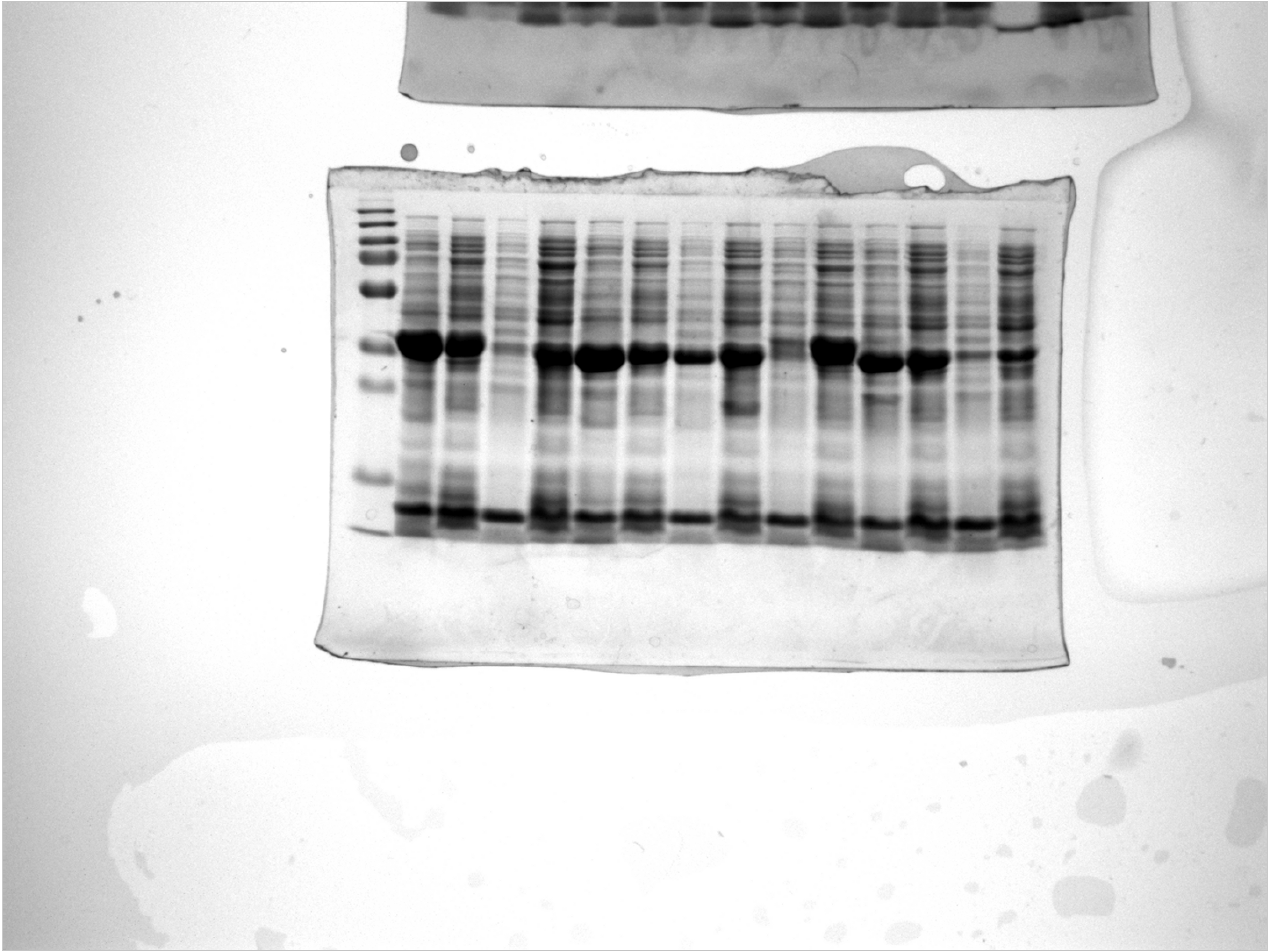

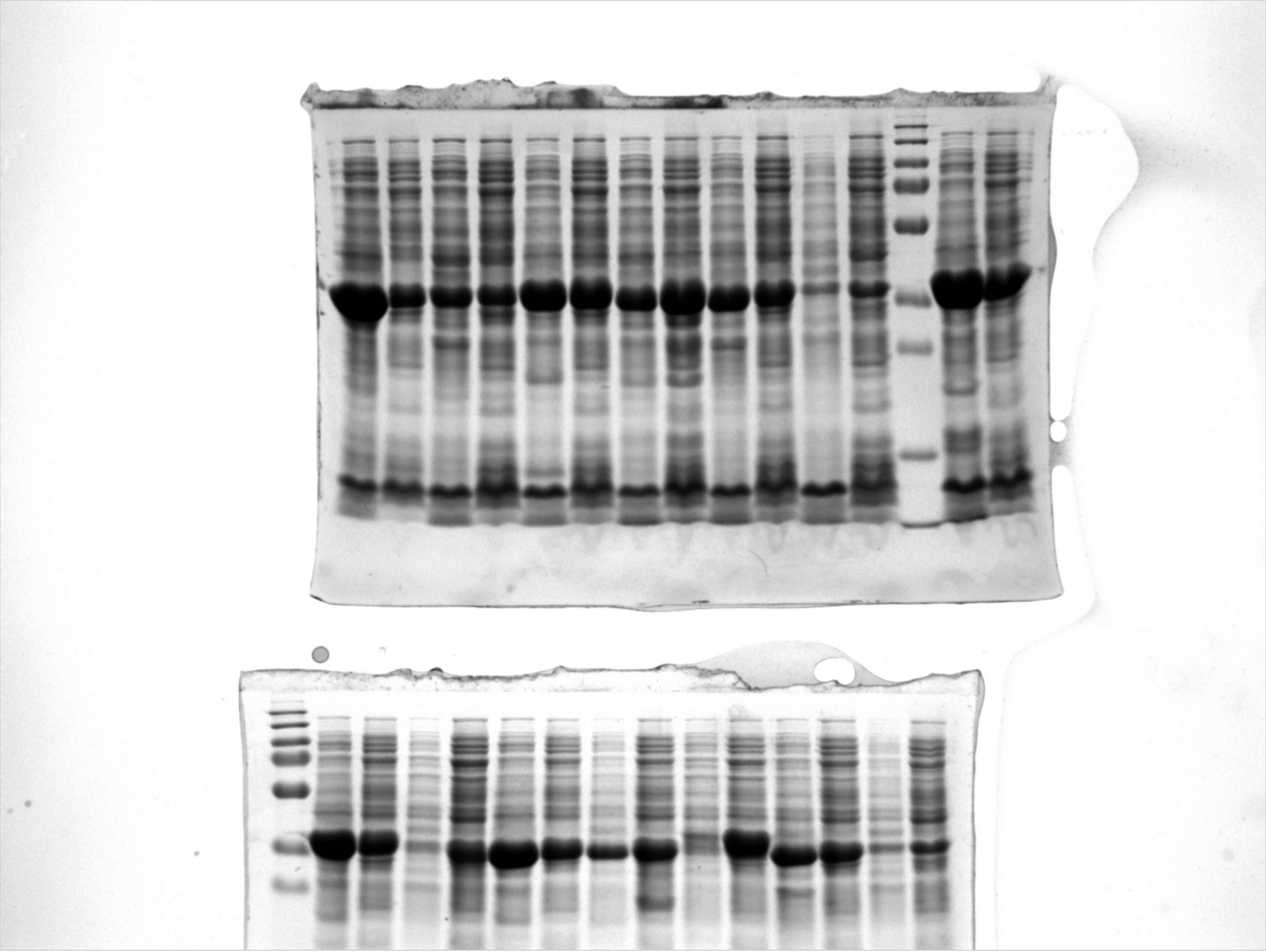

Supplement: Supplementary file 5 [file 41467_2026_77715_MOESM10_ESM.zip › Source Data for Supplementary Figure 3/SDS-PAGE_Source_Data_combined.pdf]
